# Identification of sympatric cryptic species of *Aedes albopictus* subgroup in Vietnam: new perspectives in phylosymbiosis of insect vector

**DOI:** 10.1186/s13071-017-2202-9

**Published:** 2017-06-02

**Authors:** Guillaume Minard, Van Tran Van, Florence Hélène Tran, Christian Melaun, Sven Klimpel, Lisa Katharina Koch, Khanh Ly Huynh Kim, Trang Huynh Thi Thuy, Huu Tran Ngoc, Patrick Potier, Patrick Mavingui, Claire Valiente Moro

**Affiliations:** 1 Université de Lyon, Lyon, France; 2 Université Lyon 1, Villeurbanne, France; 3 CNRS, UMR 5557, Ecologie Microbienne, Villeurbanne, France; 4 INRA, UMR1418, Villeurbanne, France; 5 Metapopulation Research Center, Department of Biosciences, University of Helsinki, Helsinki, Finland; 6 Institute for Ecology, Evolution and Diversity, Goethe-University, Frankfurt am Main, Germany; 7 Senckenberg Biodiversity and Climate Research Centre, Senckenberg Gesellschaft für Naturforschung, Frankfurt am Main, Germany; 8 Department of Medical Entomology and Zoonotics, Pasteur Institute in Ho Chi Minh City, Ho Chi Minh City, Vietnam; 9 Université de La Réunion, CNRS 9192, INSERM U1187, IRD 249, Unité Mixte Processus Infectieux en Milieu Insulaire Tropical (PIMIT), Plateforme Technologique CYROI, Sainte-Clotilde, La Réunion, France

**Keywords:** Asian tiger mosquito, Sibling species, *Wolbachia*, Microbiota, *Dysgonomonas*

## Abstract

**Background:**

The *Aedes* (*Stegomyia*) *albopictus* subgroup includes 11 cryptic species of which *Ae. albopictus* is the most widely distributed. Its global expansion associated with a documented vector competence for several emerging arboviruses raise obvious concerns in the recently colonized regions. While several studies have provided important insights regarding medical importance of *Ae. albopicus*, the investigations of the other sibling species are scarce. In Asia, indigenous populations within the *Ae. albopictus* subgroup can be found in sympatry. In the present study, we aimed to describe and compare molecular, morphological and bacterial symbionts composition among sympatric individuals from the *Ae. albopictus* subgroup inhabiting a Vietnamese protected area.

**Results:**

Based on morphological structure of the cibarial armarture, we identified a cryptic species in the forest park at Bù Gia Mập in the south-eastern region of Vietnam. Analysis of nuclear (ITS1-5.8S-ITS2) and mitochondrial (*cox*1, *nad*5) markers confirmed the divergence between the cryptic species and *Ae. albopictus*. Analysis of midgut bacterial microbiota revealed a strong similarity among the two species with a notable difference; contrary to *Ae. albopictus*, the cryptic species did not harbour any *Wolbachia* infection.

**Conclusions:**

These results could reflect either a recent invasion of *Wolbachia* in *Ae. albopictus* or alternatively a loss of this symbiont in the cryptic species. We argue that neglected species of the *Ae. albopictus* subgroup are of main importance in order to estimate variation of host-symbionts interactions across evolution.

**Electronic supplementary material:**

The online version of this article (doi:10.1186/s13071-017-2202-9) contains supplementary material, which is available to authorized users.

## Background

The *Aedes* subgenus *Stegomyia* contains currently 128 species [1]. Among them, *Aedes aegypti* and *Ae. albopictus* are of main public health concern. They are considered as main vectors for dengue and chikungunya (DENV, CHIKV) as well as Zika fever viruses, all infectious to humans. These mosquito species have also been evidenced as potential vectors under laboratory conditions for a wide range of other arboviruses including Japanese encephalitis virus, West Nile virus, eastern equine encephalitis virus and La Crosse virus. Nevertheless, the involvement of these mosquito species in the transmission of these viruses remains to be demonstrated in the field.

Identification of species within the subgenus S*tegomyia* is often based on morphological features and in particular, for adults, on patterns on the thorax (especially the scutum) and tarsi [2–5]. However, these morphological characters are not sufficient to distinguish some species, which may lead to misidentification of individuals collected in the field. This is significant for *Ae. albopictus* sibling species, combined in the literature as members of the *Scutellaris* group and *Ae. albopictus* subgroup. These species have very similar morphological characteristics especially at the larval and adult (females) stages [2–4, 6]. Furthermore, although some of the species of this subgroup have different ecological niches, some of them are found in sympatry [3, 7–9]. In Asia, indigenous populations of *Ae. albopictus* coexist with populations *of Ae. flavopictus*, *Ae. pseudalbopictus*, *Ae. subalbopictus*, *Ae. patricae*, *Ae. seatoi* and *Ae. novalbopictus* [3, 7]. In Greece, invasive populations of *Ae. albopictus* occur in sympatry with indigenous populations of *Ae. cretinus* [9]. The importance of the species of the subgroup *Ae. albopictus* in disease transmission has been poorly studied so far. This can be explained by low contact levels of these species with human populations as well as by their high resemblance to the Asian tiger mosquito, potentially leading to misidentification.

Sympatric cryptic species with recent divergences constitutes a privilege system for the understanding of symbiosis evolution. The study of symbiotic interactions is a complex and dynamic system and previous experiments have revealed strong variations in symbionts composition when comparing laboratory-reared *vs* field-caught mosquitoes but also among individuals caught in different ecosystems [10–13]. These modifications can be explained by host or symbiont population dynamics (genetic drift, bottleneck effect, expansion), modification of symbionts transmission-acquisition probability but also by modification of nutrients quality or abiotic factors that could suggest a local adaptation of one or both partners and local variation of their interaction [14–16]. The bacterial microbiota of *Ae. albopictus* presents a relative homogenous structure among populations and studies on whole body from field-caught individuals highlighted a dominance of *Wolbachia pipientis w*AlbA and *w*AlbB which often co-occur at a prevalence of ~100% [17–22]. These intracellular symbionts are involved in a control of the reproduction of *Ae. albopictus* through cytoplasmic incompatibility [23]. This process results from aberrant offspring production between infected males and uninfected females, or between hosts carrying incompatible *Wolbachia* strains. In insects, this control of reproductive process has been proposed to be the cause of reproductive isolation between populations [24]. Moreover, long term infections with *Wolbachia* and prevalence variation among populations could participate in speciation events. Similarly, reproductive isolation can be a barrier to the invasion of *Wolbachia* [25]. Studies conducted among parasitoid wasps *Nasonia giraulti* and *N. vitripennis* also showed that bacterial microbiota could be involved in speciation resulting from reproductive isolations [26]. Such events are more susceptible to occur in species complexes that have recently diverged and therefore lead to asymmetric symbionts composition regardless of their relative genetic similarity. Microbial community divergences occurring in the midgut of mosquitoes could directly impact the ecophysiology of this organ, and to a large extent the vectorial capacities of mosquitoes. Indeed, the replication of virus pathogens through the midgut constitutes the first bottleneck affecting the diversity and density of the particles [27, 28]. Furthermore, recent advances demonstrated that bacterial symbionts participate to these bottlenecks by: (i) immune priming, (ii) resource competition, and (iii) secondary metabolites production [29].

In the Nature Reserve of Bù Gia Mập in south-eastern Vietnam, we found a cryptic species living in sympatry with *Ae. albopictus*. Description of cryptic species that have recently diverged is important to decipher the recent evolution of traits in medically important vectors. We were especially interested in describing the symbiotic associations among sympatric cryptic species as variations in those associations might help to disentangle the history of symbiotic invasions as well as loss of interactions between host and symbionts. To assess its proximity to the tiger mosquito, we genotyped individuals using mitochondrial and nuclear markers and analysed their associated bacterial microbiota.

## Results

### Morphological and molecular features reveal differences among *Ae. albopictus* living in sympatry

**Fig. 1:**
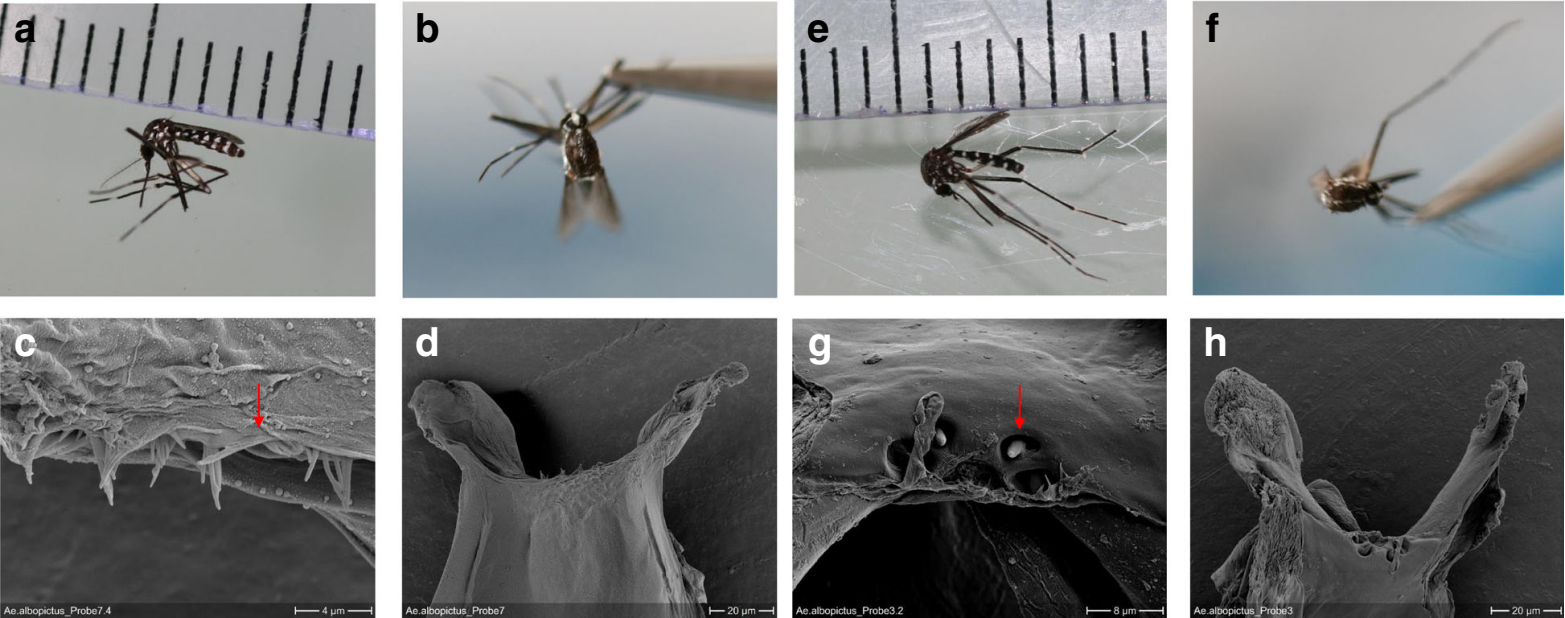
Composite figure of morphological comparison of the cryptic species *Aedes* sp. and *Ae. albopictus* from Bù Gia Mập National Park. Cryptic species *Aedes* sp.: **a** Lateral view. **b** Scutum. **c** Scanning electron microscopy of the ventral papillae. Cibarial teeth are absent (*arrow*). **d** Scanning electron microscopy of the cibarial armature including the lateral flange. *Aedes albopictus*: **e** Lateral view. **f** Scutum. **g** Scanning electron microscopy of the ventral papillae and four short cibarial teeth (*arrow*). **h** Scanning electron microscopy of the cibarial armature including the lateral fringe

**Fig. 2:**
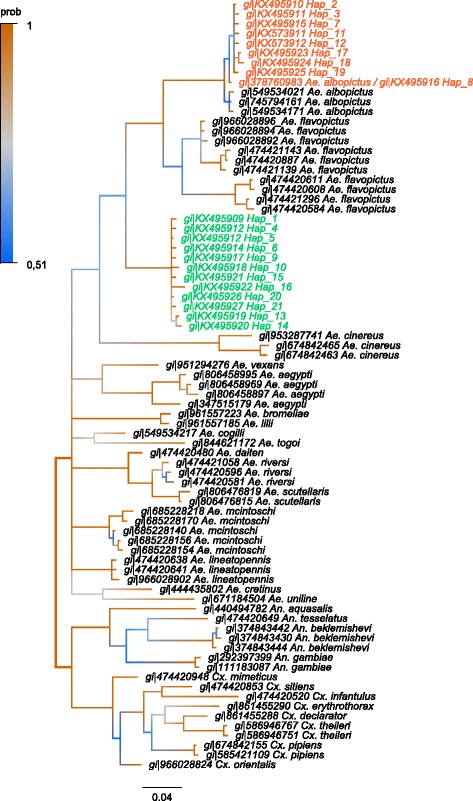
Phylogenetic tree based on *cox*1 sequences for species of the Culicidae. Bayesian consensus tree is represented. Phylogeny was built using the GTR + I + Г evolution model. Branches are coloured according to their posterior probability (prob). Clade ‘A’ associated with *Ae. albopictus* (*s.s.*) is coloured in *red*. Clade ‘C’ associated with cryptic species of *Ae. albopictus* subgroup is coloured in *green*

**Fig. 3:**
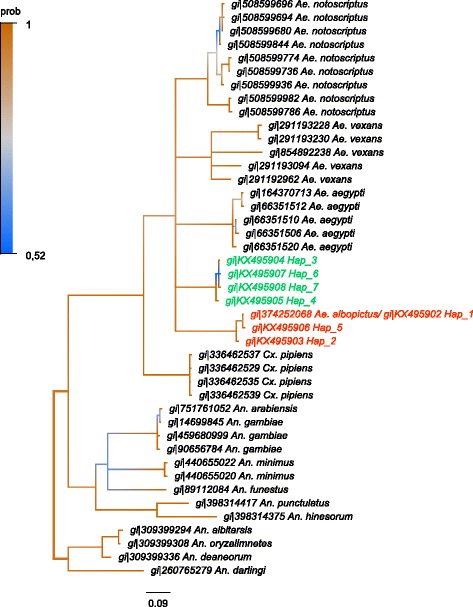
Phylogenetic tree based on *nad*5 sequences for species of the Culicidae. Bayesian consensus tree is represented. Phylogeny was built using the HKY + I + Г evolution model. Node labels refer to posterior probability of the separation. Branches are coloured according to their posterior probability (prob). Clade ‘A’ associated with *Ae. albopictus* (*s.s.*) is coloured in *red*. Clade ‘C’ associated with cryptic species of *Ae. albopictus* subgroup is coloured in *green*

**Fig. 4:**
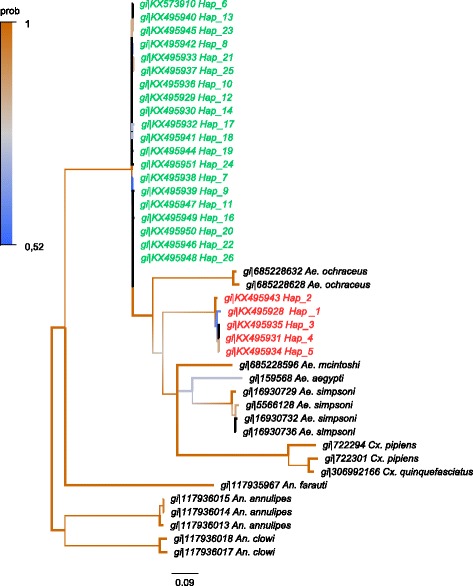
Phylogenetic tree based on ITS1-5.8S-ITS2 sequences for species of the *Culicidae*. Bayesian consensus tree is represented. Phylogeny was built using the HKY + I + Г evolution model. Node labels refer to posterior probability of the separation. Branches are coloured according to their posterior probability (prob). Clade ‘A’ associated with *Ae. albopictus* (*s.s.*) is coloured in *red*. Clade ‘C’ associated with cryptic species of *Ae. albopictus* subgroup is coloured in *green*

Adult female mosquitoes collected in the field were all identified as members of the *Ae. albopictus* subgroup using various morphological characters, especially the line of pale scale in the posterior scutum, a broad patch of white scales across lateral face of scutellum (Fig. 1). Since such morphological identification is notoriously insufficient to separate adult females within the *Ae. albopictus* subgroup [2, 3, 7]*,* we completed the identification using molecular markers. The phylogenetic distances between individuals were estimated through two mitochondrial (*cox*1, *nad*5) and three nuclear (ITS1-5.8S-ITS2 rRNA) barcodes over 43 individuals (Table 1). For technical reason 48 individuals have been haplotyped with the *cox*1 marker, 44 of those were also haplotyped with the ND5 marker, and 25 of them were genotyped with ITS1-5.8S-ITS2 marker. Individuals displaying genetically close barcodes were clustered under a unique reference haplotype number (Table 2), leading to a total of 21, 7 and 25 different reference haplotypes using *cox*1, *nad*5 and ITS1-5.8S-ITS2 markers, respectively. Distances among reference haplotypes estimated with Kimura 2-parameter (K2P) were highly correlated between the different barcodes (*cox*1 - ITS1-5.8S-ITS2, *r*_(274)_ = 0.99, *P* < 2.2.10^-16^; *cox* 1 - *nad*5, *r*_(701)_ = 0.99, *P* < 2.2.10^-16^; *nad*5 - ITS1-5.8S-ITS2, *r*_(204)_ = 0.99, *P* < 2.2.10^-16^) (Additional file 1: Figure S1). Two congruent monophyletic groups were identified subsequently and assigned to two different species (Table 2, Figs. 2, 3 and 4). The two clades were called ‘A’ and ‘C’ and corresponded respectively to the species *Ae. albopictus* and to a non-characterized cryptic species hereafter referred as *Aedes* sp. Both taxa could be discriminated using polymorphism at ITS1-5.8S-ITS2 locus. The individuals associated to clade A had an amplicon size > 1150 bp whereas those associated with clade C had an approximate amplicon size < 1050 bp, allowing easy and accurate identification of both clades through 1% agarose gel electrophoresis (Additional file 1: Figure S2). It is however important to mention that this PCR could lead to an additional band of < 560 bp in *Ae. albopictus* depending on the presence of the symbiotic protist *Ascogregarina taiwannensis*, which did not interfere with the ITS-based identification of A and C. Among those three markers, individuals from the clade C presented an equivalent or slightly higher haplotype diversity (Hd*cox*1 = 0.74; Hd*nad*5 = 0.23; HdITS1-5.8S-ITS2 = 1) than the individuals from the clade A (Hd*cox*1 = 0.70; Hd*nad*5 = 0.20; HdITS1-5.8S-ITS2 = 1). Two *cox*1 sequences annotated as *Ae. albopictus* but close the the clade C were found in the public databases (Additional file 1: Supplementary informations). In order to improve detection methods for *Aedes* sp., observation of cibarial armarture through scanning electron microscopy was used to highlight any morphological difference between members of clades A and C. Three morphological differences were characterized for both species: (i) a structural difference in the ventral papillae; (ii) a difference in the angle of the lateral flange; and (iii) the cibarial teeth. While all ventral papillae in *Ae. albopictus* become continuously slimmer from the base to tip, the ones in *Aedes* sp. from Bù Gia Mập show a bulbous thickening before the tip; the angle of the lateral flanges is wider in the cibarium of *Aedes* sp. from Bù Gia Mập than in the one of *Ae. albopictus*; cibarial teeth are absent in *Aedes* sp. from Bù Gia Mập, while *Ae. albopictus* has four short cibarial teeth (Fig. 1). Therefore, those morphological keys can be used to differentiate the two species.

**Table 1:** Samples information

	Number of tested individuals (*Ae. albopictus/*cryptic *Aedes* sp.)
Barcoding and *Wolbachia* detection	43 (17/26)
b-ARISA	24 (9/15)
16SrDNA MiSeq sequencing	5 (3/2)
Total	72 (29/43)

**Table 2:** Individual’s haplotypes and *Wolbachia* infection status

Sample reference	*cox*1 clade (Haplotype)	*nad*5	ITS1-5.8S-ITS2	*Wolbachia* - *wsp*
1	Clade A (Hap_2)	Clade A (Hap_1)	–	positive
2	Clade A (Hap_2)	Clade A (Hap_1)	–	positive
3	Clade A (Hap_2)	Clade A (Hap_1)	Clade A (Hap_1)	positive
4	Clade C (Hap_1)	Clade C (Hap_3)	Clade C (Hap_12)	negative
5	Clade C (Hap_5)	Clade C (Hap_3)	Clade C (Hap_14)	negative
6	Clade A (Hap_2)	–	Clade A (Hap_4)	positive
7	Clade C (Hap_9)	Clade C (Hap_3)	Clade C (Hap_17)	negative
8	Clade C (Hap_5)	Clade C (Hap_3)	Clade C (Hap_21)	negative
9	Clade A (Hap_7)	Clade A (Hap_1)	Clade A (Hap_5)	positive
10	Clade A (Hap_8)	Clade A (Hap_2)	–	positive
11	Clade A (Hap_2)	Clade A (Hap_1)	–	positive
12	Clade A (Hap_2)	Clade A (Hap_1)	Clade A (Hap_3)	positive
13	Clade A (Hap_2)	Clade A (Hap_5)	–	positive
14	Clade A (Hap_7)	Clade A (Hap_1)	–	positive
15	Clade C (Hap_6)	Clade C (Hap_3)	–	negative
16	Clade C (Hap_5)	Clade C (Hap_3)	Clade C (Hap_10)	negative
17	Clade A (Hap_2)	Clade A (Hap_1)	–	positive
18	Clade C (Hap_5)	Clade C (Hap_3)	Clade C (Hap_25)	negative
19	Clade C (Hap_5)	Clade C (Hap_3)	Clade C (Hap_7)	negative
20	Clade C (Hap_10)	Clade C (Hap_6)	Clade C (Hap_9)	negative
21	Clade A (Hap_3)	Clade A (Hap_1)	–	positive
22	Clade C (Hap_4)	–	Clade C (Hap_13)	negative
23	Clade A (Hap_12)	Clade A (Hap_1)	–	positive
24	Clade C (Hap_5)	Clade C (Hap_3)	–	negative
25	Clade C (Hap_5)	Clade C (Hap_3)	Clade C (Hap_6)	negative
26	Clade C (Hap_5)	Clade C (Hap_3)	Clade C (Hap_8)	negative
27	Clade C (Hap_5)	Clade C (Hap_3)	–	negative
28	Clade A (Hap_18)	Clade A (Hap_1)	Clade A (Hap_2)	positive
29	Clade A (Hap_19)	Clade A (Hap_1)	–	positive
30	Clade C (Hap_20)	–	Clade C (Hap_19)	negative
31	Clade C (Hap_5)	Clade C (Hap_3)	Clade C (Hap_23)	negative
32	Clade C (Hap_21)	Clade C (Hap_7)	–	negative
33	Clade C (Hap_13)	Clade C (Hap_3)	Clade C (Hap_18)	negative
34	Clade C (Hap_13)	Clade C (Hap_3)	Clade C (Hap_22)	negative
35	Clade C (Hap_13)	Clade C (Hap_3)	–	negative
36	Clade C (Hap_14)	Clade C (Hap_3)	–	negative
37	Clade C (Hap_13)	–	Clade C (Hap_11)	negative
38	Clade C (Hap_5)	Clade C (Hap_3)	Clade C (Hap_26)	negative
39	Clade A (Hap_2)	Clade A (Hap_1)	–	positive
40	Clade C (Hap_15)	Clade C (Hap_3)	Clade C (Hap_16)	negative
41	Clade C (Hap_5)	Clade C (Hap_3)	Clade C (Hap_20)	negative
42	Clade C (Hap_16)	Clade C (Hap_4)	Clade C (Hap_24)	negative
43	Clade A (Hap_17)	Clade A (Hap_1)	–	positive
BGM1^a^	Clade A (Hap_2)	Clade A (Hap_1)	–	–
BGM3^a^	Clade A (Hap_2)	Clade A (Hap_1)	–	–
BGM4^a^	Clade A (Hap_11)	Clade A (Hap_1)	–	–
BGM5^a^	Clade C (Hap_5)	Clade C (Hap_3)	–	–
BGM6^a^	Clade C (Hap_5)	Clade C (Hap_3)	–	–

^a^Samples used for microbiota analysis

### *Wolbachia* are naturally present in *Ae. albopictus* but absent in the cryptic species

Among all individuals from clade A, assigned to *Ae. albopictus*, all tested individuals (17/17) were diagnosed positive for *Wolbachia* infection *via* amplification of the *wsp* gene (Additional file 1: Table S1). On the contrary, no *Wolbachia* (0/26) were detected in the individuals from clade C (cryptic *Aedes* sp.).

### Midgut bacterial microbiota are similar between *Ae. albopictus* and the cryptic species

**Fig. 5:**
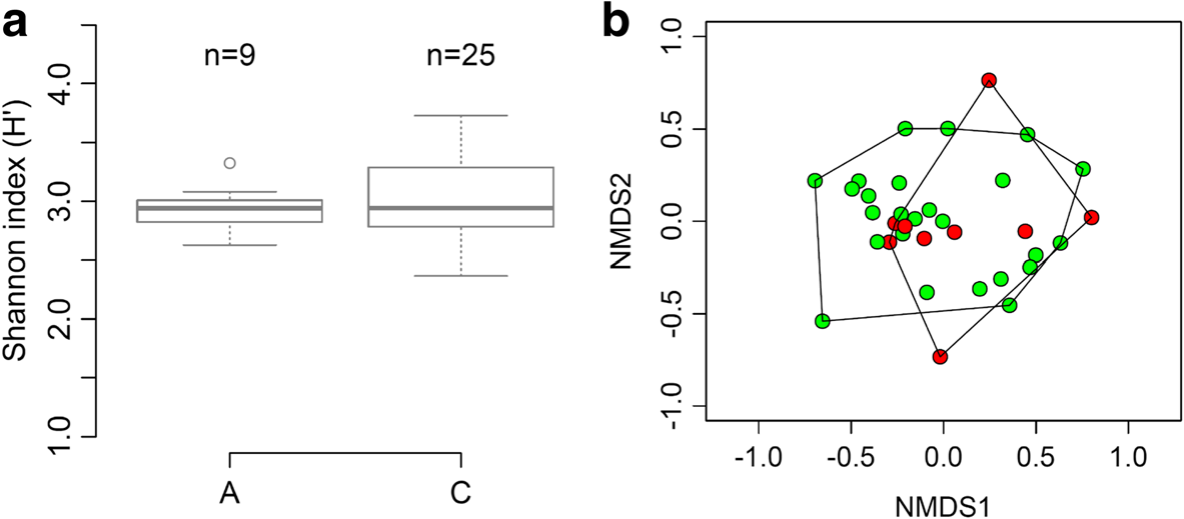
Comparison of bacterial diversity between individuals of the two *Ae. albopictus* cryptic species. **a** Boxplot representation of Shannon α-diversity within individuals associated to the ‘A’ clade (*Ae. albopictus*) and the ‘C’ clade (cryptic species). **b** 2D non-metric multidimensional sequence scaling representing dissimilarity distances among individuals of the ‘A’ clade in *green* and the ‘C’ clade in *red*

**Fig. 6:**
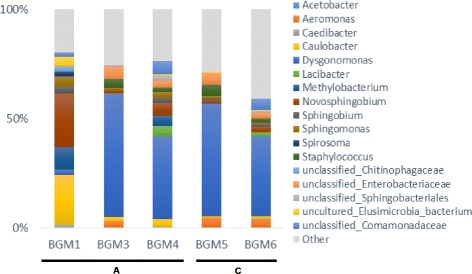
Bacterial composition of the midgut of the two *Ae. albopictus* cryptic species. The proportion of each taxon refers to the proportion of sequences identified through V5-V6 regions of 16S rDNA sequenced with MiSeq. The taxa names refer to assignation of 3% distance OTUs inside the samples. The taxa, which represented less than 1% of the sequences, were classified as “other”. Five individuals were analysed and belonged respectively to the ‘A’ clade (*Ae. albopictus*) or the ‘C’ clade (cryptic species)

b-ARISA analysis of mosquito midgut microbiota was performed on a total of 24 individuals. Using ITS1-5.8S-ITS2 polymorphism presented above, a total of 9 individuals were assigned to the A clade of *Ae. albopictus* species and 15 individuals to the cryptic *Aedes* sp. species (i.e. clade C). The Shannon α-diversity index was equivalent between both species (Mann-Whitney-Wilcoxon, *U*_(34)_ = 102, *Z* = -0.04, *P =* 0.7) (Fig. 5a). Computing Bray-Curtis dissimilarity distance, no divergence in bacterial community structure was observed between the two species (adonisANOVA, *F*_(1,32)_ = 1.02, *P =* 0.37) (Fig. 5b). In order to assess the taxonomic composition of bacterial community between the two species, a metabarcoding analysis was performed on a set of 5 additional individuals. The seventeen dominant operational taxonomic units (OTUs), which represented more than 1% of the overall rarefied sequences were represented (Fig. 6). Interestingly, the dominant taxa were similar between cryptic specimens and *Ae. albopictus* specimens. *Dysgonomas* sp., as well as members of the family *Sphingomonadaceae* (*Sphingobium, Novosphingobium, Sphingomonas*) represented more than 35% of the sequences among the individuals (Additional file 1: Table S2).

## Discussion

To the best of our knowledge, in this study we report for the first time the presence of a phylogenetically divergent cryptic species living in sympatry with *Ae. albopictus* in a protected forest of the Binh Phuoc Province in Vietnam. For convenience, no name has been given to this species. Since no mating experiments have been performed, further investigations would be required to confirm the reproductive isolation of the two species. We used several markers (barcodes, amplicon length, cibarial armature morphology) that could be used later to discriminate the cryptic species from *Ae. albopictus* (*sensu stricto*). These markers constitute a useful tool for entomological studies as we report previous inconsistent and presumably wrong assignation of this species among *Ae. albopictus* subgroup based solely on *cox*1 barcodes (Additional file 1: Supplementary information). Using similar approaches, previous investigations on *Anopheles* malaria vectors highlighted the presence of various cryptic species in Vietnamese national parks [30]. Characterization of vector species is a prerequisite to estimate pathogens transmission risks. In South-East Asia, identification of *Aedes* mosquitoes remains of main concern because of their ability to replicate and retransmit viral pathogens including dengue or Japanese encephalitis viruses. The Province of Binh Phuoc in Vietnam has often been identified as an advanced region for dengue transmission suggesting that this location could be a centroid of annual epidemics [31]. Two main identified vectors are involved in replication and transmission of this virus in South-East Asia: *Ae. aegypti* and *Ae. albopictus.* Of the latter, various cryptic species occur in that region [3, 7, 8]. However, the ecology of these species is partially unknown, at least in part, because of diagnosis difficulties. In addition to their epidemiological interest, we argue that studies on the ecology of sympatric species sharing a recent last common ancestor constitute a privileged system for the understanding of evolutionary processes. Symbiosis, in particular is a complex investigation field as the nature of interactions between host and microbes can drastically change across evolution depending on changes in: (i) trophic interactions; (ii) symbionts transmission pathways among hosts; and (iii) coevolution.

In this study, we were not able to detect any *Wolbachia* infection in the cryptic species of the *Ae. albopictus* complex. The difference in infection status between these related species is interesting as *Wolbachia-*induced sexual isolation could occur before hybrid lethality and therefore participate to speciation events [24]. In addition to their consistent divergence, individuals from the clade C lacking *Wolbachia* infections presented a slightly higher haplotype diversity relative to these from the clade A. Several examples in the *Culex pipiens* complex witnessed an asymmetric invasion by *Wolbachia* within cryptic species [32–34]. Indeed, non-invaded populations of *Cx. pipiens* from South Africa harbored an ancestral and higher mitochondrial diversity [32]. This reduction of organelle genetic diversity often occurs after *Wolbachia* invasion due to mitochondrial hitchhiking successive to the rapid spread of the symbiont [35]. Such mitochondrial sweep was not supported by our data as no significant differences in the haplotypes diversities were observed between *Ae. albopictus* and the cryptic species. A recent study, conducted by Dumas et al. [34] highlighted a similar divergence across nuclear and mitochondrial genomes when comparing uninfected and infected *Cx. pipiens* populations. This nuclear divergence supported the hypothesis of a reproductive isolation among those sympatric individuals that did not present any gene flow among them [34]. Similarly, a haplotype-based study of fig wasp *Eupristina verticillata* conducted within two Chinese provinces demonstrated that morphologically similar individuals diverged in three cryptic species [36]. These observations were supported with both mitochondrial and nuclear markers and were correlated with the *Wolbachia* clades infecting them. The authors suggested that a reproductive manipulation induced by *Wolbachia* might have been responsible for the observed speciation. Such patterns contrast with *Wolbachia* related-genetic divergences that were observed in lepidopterans *Phengaris teleius* and *P. nausithious* [37]. Within these two species, individuals infected by *Wolbachia* presented a mitochondrial divergence without consistent nuclear genetic structure. Such cryptic speciation mimicry might be induced by a mitochondrial hitchhiking without any gene flow barrier preventing breeding of infected and uninfected individuals. The pattern observed in our study supported a mitochondrial and nuclear divergence among the two sympatric A and C clades, which is consistent with a cryptic speciation. Similarly to the previous examples, our results do not inform whether a *Wolbachia*-independent sexual isolation also occurred. Mating experiments of cryptic species with *Wolbachia* infected or uninfected lines could enable us to estimate whether hybrid lethality or *Wolbachia*-induced sexual isolation occurred between these species.

In addition, our b-ARISA analysis showed that *Ae. albopictus* midgut microbiota does not differ from that of the cryptic species. MiSeq sequencing of 16S rDNA variants of 3 individuals of *Ae. albopictus* and 2 individuals of the cryptic species identified *Dysgonomonas* and *Sphingomonadaceae* as potential dominant taxa of the bacterial microbiota*.* The bacterial composition of *Ae. albopictus* midgut is strongly different from the rest of its body [22]. Investigating midgut microbiota of seven populations from France and Vietnam, we previously discovered a similar structure across populations with the presence of the same dominant genus *Dysgonomonas* sp. [22]. Individuals sampled in Vietnam also showed an enrichment of several taxa including *Sphingomonadaceae* (*Sphingobium, Sphingomonas, Novosphingobium*). Most of these similar bacterial taxa are also found in the water and plants with which mosquitoes are in contact during immature and adult stages [38, 39]. A recent study on the *Litoditis marina* complex (Nematoda) described a divergent structure between the bacterial microbiota among three cryptic species [40]. The authors suggested a link between the microbiota, intrinsic physiological properties and trophic interactions specificities for each species. Contrary to these results, the similarity between midgut bacterial microbiota associated with the two species of the *Ae. albopictus* complex revealed here, suggests similar physiology and trophic interactions with plants and vertebrate’ hosts. However, further investigations would be needed in order to properly describe the taxonomic composition of the bacterial communities associated with the midgut of *Ae. albopictus* cryptic species compared to that of *Ae. albopictus*.

## Conclusion

The identification of a cryptic species of *Ae. albopictus* in Vietnamese forest was evidenced by the detection of genetically divergent and morphologically similar individuals. New morphological keys based on cibarial armature and papillae were proposed for the distinction of this species from *Ae. albopictus* (*s.s*). Intestinal microbiota of both species were equivalent suggesting a similar selection - acquisition of their digestive symbionts. However, both species differed by their *Wolbachia* infection status. The two species present a promising study system to investigate the relationship between symbionts and vector insects evolving in equivalent ecosystems.

## Methods

### Study site and mosquito collection

**Fig. 7:**
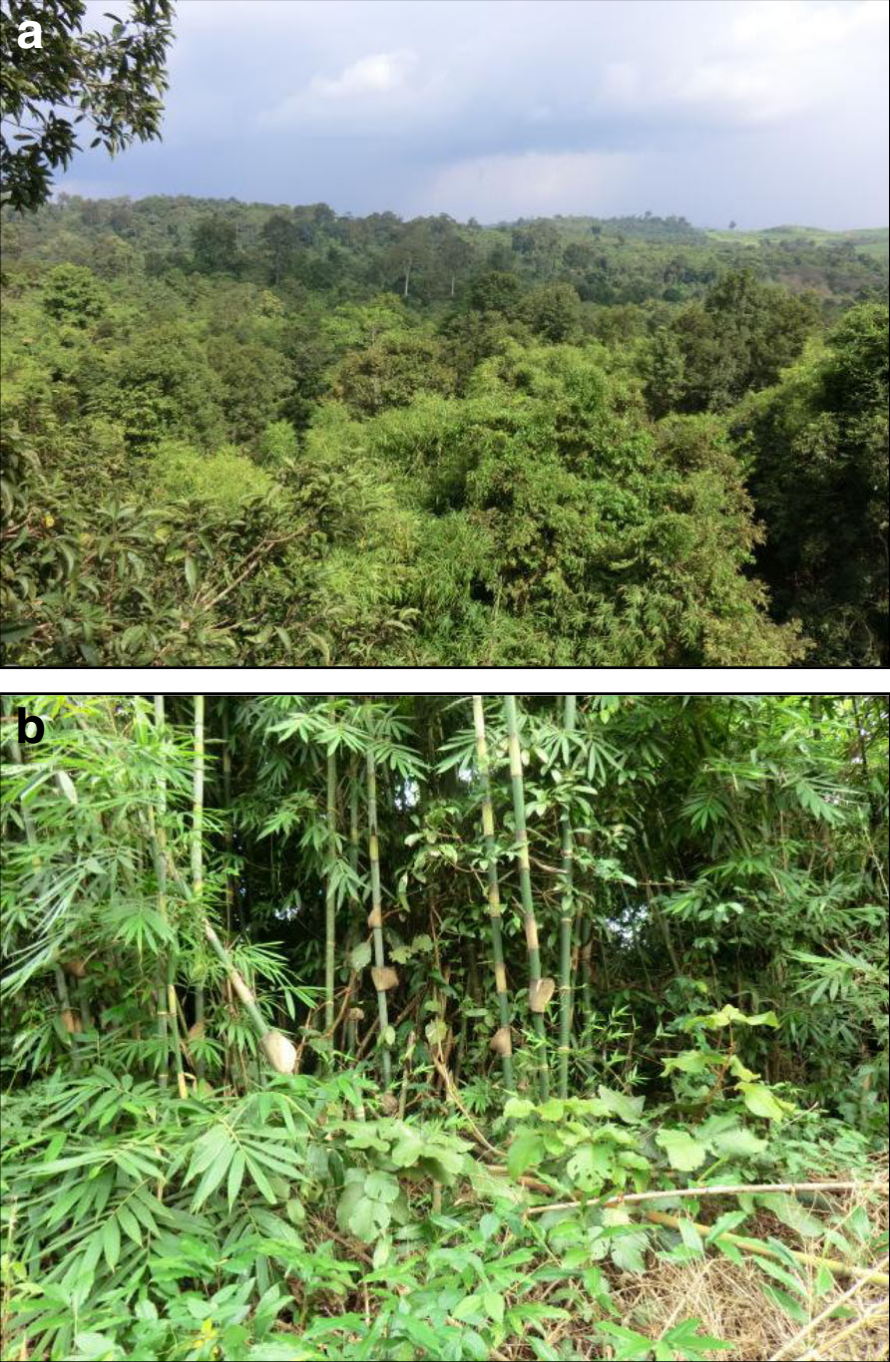
Bù Gia Mập National Park. **a** Canopy of the protected forest. **b** Breeding site where *Ae. albopictus* mosquitoes were identified and collected

*Aedes* specimens were sampled during October 2012 in Vietnam in a protected forest of Bù Gia Mập National Park located in the province of Binh Phuoc (12°6′42″N, 107°9′29″E according to the World Geodetic System 1984). After several investigations in different habitats of the site, adults of *Aedes* spp. were only found at the edge of the primary forest inside the protected area (Fig. 7). All collected individuals shared the same breeding sites. Live adult females were caught with nets. Mosquitoes were anesthetized with ether and the morphology of females was immediately observed in the field under a binocular microscope. Each mosquito individual was identified using morphological identification keys for *Ae. albopictus* [2–4, 9] as well as recommendations of the Walter Reed institute (Walter Reed Biosystematic Unit). Only females that could be seen to contain no blood upon microscopic observation of the gut contents were retained for analysis. Mosquitoes were stored in 100% ethanol at -80 °C until used.

### Morphological identification

Photographs of the scutellum and the head were made from 3 individuals of each of the two sub-species according to their genetic structure using nuclear and mitochondrial markers. The mouthparts of specimens were also analysed under a scanning electron microscope (SEM). The preparation of the mosquito’s cibarial armature followed an adopted method given by Sirivanakarn [41]. The head of each female was cut off with a sharp razor blade and macerated with 10% KOH at 95 °C until clearance. After clearance, heads were washed in a sodium chloride solution and then dissected with dissecting needles under a binocular microscope in a drop of the sodium chloride solution. Thereafter, the compound eyes were pulled apart to uncover the cibarium with the cibarial armature. For SEM, the samples were subsequently mounted on stubs using a drop of ethanol and then sputter-coated with gold. The samples were analyzed using a Hitachi S-45000 SEM scanning electron microscope. All photographs were compared to *Ae. albopictus* individuals from sampling sites of Vietnam and France.

### Amplification and analysis of mitochondrial markers

Mosquito DNA extraction was performed following our previous optimized protocol for individual whole mosquitoes [21]. A total of 43 individuals were analyzed (Tables 1 and 2). A 597-bp fragment flanking the mitochondrial cytochrome *c* oxidase subunit 1 (*cox*1) gene and a 450-bp fragment flanking the mitochondrial nicotine adenine dinucleotide dehydrogenase subunit 5 (*nad*5) gene were amplified by polymerase chain reaction (PCR) using 45 ng of DNA matrix as previously described [42]. PCR products were visualised on 1% agarose gels stained with ethidium bromide. Amplicons were sent for sequencing after purification procedure at a commercial laboratory (Biofidal, Lyon, France). The ITS1-5.8S-ITS2 region was amplified using the universal primers 18SFIN (5′-GTA AGC TTC CTT TGT ACA CAC CGC CCG T-3′) and CP16 (5′-GCG GGT ACC ATG CTT AAA TTT AGG GGG TA-3′) complementary to highly conserved sequences in the 18S and 28S rRNA [43]. Reactions were performed with 15 ng DNA, 1.5 mM MgCl_2_, 1× Reaction buffer, 0.1 mM dNTP, 0.24 μM of each primer and 1.25 U Taq DNA polymerase (Invitrogen, Cergy Pontoise, France). Amplifications were performed using following cycle conditions: an initial denaturing step at 97 °C for 4 min; 30 cycles of denaturation at 96 °C for 30 s, annealing at 48 °C for 30 s, extension at 72 °C for 2 min, followed by a final extension at 72 °C for 4 min. Amplification products were ligated into the TOPO 2.1 vector and transformed into competent One Shot cells using the TOPO TA cloning kit (Invitrogen). DNA inserts were sequenced on both strands. The two strands were aligned and sequencing errors were manually corrected. The sequences were then aligned using MUSCLE [44] algorithm through SEAVIEW software [45] and polymorphic haplotypes were determined using DnaSP v5.10 [46]. The haplotype (Hd) and nucleotidic (π) diversities were estimated with DnaSP v5.10. Each haplotype was submitted to GenBank under accession numbers KX495902–KX495951, KX573910–KX573911.

### Phylogenetic analysis

Sequences of the *cox*1 and *nad*5 genes and ITS2 region were used to study the evolutionary relationships between the individuals sampled in the forest of the National Park at Bù Gia Mập. Sequences from different species of the family *Culicidae* were selected in the GenBank database and used to build the phylogenetic trees. The *nad*5 phylogenetic tree was built using sequences of *Ae. albopictus* (gi|374252068), *Ae. notoscriptus* (gi|508599694, gi|508599680, gi|508599936, gi|508599982, gi|508599786, gi|508599774, gi|508599736, gi|508599844, gi|508599696), *Ae. vexans* (gi|291193228, gi|854892238, gi|291193230, gi|291193094, gi|291192962), *Ae. aegypti* (gi|66351510, gi|66351520, gi|66351512, gi|66351506, gi|164370713), *Culex pipiens* (gi|336462537, gi|336462529, gi|336462535, gi|336462539), *Anopheles gambiae* (gi|14699845, gi|459680999, gi|90656784), *An. albitarsis* (gi|309399294), *An. deaneorum* (gi|309399336), *An. oryzalimnetes* (gi|309399308), *An. arabiensis* (gi|751761052), *An. minimus* (gi|440655022, gi|440655020), *An. punctulatus* (gi|398314417), *An. hinesorum* (gi|398314375), *An. darling* (gi|260765279), *An. funestus* (gi|89112084). The *cox*1 phylogenetic tree was built using sequences of *Ae. flavopictus* (gi|966028896, gi|966028894, gi|966028892, gi|474421143, gi|474421139, gi|474420887, gi|474420611, gi|474421296, gi|474420608, gi|474420584), *Ae. lineatopennis* (gi|474420638, gi|474420641, gi|966028902), *Ae. cretinus* (gi|444435802), *Ae. mcintoshi* (gi|685228218, gi|685228170, gi|685228140, gi|685228156, gi|685228154), *Ae. daiten* (gi|474420480), *Ae. riversi* (gi|474421058, gi|474420596, gi|474420581), *Ae. cogilli* (gi|549534217), *Ae. scutellaris* (gi|806476819, gi|806476815), *Ae. togoi* (gi|844621172), *Ae. luniline* (gi|671184504), *Ae. vexans* (gi|951294276), *Ae. lilii* (gi|961557185), *Ae. cinereus* (gi|953287741, gi|674842465, gi|674842463), *Ae. aegypti* (gi|806458995, gi|806458969, gi|806458897, gi|347515179), *Cx. mimeticus* (gi|474420948), *Cx. sitiens* (gi|474420853), *Cx. erythrothorax* (gi|861455290), *Cx. inflantulus* (gi|474420520), *Cx. orientalis* (gi|966028824), *Cx. declarator* (gi|861455288), *Cx. pipiens* (gi|674842155, gi|585421109), *An. tesselatus* (gi|474420649), *An. aquasalis* (gi|440494782), *An. beklemishevi* (gi|374843442, gi|374843444, gi|374843430), *An. gambiae* (gi|292397399, gi|111183087). The ITS1-5,8S-ITS2 phylogenetic tree was built using sequences of *Ae. simpsoni* (gi|16930736, gi|16930732, gi|5566128, gi|1693072), *Ae. aegypti* (gi|159568), *Ae. ochraceus* (gi|685228632, gi|685228628), *Ae. mcintoshi* (gi|685228596), *Cx. quinquefasciatus* (gi|306992166), *Cx. pipiens* (gi|722301, gi|722294), *An. clowi* (gi|117936018, gi|117936017), *An. farauti* (gi|117935967), *An. annulipes* (gi|117936015, gi|117936014, gi|117936013). The likelihood of evolutionary model was estimated with jModelTest2 [47] using the Akaike Information Criterion corrected (AICc) and the Bayesian Information Criterion (BIC). Phylogenetic analyses were conducted with MrBayes v.3.2 [48]. Three independent Montecarlo Markov chains (MCMC) were run with 5,000,000 generations. A total of 1,750,000 trees were removed (‘burn-in’ step) and 3,250,000 trees per replicate were used to estimate the posterior probabilities and the consensus tree. The Potential Scale Reduction Factor confirmed the convergence of the chains.

### Detection of *Wolbachia* in whole individuals

*Wolbachia* infection status was evaluated in whole bodies of 43 mosquitoes by PCR detection of *wsp* gene (Tables 1 and 2). The PCR reaction mixture were performed in reaction volume of 25 μl which contained 1× PCR buffer, 1.5 mM of MgCl_2_, 0.2 μM of each primer, 40 μM of dNTP, 4 ng/μl of Bovin Serum Albumin (New England Biolabs, Ipswich, USA), 0.02 U/μl of Taq polymerase (Life Technologies, Saint-Aubin, France). The two generalist *wsp* primers 81 F (5′-TGG TCC AAT AAG TGA TGA AGA AAC-3′) and 691R (5′-AAA AAT TAA ACG CTA CTC CA-3′) were used [49]. The amplification protocol was held at 95 °C for 3 min, followed by 35 amplification cycles at 94 °C for 45 s, 52 °C for 40 s, 72 °C for 1 min and a final extension of 72 °C for 10 min. Amplified fragments of 611 bp were revealed under UV light after migration on 1% agarose gel electrophoresis. A positive control (DNA from C6/36 cell lines infected with *Wolbachia w*AlbB) and a negative control (DNA from uninfected C6/36 cell lines) were used.

### Analysis of midgut bacterial microbiota by b-ARISA

An additional set of 24 individuals were surface disinfected with 70% ethanol and rinsed with sterile water as described (Table 1). All dissection steps were performed under a sterile laminar flow hood in a containment environment and midgut DNA extracted as described [22]. Bacterial *Automatic Ribosomal Intergenic Spacer Analysis* (b-ARISA) was performed following the method of Cardinale et al. [50] with modifications. Reaction mixtures contained 1× of Q5 buffer (New England Biolabs, Ipswich, USA), 1× of HighGC buffer (New England Biolabs, Ipswich, USA), 0.5 μM of each primers (Invitrogen), 200 μM of dNTP (Applied Biosystem, Waltham, USA), 120 μg/ml of Bovine Serum Albumin (New England Biolabs, Ipswich, USA), 0.06 mg/ml of T4 gene 32 and 0.7 U of Q5 DNA polymerase (New England Biolabs, Ipswich, USA) and 30 ng of template DNA in a final volume of 25 μl. The primers were ITSF (5′-FAM-GTC GTA ACA AGG TAG CCG TA-3′) and ITSReub (5′-GCC AAG GCA TCC ACC-3′) and primer ITSF was 5′ end-labelled with the phosphoramidite dye 6-FAM. Reaction mixtures were held at 94 °C for 3 min, followed by 35 cycles of amplification at 94 °C for 45 s, 55 °C for 1 min, and 72 °C for 1 min 20 s and a final extension of 72 °C for 1 min 20 s. Three replicates per sample were necessary to obtain a sufficient amount of matrix to analyse. Bacterial DNA (*Micrococcus* sp.) and water were used as positive and negative controls, respectively. The PCR products were purified with QIAquick PCR Purification Kit (Qiagen, Hilden, Germany) and 80 ng of PCR product was loaded with 3 μl of Hi-Di-Formamide and 1 μl of GS-1200 LIZ internal size standard. Sizing tables were obtained with Genemapper 4.0 (Life technologies, Saint-Aubin, France) and imported in R software (R development core team). Only signals within the range of 100–1000 bp were considered for this study. Pick areas were transformed to Relative Fluorescence Intensity (Pick Area/Σ_Pick Area_) with the interactive binner and an optimal window size of 5 bp with a shift of 1 bp was selected according to the method previously described [51] (Additional file 2: Table S3). α and β-diversity analysis were performed with *vegan* package. DNA extraction was also performed from the remaining carcasses of each individual and ITS1-5.8S-ITS2 region was amplified as described above. Samples were then assigned to a clade according to the size of amplified fragments after migration of PCR products on a 1% agarose gel electrophoresis.

### MiSeq 16S rDNA gene sequencing

To determine the composition of midgut associated bacterial microbiota and their diversity, we also used the Illumina MiSeq sequencing technique of the 16S V5-V6 rDNA gene region as previously described [22]. The three *Ae. albopictus* individuals are part of a previous study performed with the same set of samples and their raw data reads are accessible from the European Nucleotide Archive (www.ebi.ac.uk/ena) under the accession number PRJEB6896 [22]. Two individuals corresponding to the cryptic species were included in this analysis (Table 1). Briefly, midgut DNA was extracted as previously described [22]. Libraries were built with one step of PCR conducted with 784 F (5′-AGG ATT AGA TAC CCT GGT A-3′) and 1061R (5′-CRR CAC GAG CTG ACG AC-3′) modified with an 8-bp multiplex barcode and Illumina adapters. The PCR mixture contained 1.75 U of Expand High Fidelity Enzyme Mix (Roche, Basel, Switzerland), 1× of Expand High Fidelity Buffer (Roche, Switzerland), 0.06 mg.ml^-1^ of T4 gene 32 protein (New England Biolabs, France), 40 μM of dNTP mix, 200 nM of each primers (Life Technologies, France). Amplifications were conducted with 5 min at 95 °C followed by 40 cycles at 95 °C for 40 s, 54.2 °C for 1 min, 72 °C for 30 s and a final extension at 72 °C for 7 min. Three PCR per sample were pooled together and equimolar product were mixed for all the samples libraries after purification with the Agencourt AMPure XP PCR Purification kit (Beckman Coulter, Villepinte, France). Sequencing was performed on the Illumina MiSeq platform (2 × 250 bp paired-end reads) by ProfileXpert - ViroScan 3D (Lyon, France). The complementary reads were aligned with PandaSeq [52]. Aligned sequences were then processed with the MOTHUR pipeline and were kept only if: (i) their size was comprised between 200 and 350 bp; (ii) they did not contain any ambiguous sequence; (iii) they were not chimeric according to Perseus chimera detection; (iv) they aligned on the SILVA database (released 115). OTUs were created by clustering sequences at a level of 97% similarity according to the median neighbour method [53]. The sequences were classified with a naïve Bayesian classifier at an 80% bootstrap [54]. A total of 51843, 81088, 32279, 69453 and 34077 sequences were obtained for the samples BGM1, BGM3, BGM4, BGM5 and BGM6, respectively. A negative control (blank extraction and PCR) was used as a reference to remove the contaminants as previously described [22]. The sequence proportions were obtained after subsampling of 8002 sequences per sample. Because of the small samples size, no comparative analysis of the α and β diversity was performed. Only the proportions of OTUs assignment were presented. The raw reads related to the two individuals from the clade C have been deposited in the European Nucleotide Archive (www.ebi.ac.uk/ena) under the accession number PRJEB14610. DNA extraction, *cox*1 and *nad*5 barcoding were performed on the carcasses of those individuals according to the protocol previously described.

## Additional files

Additional file 1: Figure S1. Correlation of pairwise nucleotidic distances for *cox*1, *nad*5 and ITS1-5.8S-ITS2 markers. **Figure S2.** Molecular features reveal differences between *Ae. albopictus* and a cryptic *Aedes* species living in sympatry. **Table S1.** Analysis of haplotype and nucleotide diversity within the two *Aedes* clades. **Table S2.** Proportions of operational taxonomic units (OTU) of bacteria identified in midgut samples by 16S rDNA Miseq sequencing. (PDF 1193 kb)

Additional file 2: Table S3. b-ARISA dataset. (XLSX 25 kb)

## Abbreviations

16S rDNA: 16S ribosomal DNA
CHICKV: Chickungunya virus
*cox*1: Cytochrome *c* oxidase subunit 1
DENV: Dengue virus
Hd: Haplotype diversity
ITS: Internal transcribed spacer
K2P: Kimura 2-parameter
*nad*5: Nicotinamide adenine dinucleotide dehydrogenase subunit 5
