# Supplementary material for: Identification of sympatric cryptic species of Aedes albopictus subgroup in Vietnam: new perspectives in phylosymbiosis of insect vector
Source: Parasit Vectors. 2017 Jun 2;10:276. doi: 10.1186/s13071-017-2202-9 (PMC5457575; doi:10.1186/s13071-017-2202-9)
Supplement: Supplementary file 1 — Correlation of pairwise nucleotidic distances for cox1, nad5 and ITS1-5.8S-ITS2 markers. Figure S2. Molecular features reveal differences between Ae. albopictus and a cryptic Aedes species living in sympatry. Table S1. Analysis of haplotype and nucleotide diversity within the two Aedes clades. Table S2. Proportions of operational taxonomic units (OTU) of bacteria identified in midgut samples by 16S rDNA Miseq sequencing. (PDF 1193 kb) [file 13071_2017_2202_MOESM1_ESM.pdf]

Supplementary informations:

Identification of sympatric cryptic species of *Aedes albopictus* subgroup in Vietnam: new perspectives in phylosymbiosis of insect vector

Extracted from Genebank the 5th of May 2016 :

>gi|340050923|gb|HQ398902.1| Aedes albopictus voucher VN103-9 cytochrome oxidase subunit I (COI) gene, partial cds; mitochondrial
AACATTATATTTTATTTTTGGAGTTTGATCGGGAATAGTAGGAACTTCATTAAGAATTTTAATTCGTACAGA
ACTTAGCCACCCAGGAATATTTATTGGAAATGATCAAATTTATAATGTAATTGTTACAGCTCATGCATTTA
TTATAATTTTTTTTATAGTTATACCAATTATAATTGGAGGATTTGGAAATTGATTAGTACCTTTAATATTAG
GAGCCCCTGATATAGCTTTTCCTCGAATAAATAATATAAGTTTCTGAATACTTCCTCCTTCTTTAACACTT
CTTCTTTCTAGTTCTATAGTAGAAAATGGAGCTGGAACTGGATGAACTGTTTACCCTCCTCTTTCTTCTG
GAACTGCTCATGCCGGGGCTTCAGTTGATTTAGCAATTTTTTCTTTACATTTAGCAGGAATTTCTTCAATT
TTAGGAGCAGTAAATTTTATTACGACTGTAATTAATATACGATCAGCTGGAATTACTCTTGATCGTCTTCC
ATTATTTGTATGATCTGTTGTTATTACAGCTATTTTATTACTTCTATCTTTACCTGTTTTAGCGGGAGCTAT
TACTATATTATTAACTGACCGAAATTTAAATACTTCTTTTTTTGATCCAATTGGAGGAGGAGACCCTATTC
TTTACCAACACTTATTT

>gi|745794145|gb|KM502236.1| Aedes albopictus voucher whlpx001 cytochrome c oxidase subunit I (COI) gene, partial cds; mitochondrial
TTTATTTTTGGAGTTTGATCGGGAATAGTAGGAACTTCATTAAGAATTTTAATTCGTACAGAACTTAGCCA
CCCAGGAATATTTATTGGAAATGATCAAATTTATAATGTAATTGTTACAGCTCATGCATTTATTATAATTTT
TTTTATAGTTATACCAATTATAATTGGAGGATTTGGAAATTGATTAGTACCTTTAATATTAGGAGCCCCTG
ATATAGCTTTTCCTCGAATAAATAATATAAGTTTCTGAATACTTCCTCCTTCTTTAACACTTCTTCTTTCTA
GTTCTATAGTAGAAAATGGAGCTGGAACTGGATGAACTGTTTACCCTCCTCTTTCTTCTGGAACTGCTCA
TGCCGGGGCTTCAGTTGATTTAGCAATTTTTTCTTTACATTTAGCAGGAATTTCTTCAATTTTAGGAGCAG
TAAATTTTATTACGACTGTAATTAATATACGATCAGCTGGAATTACTCTTGATCGTCTTCCATTATTTGTAT
GATCTGTTGTTATTACAGCTATTTTATTACTTCTATCTTTACCTGTTTTAGCGGGAGCTATTACTATATTAT
TAACTGACCGAAATTTAAATACTTCTTTTTTTGATCCAAT

Both sequences had a 99 % identity score with the Cryptic *Aedes* sp. described in the current publication.

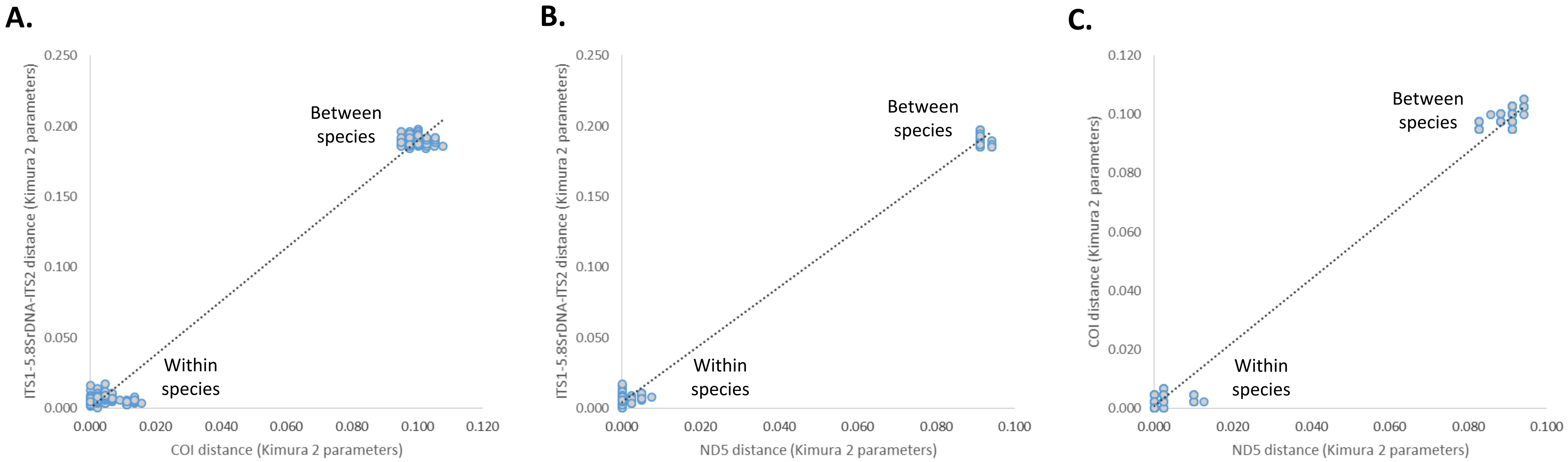

Figure S1. Correlation of pairwise nucleotidic distances among COI, ND5 and ITS1-5.8SrDNA-ITS2 markers. The Kimura 2 parameters based distances have been estimated among each pair of samples and plotted for the same samples genotyped with (A) ITS1-5.8SrDNA-ITS2 and COI, (B) ITS1-5.8SrDNA-ITS2 and ND5, (C) COI and ND5. Distances within and among *Aedes albopictus* and the Cryptic *Aedes* sp. species are highlighted.

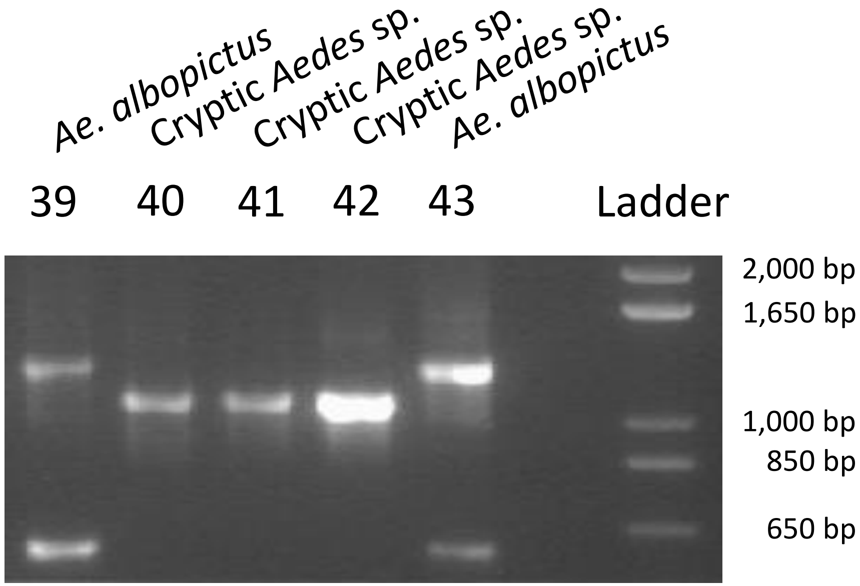

Figure S2. Molecular features reveal differences between *Ae. albopictus* and a Cryptic *Aedes* species living in sympatry. ITS1-5.8SrDNA-ITS2 amplicons differs in size between individuals belonginig to *Ae. albopictus* (39 and 43) and the cryptic *Aedes* sp. (40,41 and 42). An additional amplicon (< 560bp) of the same region from the symbiotic protist *Ascogregarina taiwanensis* might be visible for *Ae. albopictus* individuals.

Table S1. Analysis of haplotypes and nucleotides diversity within the two *Aedes* groups.

**Marker**	**Group**	**$n_{individuals}$**	**$n_{haplotypes}$**	**S***	**Hd****	**π*****
**COI**	A	20	9	9	0.70	0.0018
	C	28	12	16	0.74	0.0028
**ND5**	A	19	3	5	0.20	0.0012
	C	25	4	4	0.23	0.0007
**ITS1-5.8SrDNA-ITS2**	A	5	5	1085	1	0.68
	C	20	20	1141	1	0.63

*Number of segregating sites

** Haplotype diversity

***Nucleotide diversity

TableS2. Proportions of Operational Taxonomic Units (OTU) identified in midgut samles

Taxonomy (Bootstrap)	OTU	BGM1	BGM3	BGM4	BGM5	BGM6
Dysgonomonas(100)	Otu000001	0.0227325807707279	0.648712821794551	0.401287085514834	0.601298214954438	0.402693454023623
Asaia(91)	Otu000003	0	0	0.000218150087260035	0	0.00231813665967546
Aeromonas(100)	Otu000005	0	0	0	0.0515541130944951	0
Novosphingobium(97)	Otu000012	0.241027637212923	0.018495376155961	0.0591186736474695	0.0195980526775683	0.0260514405563528
Aquabacterium(90)	Otu000013	0.002958349552355	0.00174956260934766	0.00512652705061082	0.00074897016602172	0.00452588585936638
Caulobacter(67)	Otu000014	0.227092253795251	0.0212446888277931	0.0407940663176265	0.0103607539633005	0.0152334694778673
Dysgonomonas(100)	Otu000017	0.000155702608018684	0.00487378155461135	0.00218150087260035	0.00237173885906878	0.00430511093939728
Methylobacterium(100)	Otu000021	0.0741144414168937	0	0.0349040139616056	0	0
Acinetobacter(100)	Otu000023	0	0.00362409397650587	0	0	0.000110387459984546
Hydrotalea(100)	Otu000024	0.0653172440638381	0.00424893776555861	0.00992582897033159	0.0037448508301086	0.00684402251904184
Asaia(83)	Otu000030	0	0	0	0	0.000110387459984546
unclassified(100)	Otu000033	0	0	0.000109075043630017	0	0
unclassified(63)	Otu000034	0.0180615025301674	0	0.00861692844677138	0	0
Sphingomonas(100)	Otu000039	0.0434410276372129	0.00787303174206448	0.0282504363001745	0.0104855823243041	0.00872060933877911
Dysgonomonas(100)	Otu000041	0	0.000624843789052737	0.000545375218150087	0.00049931344401448	0.000331162379953637
Pseudomonas(97)	Otu000043	0	0.000874781304673832	0	0.00024965672200724	0.000551937299922729
Sphingobium(94)	Otu000044	0.0319968859478396	0.00374906273431642	0.0280322862129145	0.0104855823243041	0.0139088199580528
Enterococcus(90)	Otu000045	0	0.000749812546863284	0.000218150087260035	0.00012482836100362	0
Leuconostoc(100)	Otu000046	0.000155702608018684	0	0.0018542757417103	0.00536761952315566	0
Dysgonomonas(100)	Otu000047	0	0.000749812546863284	0.00043630017452007	0.00074897016602172	0.000551937299922729
Dysgonomonas(100)	Otu000048	0.000155702608018684	0.00149962509372657	0.000545375218150087	0.00024965672200724	0.000441549839938183
unclassified(53)	Otu000050	0.0190735694822888	0.0108722819295176	0.0566099476439791	0	0.0466938955734629
Ralstonia(75)	Otu000051	0.000934215648112106	0.000124968757810547	0.000763525305410122	0.00112345524903258	0.000441549839938183
Aeromonas(100)	Otu000054	0	0.000249937515621095	0.000327225130890052	0.00024965672200724	0.000331162379953637
Brevundimonas(96)	Otu000055	0.00264694433631763	0.00287428142964259	0.00261780104712042	0.0087379852702534	0.00353239871950546
Ewingella(63)	Otu000056	0	0.00399900024993752	0.00479930191972077	0.00262139558107602	0.00353239871950546
unclassified(100)	Otu000060	0.000467107824056053	0.000249937515621095	0.000109075043630017	0.00037448508301086	0.000110387459984546
Asaia(72)	Otu000061	0	0	0.000109075043630017	0	0
Novosphingobium(100)	Otu000062	0.00186843129622421	0.000249937515621095	0.000654450261780105	0.00049931344401448	0.000110387459984546
Methylobacterium(100)	Otu000063	0.0242117555469054	0	0.0145069808027923	0	0
unclassified(85)	Otu000065	0	0.0044988752811797	0.00327225130890052	0.00411933591311946	0.00121426205983

unclassified(100)	Otu000066	0.000544959128065395	0	0.000109075043630017	0	0
Pseudomonas(89)	Otu000067	0	0	0	0.00012482836100362	0.000110387459984546
Cellvibrio(94)	Otu000068	0	0.00399900024993752	0	0.0106104106853077	0
Novosphingobium(100)	Otu000069	0.00108991825613079	0	0.000654450261780105	0.00012482836100362	0.000220774919969092
Aeromonas(100)	Otu000070	0.0000778513040093422	0	0.000109075043630017	0.00012482836100362	0.000220774919969092
unclassified(99)	Otu000073	0.000934215648112106	0	0	0.00224691049806516	0
Gluconobacter(99)	Otu000076	0	0	0	0	0.151451595098797
unclassified(86)	Otu000077	0.000934215648112106	0	0.000545375218150087	0	0
Novosphingobium(89)	Otu000079	0.00124562086414947	0.000124968757810547	0.000327225130890052	0.00012482836100362	0.000220774919969092
unclassified(98)	Otu000080	0	0.000249937515621095	0.000218150087260035	0.00024965672200724	0.000110387459984546
Streptococcus(100)	Otu000083	0	0	0.0025087260034904	0.00287105230308326	0.00220774919969092
Novosphingobium(92)	Otu000085	0.00132347216815882	0.000124968757810547	0.000327225130890052	0	0.000220774919969092
Rhizobacter(90)	Otu000086	0	0	0.000981675392670157	0	0.00132464951981455
unclassified(95)	Otu000093	0	0	0.00087260034904014	0.00012482836100362	0.000551937299922729
Rhizobium(100)	Otu000094	0.0128454651615415	0.00487378155461135	0.00610820244328098	0.00099862688802896	0.00640247267910365
Lacibacter(100)	Otu000097	0	0	0.0471204188481675	0	0.0205320675571255
Tepidimonas(98)	Otu000098	0	0	0	0.00012482836100362	0.000110387459984546
Aquabacterium(64)	Otu000099	0.000155702608018684	0	0.000327225130890052	0	0.000220774919969092
unclassified(86)	Otu000102	0.0000778513040093422	0	0.000545375218150087	0.00574210460616652	0.000110387459984546
Chryseobacterium(100)	Otu000103	0	0	0.000109075043630017	0	0
Streptococcus(100)	Otu000104	0	0.00149962509372657	0.000109075043630017	0.00424416427412308	0
Sphingobium(100)	Otu000105	0.000389256520046711	0.000124968757810547	0.000327225130890052	0.00049931344401448	0.000110387459984546
Afipia(99)	Otu000109	0.00957571039314909	0.00374906273431642	0.00469022687609075	0	0.00154542443978364
Caedibacter(100)	Otu000111	0.0137018295056442	0.000124968757810547	0.00294502617801047	0	0.000662324759907275
unclassified(77)	Otu000112	0.000233553912028026	0	0.00043630017452007	0.00024965672200724	0.000551937299922729
unclassified(98)	Otu000113	0	0.00137465633591602	0.000109075043630017	0.00024965672200724	0
Enhydrobacter(100)	Otu000117	0	0.00149962509372657	0	0	0
Hydrotalea(100)	Otu000118	0.000544959128065395	0	0.000109075043630017	0	0
Cloacibacterium(85)	Otu000119	0.0000778513040093422	0.00262434391402149	0.00152705061082024	0	0.000993487139860912
unclassified(100)	Otu000120	0	0	0	0.00024965672200724	0.000110387459984546
Streptococcus(100)	Otu000121	0.0000778513040093422	0	0.00087260034904014	0.00449382099613032	0
unclassified(100)	Otu000123	0	0.000624843789052737	0	0.0074897016602172	0
Pseudomonas(94)	Otu000125	0	0.00512371907023244	0.00272687609075044	0.00386967919111222	0.00485704823932001

unclassified(55)	Otu000132	0.0000778513040093422	0.000374906273431642	0.000109075043630017	0.00024965672200724	0
Aquabacterium(93)	Otu000133	0.0000778513040093422	0	0.000654450261780105	0	0.000331162379953637
Staphylococcus(100)	Otu000134	0	0.000249937515621095	0	0	0.000110387459984546
unclassified	Otu000135	0.000233553912028026	0.000499875031242189	0.00109075043630017	0.0006241418050181	0
Dysgonomonas(100)	Otu000136	0.0000778513040093422	0.000624843789052737	0.00043630017452007	0.00024965672200724	0.00242852411966001
Dysgonomonas(100)	Otu000137	0	0	0.000109075043630017	0.00012482836100362	0
Aquabacterium(77)	Otu000139	0.000155702608018684	0	0.000545375218150087	0.00012482836100362	0.000110387459984546
unclassified(97)	Otu000140	0	0.00174956260934766	0.000654450261780105	0.00112345524903258	0.000993487139860912
Chryseobacterium(100)	Otu000141	0	0.00349912521869533	0.00174520069808028	0.00037448508301086	0.000331162379953637
Dysgonomonas(100)	Otu000143	0	0.000249937515621095	0.000109075043630017	0.00012482836100362	0
Dysgonomonas(100)	Otu000144	0.000155702608018684	0.0023744063984004	0.000327225130890052	0.00349519410810136	0.00077271221989182
Novosphingobium(84)	Otu000145	0.000934215648112106	0	0.000218150087260035	0	0.000220774919969092
unclassified(99)	Otu000146	0	0.000124968757810547	0	0	0.000110387459984546
Finegoldia(100)	Otu000148	0	0.00362409397650587	0	0	0
unclassified(58)	Otu000150	0.000467107824056053	0.000249937515621095	0.000218150087260035	0.00024965672200724	0.000441549839938183
Bacillus(100)	Otu000151	0	0	0	0.00024965672200724	0
Flavobacterium(100)	Otu000153	0	0.000124968757810547	0.000109075043630017	0	0
Caulobacter(79)	Otu000154	0.000856364344102764	0	0	0.00012482836100362	0
unclassified(88)	Otu000156	0.00179057999221487	0	0.000327225130890052	0.00024965672200724	0.000110387459984546
Myroides(99)	Otu000157	0	0.00124968757810547	0.000763525305410122	0.00224691049806516	0
Chryseobacterium(99)	Otu000160	0	0.000124968757810547	0	0	0.000220774919969092
unclassified(100)	Otu000161	0	0	0.000654450261780105	0	0.000220774919969092
Paracoccus(100)	Otu000163	0.000389256520046711	0.00512371907023244	0.00141797556719023	0.0107352390463113	0.00430511093939728
unclassified(73)	Otu000164	0	0.000124968757810547	0	0.00012482836100362	0
unclassified(100)	Otu000165	0.000155702608018684	0	0	0	0
Dysgonomonas(100)	Otu000166	0	0	0.000109075043630017	0.00012482836100362	0.000331162379953637
Chryseobacterium(100)	Otu000167	0	0.00112471882029493	0	0.00074897016602172	0
unclassified(100)	Otu000168	0.000311405216037369	0	0	0	0
Staphylococcus(100)	Otu000169	0	0.00312421894526368	0	0.0024965672200724	0
Lacibacter(100)	Otu000170	0	0.000124968757810547	0.000545375218150087	0	0.000220774919969092
unclassified(98)	Otu000171	0	0.000124968757810547	0	0.00012482836100362	0
unclassified(63)	Otu000172	0.0000778513040093422	0	0.000218150087260035	0	0.000110387459984546
Chryseobacterium(86)	Otu000174	0	0	0.00163612565445026	0	0

Staphylococcus(100)	Otu000175	0	0.000124968757810547	0	0.00012482836100362	0
Streptococcus(100)	Otu000176	0	0	0	0	0.000110387459984546
Rhizobiales_bacterium_NHI-8(64)	Otu000177	0.00217983651226158	0.00299925018745314	0.00567190226876091	0	0.00463627331935092
unclassified(100)	Otu000180	0	0.000124968757810547	0	0	0
unclassified(100)	Otu000181	0	0.00574856285928518	0.0232329842931937	0.00049931344401448	0.0120322331383155
Pseudomonas(100)	Otu000185	0	0.000249937515621095	0	0	0
Lacibacter(100)	Otu000187	0	0.000249937515621095	0.000654450261780105	0	0.000441549839938183
Methylobacterium(100)	Otu000189	0.00210198520825224	0	0	0.00049931344401448	0.00342201125952092
unclassified(100)	Otu000190	0	0.000249937515621095	0.000109075043630017	0	0
Chryseobacterium(100)	Otu000193	0.0000778513040093422	0.000374906273431642	0.000109075043630017	0	0
Pantoea(55)	Otu000195	0	0	0	0.00049931344401448	0
Bacillus(100)	Otu000197	0	0	0	0.00012482836100362	0
Aquabacterium(74)	Otu000200	0	0	0.000218150087260035	0	0.000110387459984546
Dysgonomonas(100)	Otu000201	0	0	0	0	0.000220774919969092
unclassified(100)	Otu000202	0	0	0.000109075043630017	0	0
unclassified(53)	Otu000203	0.00264694433631763	0.000624843789052737	0.00207242582897033	0.00037448508301086	0.00176619935975273
unclassified(60)	Otu000205	0.0014791747761775	0	0	0.0006241418050181	0
Anaerococcus(100)	Otu000208	0.000389256520046711	0.000499875031242189	0	0.00948695543627512	0
Chryseobacterium(100)	Otu000209	0	0	0.000109075043630017	0	0
unclassified(99)	Otu000210	0.0000778513040093422	0	0	0	0
Anaerococcus(100)	Otu000212	0	0	0	0.00049931344401448	0
unclassified(69)	Otu000213	0.0000778513040093422	0	0	0	0
Gluconobacter(93)	Otu000215	0	0	0	0	0.0259410530963683
Sphingobium(98)	Otu000221	0.000622810432074737	0	0.000218150087260035	0.00024965672200724	0.000110387459984546
unclassified(100)	Otu000222	0	0	0	0.00012482836100362	0.000110387459984546
Haemophilus(100)	Otu000223	0.000155702608018684	0.000499875031242189	0.00087260034904014	0.0012482836100362	0.00077271221989182
unclassified(100)	Otu000224	0	0	0.000109075043630017	0	0
unclassified(100)	Otu000225	0	0	0	0.00137311197103982	0
Dysgonomonas(100)	Otu000226	0	0	0.000109075043630017	0	0
Escherichia-Shigella(83)	Otu000227	0.0000778513040093422	0	0	0	0
unclassified(89)	Otu000228	0.000155702608018684	0	0	0	0.000331162379953637
Dysgonomonas(100)	Otu000230	0	0	0	0	0.000110387459984546
Flectobacillus(100)	Otu000232	0.0000778513040093422	0	0.00119982547993019	0.00087379852702534	0.00110387459984546

Flavobacterium(100)	Otu000233	0	0	0	0.00024965672200724	0
unclassified(100)	Otu000236	0.000155702608018684	0	0	0.00012482836100362	0.000110387459984546
Sphingobium(98)	Otu000241	0.000155702608018684	0	0.000218150087260035	0	0.000110387459984546
Sphingopyxis(88)	Otu000242	0	0	0.000109075043630017	0	0
unclassified(100)	Otu000245	0	0.000124968757810547	0	0	0.000110387459984546
unclassified(100)	Otu000246	0.0424289606850915	0.00374906273431642	0.00818062827225131	0.0024965672200724	0.000993487139860912
Aquabacterium(83)	Otu000247	0.000155702608018684	0	0.000218150087260035	0	0.000110387459984546
Spirosoma(100)	Otu000248	0.0219540677306345	0	0.000763525305410122	0.00037448508301086	0
Sphingomonas(100)	Otu000249	0	0.000374906273431642	0	0	0
unclassified(100)	Otu000251	0.000233553912028026	0	0.000218150087260035	0	0.000220774919969092
unclassified(95)	Otu000252	0.0000778513040093422	0	0.000654450261780105	0	0.000110387459984546
Pelomonas(77)	Otu000253	0.0000778513040093422	0	0.000109075043630017	0	0
Ralstonia(62)	Otu000257	0.000155702608018684	0.000124968757810547	0	0.00012482836100362	0.000110387459984546
unclassified(100)	Otu000258	0	0.000124968757810547	0	0	0
unclassified(100)	Otu000259	0	0	0	0.00012482836100362	0
Paracoccus(98)	Otu000261	0.000233553912028026	0	0	0.00137311197103982	0
Chryseobacterium(100)	Otu000262	0	0.000124968757810547	0	0.0012482836100362	0.000110387459984546
Flavobacterium(93)	Otu000263	0.000155702608018684	0.00324918770307423	0	0.0006241418050181	0
unclassified(95)	Otu000264	0	0.00112471882029493	0	0	0
Neochlamydia(100)	Otu000265	0.00428182172051382	0.00124968757810547	0.000109075043630017	0	0
Pseudochrobactrum(100)	Otu000266	0.000155702608018684	0.000499875031242189	0	0	0
Flexibacter(93)	Otu000268	0.000155702608018684	0.00162459385153712	0.00130890052356021	0	0.00474666077933547
Acidocella(100)	Otu000270	0.0000778513040093422	0	0	0.00037448508301086	0
Streptococcus(100)	Otu000272	0.0000778513040093422	0	0	0	0
Brevundimonas(100)	Otu000273	0.0000778513040093422	0.00137465633591602	0.00174520069808028	0	0
Alkanindiges(100)	Otu000274	0	0	0	0.00012482836100362	0
Dolosigranulum(100)	Otu000275	0	0.00137465633591602	0.000327225130890052	0	0
Legionella(100)	Otu000276	0.00101206695212145	0	0	0.00212208213706154	0.000551937299922729
unclassified(87)	Otu000277	0	0	0.000218150087260035	0	0
Reyranella(100)	Otu000278	0.000155702608018684	0.000249937515621095	0.00479930191972077	0	0.000441549839938183
Bosea(100)	Otu000279	0.000233553912028026	0.000874781304673832	0.000654450261780105	0.00037448508301086	0.00198697427972182
Dysgonomonas(84)	Otu000281	0	0	0.00305410122164049	0.00087379852702534	0.000551937299922729
Methylobacterium(100)	Otu000283	0.0000778513040093422	0	0	0	0.000331162379953637

Pseudochrobactrum(98)	Otu000284	0	0.000124968757810547	0.00152705061082024	0	0
Pseudomonas(100)	Otu000285	0	0	0	0.00012482836100362	0.000110387459984546
unclassified(75)	Otu000286	0	0	0.000109075043630017	0	0
unclassified(86)	Otu000291	0.0000778513040093422	0	0.000218150087260035	0	0
unclassified(98)	Otu000293	0	0	0.000545375218150087	0	0.000110387459984546
Chryseobacterium(100)	Otu000294	0	0.000249937515621095	0	0	0
unclassified(52)	Otu000296	0	0	0.000218150087260035	0.00012482836100362	0.000110387459984546
Aquabacterium(86)	Otu000298	0	0	0.000109075043630017	0.00012482836100362	0
Paracoccus(95)	Otu000300	0	0	0	0.00024965672200724	0
Aerococcus(100)	Otu000301	0.000155702608018684	0.00374906273431642	0	0.00149794033204344	0.00077271221989182
Hymenobacter(100)	Otu000302	0	0.0044988752811797	0	0.00349519410810136	0
Enterococcus(100)	Otu000303	0	0	0	0	0.000110387459984546
Aquabacterium(83)	Otu000304	0	0.000249937515621095	0.000218150087260035	0	0.000110387459984546
Hymenobacter(100)	Otu000305	0.000233553912028026	0.00299925018745314	0.00109075043630017	0.00711521657720634	0.00154542443978364
unclassified(100)	Otu000308	0	0	0	0.00012482836100362	0
Prevotella(100)	Otu000312	0	0	0	0	0.000110387459984546
Novosphingobium(100)	Otu000314	0.000233553912028026	0	0.000218150087260035	0	0
unclassified(100)	Otu000315	0	0	0	0.00012482836100362	0.000110387459984546
Chryseobacterium(100)	Otu000317	0	0.0021244688827793	0	0.00037448508301086	0
Bradyrhizobium(100)	Otu000318	0	0	0	0.00149794033204344	0.00209736173970637
Aquabacterium(85)	Otu000321	0.0000778513040093422	0	0	0	0
unclassified(100)	Otu000322	0	0	0.000218150087260035	0	0
Aquabacterium(91)	Otu000323	0	0	0.000109075043630017	0	0.000110387459984546
Rheinheimera(100)	Otu000327	0.000155702608018684	0	0.000327225130890052	0.00349519410810136	0
Legionella(97)	Otu000329	0	0.00324918770307423	0	0.00012482836100362	0.000441549839938183
Aquabacterium(91)	Otu000333	0	0	0.000218150087260035	0	0
Rubellimicrobium(100)	Otu000335	0	0	0	0.0012482836100362	0.0038635610994591
Paracoccus(100)	Otu000336	0	0	0	0.00137311197103982	0
Gemella(100)	Otu000337	0	0	0.000109075043630017	0.00012482836100362	0
Hymenobacter(100)	Otu000341	0	0	0	0	0.00231813665967546
Hyphomicrobium(100)	Otu000344	0	0.00199950012496876	0.00272687609075044	0	0
unclassified(87)	Otu000345	0.000155702608018684	0	0.000109075043630017	0	0.000110387459984546
unclassified(97)	Otu000348	0	0	0	0.00012482836100362	0

Sphingomonas(100)	Otu000349	0	0	0	0.00024965672200724	0
Pseudomonas(100)	Otu000352	0	0	0	0	0.000110387459984546
unclassified(59)	Otu000353	0	0.000374906273431642	0	0	0
unclassified(86)	Otu000358	0	0.00299925018745314	0	0	0
Pseudomonas(97)	Otu000361	0	0	0	0	0.000110387459984546
Pseudomonas(90)	Otu000362	0	0.000124968757810547	0	0	0
Chryseobacterium(83)	Otu000363	0	0	0	0	0.00143503697979909
Neisseria(97)	Otu000365	0	0	0.000109075043630017	0	0
Chryseobacterium(79)	Otu000367	0	0.000124968757810547	0	0	0
unclassified(100)	Otu000368	0	0	0.000109075043630017	0	0
Rhizobium(97)	Otu000370	0	0.000999750062484379	0	0	0
unclassified(100)	Otu000371	0	0	0	0.00012482836100362	0
unclassified(97)	Otu000376	0	0	0	0.00012482836100362	0
unclassified(100)	Otu000378	0	0	0	0.00012482836100362	0.000110387459984546
Novosphingobium(67)	Otu000379	0.00373686259244842	0	0.000327225130890052	0	0.000110387459984546
Sphingobium(100)	Otu000381	0	0	0	0.00037448508301086	0
Mesorhizobium(62)	Otu000382	0.000233553912028026	0	0.00087260034904014	0.0006241418050181	0
Cellvibrio(100)	Otu000387	0	0.000124968757810547	0	0	0
unclassified(66)	Otu000388	0	0	0	0.00012482836100362	0
Pedobacter(97)	Otu000390	0	0	0.0018542757417103	0	0
Aeromonas(100)	Otu000393	0	0.000124968757810547	0	0.00012482836100362	0
Sphingomonas(100)	Otu000394	0	0	0.000218150087260035	0	0
SM1A02(100)	Otu000395	0	0.000124968757810547	0.000218150087260035	0	0
Dysgonomonas(100)	Otu000396	0	0	0	0.00012482836100362	0
unclassified(76)	Otu000400	0	0.000624843789052737	0.000981675392670157	0	0.000331162379953637
Pseudomonas(100)	Otu000404	0	0	0	0	0.000110387459984546
unclassified(100)	Otu000406	0	0	0	0	0.000110387459984546
Novosphingobium(100)	Otu000407	0.000233553912028026	0	0	0	0
Methylobacterium(100)	Otu000410	0	0	0	0.00012482836100362	0
Streptococcus(100)	Otu000411	0	0	0	0.00037448508301086	0.000662324759907275
Rhizobium(100)	Otu000419	0	0	0	0.00012482836100362	0
Dysgonomonas(100)	Otu000421	0	0	0	0.00012482836100362	0.000110387459984546
Novosphingobium(88)	Otu000423	0.000233553912028026	0	0	0	0

unclassified(100)	Otu000426	0	0	0.000109075043630017	0	0
Aquabacterium(74)	Otu000429	0	0	0.000109075043630017	0	0
unclassified(66)	Otu000431	0.0000778513040093422	0	0.00523560209424084	0.00012482836100362	0.00231813665967546
Asticcacaulis(61)	Otu000432	0	0	0.00109075043630017	0.00087379852702534	0.000331162379953637
unclassified(100)	Otu000434	0.0000778513040093422	0	0	0	0
Sphingobium(73)	Otu000441	0.00490463215258856	0	0.00043630017452007	0	0
unclassified(73)	Otu000443	0.000544959128065395	0	0.000763525305410122	0.00024965672200724	0.000551937299922729
Acinetobacter(91)	Otu000444	0	0.000124968757810547	0.000327225130890052	0	0
Tepidimonas(78)	Otu000447	0	0	0	0.00012482836100362	0
Sphingopyxis(100)	Otu000448	0	0	0.000327225130890052	0	0
Sphingopyxis(96)	Otu000449	0.000311405216037369	0	0	0	0
Sphingomonas(100)	Otu000450	0.0000778513040093422	0	0	0	0
Eremococcus(100)	Otu000455	0	0	0.00163612565445026	0.00037448508301086	0
Sphingobacterium(100)	Otu000456	0	0	0.00109075043630017	0.00037448508301086	0
uncultured(87)	Otu000461	0.000544959128065395	0	0	0	0.000331162379953637
unclassified(100)	Otu000464	0	0.000874781304673832	0	0	0
Pseudomonas(81)	Otu000468	0	0.000124968757810547	0	0	0
Rhizobium(100)	Otu000474	0.0000778513040093422	0.000124968757810547	0	0	0
unclassified(100)	Otu000475	0	0	0	0	0.000110387459984546
Aquabacterium(67)	Otu000478	0	0	0.000109075043630017	0	0
Aquabacterium(67)	Otu000479	0.000233553912028026	0	0	0	0
unclassified(67)	Otu000483	0	0	0	0	0.000993487139860912
Novosphingobium(96)	Otu000484	0.000155702608018684	0	0	0	0
Arcicella(100)	Otu000485	0	0.000249937515621095	0.000327225130890052	0	0
Sphingobium(100)	Otu000489	0.000155702608018684	0	0.000109075043630017	0	0
Novosphingobium(95)	Otu000491	0.0000778513040093422	0	0	0	0
Dokdonella(85)	Otu000492	0.0000778513040093422	0.000374906273431642	0	0	0
Novosphingobium(85)	Otu000493	0.0000778513040093422	0	0	0	0
unclassified(100)	Otu000497	0	0	0	0	0.000110387459984546
Flavobacterium(65)	Otu000501	0	0	0	0.00037448508301086	0
unclassified(100)	Otu000504	0	0.000124968757810547	0	0	0
unclassified(100)	Otu000505	0.0000778513040093422	0	0.000109075043630017	0	0
unclassified(100)	Otu000510	0	0	0	0.00012482836100362	0

Aquabacterium(69)	Otu000511	0.0000778513040093422	0	0	0	0
Rhizobacter(95)	Otu000512	0	0	0	0	0.000110387459984546
Anaerococcus(100)	Otu000513	0	0	0	0.00049931344401448	0.00298046141958274
Flavobacterium(95)	Otu000516	0	0.000124968757810547	0	0	0
Anaerococcus(100)	Otu000518	0	0.00624843789052737	0	0	0
Paracoccus(64)	Otu000532	0	0	0	0.00037448508301086	0
unclassified(100)	Otu000534	0	0.000999750062484379	0.000654450261780105	0	0.000883099679876366
Sphingobium(95)	Otu000535	0.0000778513040093422	0	0.000109075043630017	0.00012482836100362	0.000110387459984546
unclassified(100)	Otu000536	0.000233553912028026	0	0.000545375218150087	0	0
unclassified(53)	Otu000540	0.0000778513040093422	0	0.000109075043630017	0	0
unclassified	Otu000542	0.000622810432074737	0	0	0.00074897016602172	0
Rhizobium(100)	Otu000543	0.000155702608018684	0	0.000109075043630017	0	0.000110387459984546
Dysgonomonas(100)	Otu000545	0	0	0.000109075043630017	0	0
unclassified(100)	Otu000547	0.000233553912028026	0.000124968757810547	0	0	0
Legionella(100)	Otu000548	0.000155702608018684	0	0.00043630017452007	0	0.000331162379953637
unclassified(100)	Otu000550	0	0.000124968757810547	0	0.00012482836100362	0
Brevundimonas(95)	Otu000552	0	0	0	0.00012482836100362	0
Streptococcus(100)	Otu000553	0.0000778513040093422	0.000124968757810547	0	0	0
Gluconobacter(100)	Otu000554	0	0	0	0	0.000883099679876366
Chryseobacterium(100)	Otu000556	0	0	0.000109075043630017	0	0
Bergeyella(100)	Otu000557	0.0000778513040093422	0.00337415646088478	0	0.0056172762451629	0
Rhizobium(100)	Otu000560	0	0	0	0.00024965672200724	0.000110387459984546
Chryseobacterium(100)	Otu000562	0	0	0.000109075043630017	0	0
unclassified	Otu000563	0	0	0	0	0.000110387459984546
Caulobacter(73)	Otu000565	0.0000778513040093422	0	0	0	0
Phyllobacterium(100)	Otu000567	0	0.0021244688827793	0	0.00012482836100362	0
Dysgonomonas(100)	Otu000571	0	0	0	0.00012482836100362	0
Sphingopyxis(59)	Otu000579	0.000155702608018684	0	0.00043630017452007	0.00012482836100362	0
unclassified(100)	Otu000580	0.0000778513040093422	0	0.000218150087260035	0	0
unclassified(100)	Otu000583	0	0.000124968757810547	0	0	0
unclassified(100)	Otu000584	0	0	0.000218150087260035	0	0.000220774919969092
Pseudomonas(100)	Otu000586	0	0	0	0.00012482836100362	0
Paracoccus(95)	Otu000596	0	0	0	0	0.000110387459984546

Paracoccus(100)	Otu000597	0	0	0	0.00012482836100362	0
unclassified(71)	Otu000601	0.0000778513040093422	0	0.000109075043630017	0	0
Lacibacter(100)	Otu000610	0	0	0.000327225130890052	0	0
unclassified(100)	Otu000618	0	0	0	0.00012482836100362	0
Paracoccus(94)	Otu000620	0	0.000124968757810547	0	0.00012482836100362	0
unclassified(100)	Otu000628	0	0.000124968757810547	0	0	0
Lactococcus(100)	Otu000633	0.0000778513040093422	0.00199950012496876	0	0.00037448508301086	0
Sphingomonas(100)	Otu000635	0	0.000874781304673832	0.000109075043630017	0.00037448508301086	0
Sphingobium(100)	Otu000636	0.0000778513040093422	0	0	0	0
Lactobacillus(100)	Otu000640	0	0.000624843789052737	0	0.00237173885906878	0
Flexibacter(100)	Otu000645	0	0	0	0	0.000110387459984546
unclassified(100)	Otu000646	0	0	0.000218150087260035	0	0
Methylobacterium(100)	Otu000648	0.000467107824056053	0	0.000109075043630017	0.00399450755211584	0
Dysgonomonas(100)	Otu000655	0	0	0.000109075043630017	0	0
Dysgonomonas(100)	Otu000659	0	0	0	0.00012482836100362	0
Dysgonomonas(100)	Otu000660	0	0.000124968757810547	0	0	0
unclassified(80)	Otu000665	0	0	0	0	0.000110387459984546
unclassified(100)	Otu000673	0	0.000124968757810547	0	0	0.000220774919969092
Bacillus(100)	Otu000674	0	0	0	0.00012482836100362	0
uncultured(100)	Otu000678	0	0	0.000327225130890052	0.00012482836100362	0.000110387459984546
unclassified(100)	Otu000680	0	0	0	0.00012482836100362	0
Staphylococcus(100)	Otu000682	0.000233553912028026	0	0	0	0
uncultured_bacterium(87)	Otu000684	0	0	0.000218150087260035	0.00149794033204344	0
Patulibacter(100)	Otu000685	0	0.000124968757810547	0.000109075043630017	0	0
Hyphomicrobium(87)	Otu000688	0	0	0.00283595113438045	0	0.00176619935975273
Roseomonas(87)	Otu000695	0.0000778513040093422	0.000749812546863284	0.00141797556719023	0	0.000883099679876366
unclassified(100)	Otu000701	0	0	0	0.00012482836100362	0
Perlucidibaca(86)	Otu000707	0	0	0.000545375218150087	0.00024965672200724	0
unclassified	Otu000711	0	0	0	0.00012482836100362	0.000110387459984546
Aquabacterium(93)	Otu000713	0	0	0.000218150087260035	0	0.000110387459984546
Novosphingobium(93)	Otu000718	0.0000778513040093422	0	0	0	0
Sphingomonas(100)	Otu000720	0	0	0	0.00112345524903258	0.000441549839938183
Bacillus(100)	Otu000726	0	0	0	0.00024965672200724	0

Chryseobacterium(100)	Otu000731	0	0.000124968757810547	0	0	0
Flexibacter(100)	Otu000733	0.000233553912028026	0	0.00043630017452007	0.00274622394207964	0.000883099679876366
Caedibacter(100)	Otu000738	0.0000778513040093422	0	0.000109075043630017	0	0
Pedobacter(79)	Otu000739	0	0	0.000109075043630017	0	0.000220774919969092
Sphingomonas(100)	Otu000740	0	0	0	0.00074897016602172	0
Novosphingobium(100)	Otu000743	0	0.000499875031242189	0	0	0
Roseomonas(100)	Otu000750	0	0	0	0.00012482836100362	0
Sphingobacterium(100)	Otu000752	0	0	0	0.00049931344401448	0
unclassified(100)	Otu000757	0	0	0	0.00012482836100362	0
Cytophaga(100)	Otu000759	0	0	0.00883507853403141	0	0.00353239871950546
Enterococcus(100)	Otu000760	0	0.000374906273431642	0	0	0
Fusobacterium(100)	Otu000766	0	0	0	0.00037448508301086	0
unclassified(70)	Otu000770	0.000155702608018684	0	0	0	0
unclassified(100)	Otu000787	0	0	0.000109075043630017	0	0
Afipia(100)	Otu000794	0.000544959128065395	0	0.000109075043630017	0	0
unclassified(70)	Otu000804	0	0	0	0.0006241418050181	0
Flavobacterium(100)	Otu000805	0	0.000124968757810547	0	0	0
Chryseobacterium(100)	Otu000809	0	0	0.000109075043630017	0	0
unclassified(100)	Otu000811	0	0	0.000981675392670157	0	0
unclassified(100)	Otu000812	0	0	0.000109075043630017	0	0
Prevotella(100)	Otu000816	0	0	0.00109075043630017	0	0
unclassified(100)	Otu000818	0	0	0	0.00012482836100362	0
Hydrotalea(100)	Otu000823	0.0000778513040093422	0	0	0	0
Legionella(100)	Otu000825	0	0.0044988752811797	0	0	0
Novosphingobium(100)	Otu000831	0.0000778513040093422	0	0	0	0
unclassified(92)	Otu000839	0	0	0	0	0.000110387459984546
Rhodoplanes(100)	Otu000842	0.0000778513040093422	0	0	0	0
unclassified(100)	Otu000843	0	0	0	0.00012482836100362	0
Prevotella(100)	Otu000847	0	0.000749812546863284	0	0	0
unclassified(92)	Otu000850	0	0	0	0	0.000883099679876366
Chryseobacterium(100)	Otu000851	0	0.000124968757810547	0	0	0
Novosphingobium(100)	Otu000855	0.0000778513040093422	0	0	0	0
Lactobacillus(100)	Otu000857	0	0	0.000218150087260035	0	0

Dysgonomonas(92)	Otu000860	0	0	0.000109075043630017	0.00024965672200724	0
Dysgonomonas(100)	Otu000869	0	0.000124968757810547	0	0	0
Anaerococcus(100)	Otu000876	0	0.000124968757810547	0	0	0
Nevskia(100)	Otu000881	0	0.000124968757810547	0.000109075043630017	0	0.000110387459984546
Arenimonas(100)	Otu000885	0	0	0.000218150087260035	0.00037448508301086	0
unclassified(100)	Otu000889	0	0	0	0	0.000220774919969092
Roseomonas(100)	Otu000890	0	0.000499875031242189	0.000654450261780105	0	0
unclassified(100)	Otu000891	0	0.000124968757810547	0	0	0.000110387459984546
Dysgonomonas(82)	Otu000896	0	0	0.000109075043630017	0	0
Dysgonomonas(100)	Otu000897	0	0.000124968757810547	0	0	0
unclassified(100)	Otu000904	0	0.000124968757810547	0.000109075043630017	0	0
Bosea(100)	Otu000905	0	0	0.000109075043630017	0	0
unclassified(82)	Otu000911	0	0	0	0.00012482836100362	0
Rhizobium(100)	Otu000916	0	0	0	0	0.000110387459984546
unclassified(100)	Otu000917	0	0	0	0	0.00077271221989182
Flavobacterium(100)	Otu000919	0	0	0	0.00012482836100362	0
Capnocytophaga(100)	Otu000923	0	0.00149962509372657	0	0	0
unclassified(100)	Otu000929	0	0.00174956260934766	0.000763525305410122	0.00012482836100362	0.000662324759907275
unclassified(91)	Otu000936	0	0	0.000109075043630017	0	0
Novosphingobium(82)	Otu000944	0.0000778513040093422	0	0	0	0
unclassified(64)	Otu000954	0.000155702608018684	0	0	0	0
unclassified(100)	Otu000955	0.0000778513040093422	0	0	0	0
unclassified(100)	Otu000958	0	0	0.000109075043630017	0	0
Lacibacter(100)	Otu000960	0	0.000124968757810547	0	0	0
Lacibacter(100)	Otu000961	0	0	0.000327225130890052	0	0.000110387459984546
Pelomonas(61)	Otu000965	0.0000778513040093422	0	0	0.00012482836100362	0
Lacibacter(100)	Otu000981	0	0	0.000218150087260035	0	0
unclassified(100)	Otu000982	0.000155702608018684	0	0	0	0
Devosia(100)	Otu000984	0	0.00124968757810547	0	0	0
Roseomonas(100)	Otu000990	0	0	0.000109075043630017	0	0
unclassified(100)	Otu000993	0	0	0.000327225130890052	0	0
unclassified(100)	Otu000995	0.000233553912028026	0	0	0.00012482836100362	0
Dysgonomonas(100)	Otu000997	0	0	0.000218150087260035	0	0

Dysgonomonas(100)	Otu000998	0	0	0	0.00012482836100362	0
unclassified(100)	Otu001007	0	0	0.000218150087260035	0	0
uncultured_Stigmatella_sp.(70)	Otu001023	0.000467107824056053	0.000874781304673832	0.00174520069808028	0	0.00187658681973728
Fusobacterium(100)	Otu001029	0	0	0	0.00162276869304706	0
Halomonas(100)	Otu001032	0	0	0.000109075043630017	0.00074897016602172	0
Amaricoccus(100)	Otu001033	0	0	0	0.00299588066408688	0
unclassified(90)	Otu001036	0	0.000124968757810547	0	0	0.000110387459984546
Flavobacterium(80)	Otu001041	0	0	0	0.00287105230308326	0
Chryseobacterium(90)	Otu001047	0	0.000124968757810547	0	0	0
Chryseobacterium(100)	Otu001050	0	0	0.000109075043630017	0	0
Ralstonia(100)	Otu001052	0.0000778513040093422	0	0	0	0
Anaerococcus(100)	Otu001054	0	0	0	0.00037448508301086	0
Novosphingobium(90)	Otu001057	0.000155702608018684	0	0	0	0
Novosphingobium(100)	Otu001058	0.000233553912028026	0	0	0	0.000110387459984546
Sphingobium(100)	Otu001061	0	0	0	0.00012482836100362	0
Novosphingobium(90)	Otu001062	0	0.000124968757810547	0	0	0
Novosphingobium(100)	Otu001063	0.000467107824056053	0	0	0	0
Rhizobiales_bacterium_NHI-8(70)	Otu001065	0	0	0	0	0.000110387459984546
unclassified(80)	Otu001070	0.000155702608018684	0	0	0	0
Aquabacterium(100)	Otu001072	0	0	0.000109075043630017	0	0
Aquabacterium(80)	Otu001073	0	0.000124968757810547	0	0	0.000110387459984546
Tepidimonas(100)	Otu001074	0	0	0	0.00012482836100362	0
Aquabacterium(100)	Otu001076	0.0000778513040093422	0	0	0	0
Aquabacterium(90)	Otu001078	0	0	0.000109075043630017	0	0
Altererythrobacter(61)	Otu001080	0	0	0	0.00012482836100362	0
unclassified(100)	Otu001091	0.0000778513040093422	0	0	0	0.000110387459984546
Gluconobacter(100)	Otu001093	0	0	0	0	0.000331162379953637
Ochrobactrum(100)	Otu001095	0	0.000124968757810547	0.000109075043630017	0	0
Caedibacter(89)	Otu001100	0.000233553912028026	0	0	0	0
Caedibacter(100)	Otu001101	0.0000778513040093422	0	0	0	0
Halomonas(100)	Otu001103	0	0.000874781304673832	0	0	0
Alkalibacterium(100)	Otu001104	0	0.000124968757810547	0	0	0
Lachnoanaerobaculum(100)	Otu001106	0	0	0	0	0.00143503697979909

unclassified(78)	Otu001110	0	0	0	0.0024965672200724	0.00077271221989182
Bosea(100)	Otu001113	0	0	0.000218150087260035	0	0
Rhodopseudomonas(100)	Otu001114	0.000233553912028026	0	0	0	0.000551937299922729
Acinetobacter(100)	Otu001116	0	0	0	0	0.000110387459984546
unclassified(100)	Otu001124	0	0	0	0	0.000110387459984546
Brevundimonas(100)	Otu001128	0	0	0	0.00012482836100362	0
Dysgonomonas(100)	Otu001130	0	0.000124968757810547	0	0	0
Dysgonomonas(100)	Otu001133	0	0.000124968757810547	0	0	0
Schlegelella(100)	Otu001136	0	0	0.00043630017452007	0	0.000441549839938183
Aquabacterium(89)	Otu001138	0	0	0.000109075043630017	0	0
unclassified(100)	Otu001141	0.0000778513040093422	0	0	0	0
Flavobacterium(100)	Otu001147	0	0	0.00109075043630017	0.00012482836100362	0
Flavobacterium(100)	Otu001150	0	0	0	0.00024965672200724	0
unclassified(100)	Otu001173	0.0000778513040093422	0	0	0	0
unclassified(78)	Otu001183	0	0	0	0	0.00165581189976819
Ferruginibacter(100)	Otu001184	0	0	0.00043630017452007	0	0
unclassified(56)	Otu001190	0.000311405216037369	0	0	0	0
unclassified(100)	Otu001195	0	0.00224943764058985	0	0	0
unclassified(100)	Otu001196	0.0000778513040093422	0	0.000109075043630017	0	0
Pseudomonas(100)	Otu001198	0	0.000124968757810547	0	0	0.000110387459984546
Methylobacterium(100)	Otu001200	0.0000778513040093422	0	0	0	0
Asaia(56)	Otu001211	0	0	0	0	0.000110387459984546
unclassified(89)	Otu001212	0	0	0	0.00049931344401448	0
unclassified(100)	Otu001219	0	0	0.000109075043630017	0	0
Abiotrophia(100)	Otu001225	0	0	0	0.00137311197103982	0
Candidatus_Rhabdochlamydia(100)	Otu001229	0.0000778513040093422	0	0	0	0
Gluconobacter(75)	Otu001248	0	0	0	0	0.000331162379953637
unclassified(100)	Otu001263	0	0	0	0	0.000110387459984546
Rubellimicrobium(100)	Otu001275	0	0	0	0	0.00132464951981455
unclassified(63)	Otu001283	0	0	0	0	0.000110387459984546
unclassified(100)	Otu001284	0.0000778513040093422	0	0	0	0
unclassified(63)	Otu001285	0	0	0	0	0.000110387459984546
Cloacibacterium(100)	Otu001288	0	0.000124968757810547	0	0	0

Chryseobacterium(88)	Otu001289	0	0	0	0.00012482836100362	0
unclassified(100)	Otu001299	0	0	0.000109075043630017	0	0
Phenylobacterium(88)	Otu001300	0	0.000124968757810547	0	0	0
Flavobacterium(100)	Otu001303	0	0.000124968757810547	0	0	0
Parvimonas(100)	Otu001305	0.0000778513040093422	0	0	0	0
Blastomonas(100)	Otu001308	0.000700661736084079	0	0	0	0
Sphingopyxis(88)	Otu001311	0.0000778513040093422	0	0	0	0
Sphingomonas(100)	Otu001312	0.000155702608018684	0	0	0	0
Novosphingobium(100)	Otu001313	0.000155702608018684	0	0	0	0
Sphingomonas(100)	Otu001315	0	0	0	0.0018724254150543	0
Nubsella(63)	Otu001333	0	0	0	0	0.00132464951981455
unclassified(100)	Otu001336	0.000155702608018684	0	0	0	0
Pedobacter(100)	Otu001350	0	0	0	0.00012482836100362	0
unclassified(100)	Otu001353	0	0.000124968757810547	0.000109075043630017	0	0
unclassified(100)	Otu001357	0	0.000124968757810547	0	0	0
Veillonella(100)	Otu001369	0	0	0.000545375218150087	0	0
Dysgonomonas(100)	Otu001372	0	0	0.000218150087260035	0.00012482836100362	0
Ochrobactrum(100)	Otu001375	0	0	0	0.00012482836100362	0
Hyphomicrobium(100)	Otu001387	0	0	0.000109075043630017	0	0
unclassified(100)	Otu001391	0	0	0	0	0.000220774919969092
Bosea(100)	Otu001404	0	0	0.00087260034904014	0	0.000220774919969092
Pseudomonas(100)	Otu001405	0	0	0	0.00012482836100362	0
unclassified(100)	Otu001414	0.000155702608018684	0	0	0	0
Pseudomonas(75)	Otu001418	0	0	0	0	0.000110387459984546
Rubellimicrobium(86)	Otu001420	0	0.000499875031242189	0	0	0
unclassified(72)	Otu001423	0	0	0.000763525305410122	0	0
unclassified(100)	Otu001426	0	0	0	0	0.000441549839938183
unclassified	Otu001434	0	0	0.00239965095986038	0	0
unclassified(72)	Otu001440	0	0	0	0	0.000220774919969092
Dysgonomonas(100)	Otu001445	0	0.000124968757810547	0	0	0
Dysgonomonas(100)	Otu001447	0	0	0	0	0.000110387459984546
unclassified(100)	Otu001452	0	0	0	0.00012482836100362	0
unclassified(100)	Otu001462	0	0	0.00141797556719023	0	0

unclassified(100)	Otu001474	0	0	0	0	0.000110387459984546
unclassified(100)	Otu001476	0	0	0	0	0.000220774919969092
unclassified(100)	Otu001480	0	0.000124968757810547	0	0	0
unclassified(100)	Otu001484	0	0	0.000109075043630017	0	0
Hymenobacter(100)	Otu001491	0	0.00112471882029493	0	0	0
Rhodopseudomonas(58)	Otu001499	0.000155702608018684	0	0	0	0
unclassified(100)	Otu001528	0	0.000124968757810547	0	0	0
unclassified(100)	Otu001536	0	0.00137465633591602	0.00043630017452007	0.00037448508301086	0.000331162379953637
Aquabacterium(86)	Otu001541	0	0.000124968757810547	0	0	0
Sphingomonas(100)	Otu001559	0	0	0	0.00037448508301086	0.000993487139860912
Anaerococcus(100)	Otu001566	0	0	0	0.00012482836100362	0
Staphylococcus(100)	Otu001571	0	0	0	0.00012482836100362	0
Jeotgalicoccus(86)	Otu001572	0	0	0	0.00012482836100362	0
Pseudomonas(72)	Otu001578	0	0	0	0.0006241418050181	0
Chryseobacterium(100)	Otu001592	0	0.000749812546863284	0	0	0
Stenotrophomonas(100)	Otu001595	0	0.000124968757810547	0	0	0
unclassified(100)	Otu001603	0.0000778513040093422	0	0	0	0
unclassified(100)	Otu001609	0	0.00249937515621095	0.00043630017452007	0	0
Chryseobacterium(100)	Otu001637	0	0	0	0	0.000110387459984546
Flavobacterium(100)	Otu001638	0	0	0	0	0.00110387459984546
Hyphomicrobium(100)	Otu001645	0	0	0	0.00174759705405068	0
Deinococcus(100)	Otu001659	0	0	0	0	0.000110387459984546
uncultured(100)	Otu001662	0.000155702608018684	0	0	0	0.00110387459984546
Caedibacter(100)	Otu001663	0.0000778513040093422	0	0	0	0
uncultured(100)	Otu001665	0	0	0	0.00099862688802896	0
Roseburia(100)	Otu001721	0	0.000124968757810547	0	0	0
Blautia(100)	Otu001723	0	0	0	0	0.000220774919969092
Anaerostipes(100)	Otu001725	0	0	0	0	0.000331162379953637
Bacteroides(100)	Otu001726	0	0	0	0	0.000110387459984546
Staphylococcus(84)	Otu001728	0	0	0	0.00012482836100362	0
Pedomicrobium(100)	Otu001731	0	0.00112471882029493	0.000763525305410122	0	0.000331162379953637
Devosia(100)	Otu001734	0	0	0	0.00037448508301086	0
unclassified	Otu001739	0	0.000124968757810547	0	0	0

unclassified(100)	Otu001747	0	0.000124968757810547	0.000109075043630017	0	0
unclassified(100)	Otu001748	0.0000778513040093422	0	0	0	0
unclassified(84)	Otu001749	0	0	0	0.00012482836100362	0
unclassified(100)	Otu001761	0	0	0	0	0.000110387459984546
Rhodobium(100)	Otu001773	0	0.000624843789052737	0	0	0.000331162379953637
uncultured(100)	Otu001775	0	0	0	0.00024965672200724	0
Hymenobacter(100)	Otu001777	0	0	0	0	0.00132464951981455
Flexibacter(100)	Otu001785	0	0	0.000109075043630017	0	0
Hymenobacter(100)	Otu001787	0	0	0	0	0.000883099679876366
Cytophaga(100)	Otu001789	0	0	0	0.00037448508301086	0
Dyadobacter(100)	Otu001790	0	0.000749812546863284	0	0	0
Hymenobacter(100)	Otu001794	0	0	0.0025087260034904	0	0
unclassified(84)	Otu001798	0	0	0	0	0.00761673473893366
Bdellovibrio(100)	Otu001801	0	0.000874781304673832	0	0	0
Chryseobacterium(100)	Otu001804	0	0.000124968757810547	0	0	0
Chryseobacterium(100)	Otu001805	0	0	0	0	0.000883099679876366
Chryseobacterium(100)	Otu001806	0	0	0	0.00012482836100362	0.00143503697979909
unclassified(100)	Otu001853	0	0.000499875031242189	0	0	0
Prevotella(100)	Otu001865	0	0	0.000109075043630017	0	0
Marinomonas(100)	Otu001870	0	0	0	0.00099862688802896	0
Lysobacter(84)	Otu001886	0.0000778513040093422	0	0	0	0
unclassified(100)	Otu001905	0	0.000124968757810547	0	0	0
Pedobacter(100)	Otu001911	0	0.000249937515621095	0	0	0
unclassified(100)	Otu001912	0	0	0.000109075043630017	0	0
Dysgonomonas(100)	Otu001919	0	0	0.000109075043630017	0	0
unclassified(67)	Otu001920	0	0	0	0	0.000331162379953637
Chryseobacterium(100)	Otu001922	0	0	0	0.00012482836100362	0
Chryseobacterium(100)	Otu001928	0	0	0	0.00012482836100362	0
Reyranella(100)	Otu001934	0.000389256520046711	0	0	0	0
unclassified(67)	Otu001942	0.000311405216037369	0	0	0	0
Sphingopyxis(84)	Otu001952	0	0	0.000109075043630017	0	0
Sphingomonas(100)	Otu001959	0	0.000874781304673832	0	0	0
Methylobacterium(100)	Otu001960	0	0.000624843789052737	0	0	0

Methylobacterium(100)	Otu001961	0.0000778513040093422	0	0	0.00024965672200724	0
Dysgonomonas(100)	Otu001965	0	0	0	0.00012482836100362	0.000110387459984546
Chryseobacterium(100)	Otu001973	0	0	0	0	0.000110387459984546
Aquabacterium(84)	Otu001990	0	0	0.000109075043630017	0	0
Aquabacterium(67)	Otu001991	0	0	0.000109075043630017	0	0
unclassified(100)	Otu001998	0	0	0	0	0.000220774919969092
unclassified(84)	Otu001999	0.0000778513040093422	0	0	0	0
unclassified(67)	Otu002005	0	0.000124968757810547	0	0	0
unclassified(100)	Otu002012	0	0	0	0.00024965672200724	0.000662324759907275
Alkanindiges(67)	Otu002015	0	0	0	0.00012482836100362	0
Aeromonas(100)	Otu002028	0	0	0	0.00012482836100362	0
unclassified(100)	Otu002046	0	0	0	0	0.000110387459984546
unclassified(100)	Otu002047	0.0000778513040093422	0	0	0	0
Aquabacterium(84)	Otu002048	0	0	0.000109075043630017	0	0
Rubellimicrobium(100)	Otu002051	0	0	0	0.00024965672200724	0
Rubellimicrobium(100)	Otu002053	0	0	0	0	0.000551937299922729
Rubellimicrobium(84)	Otu002056	0	0	0	0.00162276869304706	0.000220774919969092
Bergeyella(100)	Otu002061	0	0.000124968757810547	0	0	0
Aquabacterium(100)	Otu002072	0	0	0.000109075043630017	0	0
Aquabacterium(100)	Otu002075	0.0000778513040093422	0	0	0	0
Aquabacterium(80)	Otu002087	0.0000778513040093422	0	0	0	0
unclassified(61)	Otu002090	0	0	0	0	0.00242852411966001
Aquabacterium(100)	Otu002096	0	0	0	0	0.000110387459984546
unclassified(61)	Otu002102	0	0	0	0	0.000331162379953637
unclassified(100)	Otu002123	0	0.000124968757810547	0	0	0
Paracoccus(100)	Otu002138	0	0	0	0	0.000551937299922729
unclassified(100)	Otu002147	0	0	0	0	0.000662324759907275
unclassified(100)	Otu002154	0	0	0.000109075043630017	0	0
unclassified(100)	Otu002169	0	0	0	0	0.000110387459984546
Methylobacterium(100)	Otu002196	0.0000778513040093422	0	0	0	0
Brevundimonas(100)	Otu002210	0	0	0	0.00012482836100362	0
Caulobacter(80)	Otu002213	0.000155702608018684	0	0	0	0
Legionella(100)	Otu002258	0	0.000749812546863284	0	0	0

Legionella(100)	Otu002262	0	0	0.000327225130890052	0	0
Legionella(100)	Otu002263	0	0	0	0.00024965672200724	0
unclassified(100)	Otu002281	0	0	0	0	0.000220774919969092
Lactococcus(100)	Otu002287	0	0	0	0.0006241418050181	0
Pseudomonas(100)	Otu002299	0	0.000124968757810547	0	0	0
Pseudomonas(100)	Otu002306	0	0	0.000109075043630017	0	0.000110387459984546
Novosphingobium(61)	Otu002320	0.0000778513040093422	0	0	0	0
Sphingomonas(100)	Otu002332	0	0	0	0	0.000110387459984546
unclassified(80)	Otu002333	0	0	0	0	0.000110387459984546
Sphingopyxis(100)	Otu002336	0	0	0	0	0.000110387459984546
Sphingomonas(80)	Otu002337	0	0	0.000109075043630017	0	0
unclassified(61)	Otu002338	0.0000778513040093422	0	0	0	0
Sphingomonas(100)	Otu002352	0.0000778513040093422	0	0	0	0
Acinetobacter(100)	Otu002360	0	0	0	0.00012482836100362	0
Dysgonomonas(100)	Otu002363	0	0	0	0.00012482836100362	0
Dysgonomonas(100)	Otu002368	0	0	0.000109075043630017	0	0
Dysgonomonas(100)	Otu002369	0	0	0	0	0.000110387459984546
Porphyromonas(100)	Otu002371	0.0000778513040093422	0	0	0	0
Dysgonomonas(100)	Otu002373	0	0.000124968757810547	0	0	0
Dysgonomonas(100)	Otu002381	0	0.000124968757810547	0	0	0
Afipia(100)	Otu002383	0.0000778513040093422	0	0	0	0.000110387459984546
unclassified(61)	Otu002396	0.000155702608018684	0	0	0	0
Rhizobium(100)	Otu002407	0.0000778513040093422	0	0	0	0
unclassified(100)	Otu002431	0	0.000124968757810547	0	0	0
unclassified(100)	Otu002441	0.0014791747761775	0	0	0	0
Ochrobactrum(100)	Otu002443	0	0.000124968757810547	0	0	0
Rhizobium(100)	Otu002445	0	0	0	0	0.000110387459984546
Capnocytophaga(100)	Otu002489	0	0.000999750062484379	0	0	0
Catonella(100)	Otu002503	0	0	0	0	0.000110387459984546
Chryseobacterium(100)	Otu002527	0.0000778513040093422	0	0	0.00037448508301086	0
Ralstonia(80)	Otu002537	0.0000778513040093422	0	0	0	0
Prevotella(100)	Otu002544	0	0	0	0.00087379852702534	0
Chryseobacterium(100)	Otu002561	0	0.000124968757810547	0	0	0

Lacibacter(100)	Otu002566	0	0	0.000109075043630017	0	0
Sphingobacterium(80)	Otu002589	0	0	0	0	0.000220774919969092
unclassified(100)	Otu002599	0	0.00124968757810547	0	0	0
Hymenobacter(100)	Otu002622	0	0	0	0.00012482836100362	0
Hymenobacter(100)	Otu002623	0	0.000874781304673832	0	0.00024965672200724	0
Cytophaga(100)	Otu002624	0	0	0.000109075043630017	0	0
unclassified(100)	Otu002659	0	0	0	0.00012482836100362	0
unclassified(100)	Otu002660	0	0	0	0	0.000110387459984546
Dysgonomonas(100)	Otu002662	0	0	0.000109075043630017	0	0
unclassified(100)	Otu002668	0	0	0.000109075043630017	0	0
unclassified(100)	Otu002673	0	0	0	0	0.000110387459984546
Dysgonomonas(100)	Otu002684	0	0.000124968757810547	0	0	0
uncultured(100)	Otu002696	0	0.000499875031242189	0	0	0
Bergeyella(100)	Otu002722	0	0	0	0.00012482836100362	0
Legionella(100)	Otu002727	0	0.00124968757810547	0	0	0
unclassified(100)	Otu002733	0	0	0.000109075043630017	0	0
unclassified(100)	Otu002754	0	0.000124968757810547	0	0	0
unclassified(100)	Otu002755	0	0	0.000109075043630017	0	0
Legionella(100)	Otu002762	0	0	0	0	0.000331162379953637
Dysgonomonas(100)	Otu002768	0	0	0.000109075043630017	0	0
unclassified	Otu002783	0	0	0	0	0.000110387459984546
Aquabacterium(75)	Otu002786	0.0000778513040093422	0	0	0	0
Aquabacterium(100)	Otu002792	0.0000778513040093422	0	0	0	0
Dysgonomonas(100)	Otu002804	0	0.000124968757810547	0	0	0
Dysgonomonas(100)	Otu002809	0	0	0.000109075043630017	0	0
Dysgonomonas(100)	Otu002810	0	0.000124968757810547	0	0	0
unclassified(100)	Otu002819	0	0.000124968757810547	0	0	0
Aquabacterium(75)	Otu002843	0	0	0	0	0.000110387459984546
unclassified	Otu002849	0	0	0	0	0.000110387459984546
unclassified(100)	Otu002850	0.0000778513040093422	0	0	0	0
Dysgonomonas(100)	Otu002865	0	0.000124968757810547	0	0	0
Aquabacterium(100)	Otu002873	0	0	0	0	0.000110387459984546
unclassified	Otu002875	0	0	0	0	0.000110387459984546

unclassified(75)	Otu002879	0	0	0	0	0.000110387459984546
Aquabacterium(75)	Otu002890	0	0	0.000109075043630017	0	0
Dysgonomonas(100)	Otu002892	0	0	0	0	0.000110387459984546
unclassified(100)	Otu002894	0	0.000124968757810547	0	0	0
Aquabacterium(100)	Otu002901	0.0000778513040093422	0	0	0	0
Dysgonomonas(100)	Otu002905	0	0	0	0.00012482836100362	0
unclassified(100)	Otu002940	0.000155702608018684	0.000124968757810547	0.00152705061082024	0	0.000220774919969092
Leptotrichia(100)	Otu002947	0	0.000124968757810547	0	0.00012482836100362	0
Adhaeribacter(100)	Otu002968	0	0	0	0	0.000551937299922729
unclassified(100)	Otu003008	0	0	0	0.00012482836100362	0
Planctomyces(100)	Otu003027	0	0	0	0	0.000220774919969092
Gluconobacter(100)	Otu003048	0	0	0	0	0.000110387459984546
Bryobacter(100)	Otu003063	0.0000778513040093422	0.000249937515621095	0	0	0.000551937299922729
unclassified(100)	Otu003066	0	0	0	0	0.000110387459984546
unclassified(100)	Otu003069	0	0	0.000981675392670157	0	0.000110387459984546
unclassified(100)	Otu003070	0	0.000124968757810547	0	0	0
unclassified(100)	Otu003078	0	0	0	0	0.000110387459984546
unclassified(100)	Otu003089	0	0	0	0	0.000110387459984546
unclassified(100)	Otu003096	0	0	0	0.00012482836100362	0
unclassified(100)	Otu003117	0	0.000124968757810547	0	0	0
unclassified(100)	Otu003143	0.000155702608018684	0	0	0	0
Candidatus_Protochlamydia(100)	Otu003146	0	0	0	0.00049931344401448	0
unclassified(75)	Otu003185	0	0	0	0.00024965672200724	0.000331162379953637
unclassified(100)	Otu003226	0	0	0	0.0006241418050181	0
unclassified	Otu003228	0	0	0	0	0.000110387459984546
Prevotella(100)	Otu003233	0	0	0	0.00037448508301086	0
Prevotella(100)	Otu003251	0	0	0	0.00012482836100362	0
Sphingobium(100)	Otu003281	0	0	0	0.00024965672200724	0.000220774919969092
unclassified(75)	Otu003284	0.000155702608018684	0	0	0	0
unclassified	Otu003296	0	0	0	0.00012482836100362	0
Methylobacterium(100)	Otu003315	0	0	0	0	0.000110387459984546
uncultured(100)	Otu003322	0.0000778513040093422	0	0	0	0
Caedibacter(100)	Otu003323	0.0000778513040093422	0	0	0	0

Caedibacter(100)	Otu003324	0	0.00162459385153712	0	0	0
Pseudomonas(100)	Otu003342	0	0.000124968757810547	0	0	0
Lactobacillus(100)	Otu003373	0	0	0	0	0.000110387459984546
Lacibacter(100)	Otu003389	0	0	0.000109075043630017	0	0
Fluviicola(75)	Otu003417	0	0.00224943764058985	0	0	0
Pseudomonas(100)	Otu003443	0	0	0	0.00012482836100362	0
uncultured(100)	Otu003448	0	0	0	0.00012482836100362	0
Pseudochrobactrum(100)	Otu003456	0	0	0	0.00012482836100362	0
Aquabacterium(100)	Otu003458	0	0.000124968757810547	0	0	0
unclassified(100)	Otu003459	0	0.000499875031242189	0	0.00212208213706154	0
Brevundimonas(100)	Otu003478	0	0.000124968757810547	0	0	0
Brevundimonas(100)	Otu003479	0	0	0	0.00012482836100362	0
Caulobacter(75)	Otu003481	0.000155702608018684	0	0	0	0
unclassified(75)	Otu003482	0.0000778513040093422	0	0	0	0
unclassified(100)	Otu003501	0	0	0	0	0.000110387459984546
unclassified(100)	Otu003525	0	0	0.000109075043630017	0	0
Aureimonas(100)	Otu003537	0	0.000249937515621095	0	0	0.000220774919969092
unclassified(100)	Otu003543	0	0	0.000109075043630017	0	0.000110387459984546
unclassified(75)	Otu003548	0	0	0	0	0.000551937299922729
Novosphingobium(75)	Otu003557	0.0000778513040093422	0	0	0	0
unclassified(75)	Otu003558	0	0.000124968757810547	0	0	0
Chryseobacterium(100)	Otu003560	0	0	0	0.00012482836100362	0
unclassified(100)	Otu003562	0	0	0.000109075043630017	0	0
Bosea(100)	Otu003566	0	0	0	0	0.000110387459984546
Dysgonomonas(100)	Otu003568	0	0	0.000109075043630017	0	0
Sphingopyxis(100)	Otu003571	0	0	0	0.00012482836100362	0
Sphingobium(100)	Otu003574	0.000155702608018684	0	0	0	0
Sphingopyxis(100)	Otu003587	0	0.000374906273431642	0	0	0
Novosphingobium(100)	Otu003588	0.0000778513040093422	0	0	0	0
Chryseobacterium(100)	Otu003590	0	0.000124968757810547	0	0	0
unclassified(100)	Otu003600	0.0000778513040093422	0	0	0	0
Rhizobium(100)	Otu003605	0	0	0.00012482836100362	0
unclassified(100)	Otu003620	0	0	0	0.00012482836100362	0

unclassified(100)	Otu003643	0	0.000874781304673832	0	0	0
Devosia(100)	Otu003671	0	0	0	0.00037448508301086	0
unclassified(100)	Otu003679	0	0.000749812546863284	0	0	0
Hydrotalea(100)	Otu003683	0.000155702608018684	0	0	0	0
Terrimonas(100)	Otu003692	0	0	0	0.00037448508301086	0
Flavobacterium(100)	Otu003694	0	0.000124968757810547	0	0	0
Flavobacterium(100)	Otu003707	0	0.000124968757810547	0	0	0
Chryseobacterium(100)	Otu003716	0	0.000124968757810547	0	0	0
Chryseobacterium(100)	Otu003734	0	0.000124968757810547	0	0	0
Coprococcus(100)	Otu003764	0	0	0.000654450261780105	0	0
uncultured_Roseobacter_sp.(100)	Otu003773	0	0	0	0	0.000551937299922729
unclassified(100)	Otu003843	0	0.000249937515621095	0	0	0
Chryseobacterium(100)	Otu003868	0	0.000124968757810547	0	0	0
Aquabacterium(67)	Otu003888	0	0	0	0	0.000110387459984546
Halomonas(100)	Otu003901	0	0.000374906273431642	0	0	0
Halomonas(100)	Otu003902	0	0.000249937515621095	0	0	0
Dysgonomonas(100)	Otu003922	0	0	0	0	0.000110387459984546
Anaerobranca(100)	Otu003943	0	0.00112471882029493	0	0	0
Novosphingobium(100)	Otu003946	0.0000778513040093422	0	0	0	0
Rheinheimera(100)	Otu003947	0	0.000124968757810547	0	0	0
Dysgonomonas(100)	Otu003979	0	0	0	0.00012482836100362	0
Dysgonomonas(100)	Otu004009	0	0	0	0	0.000110387459984546
Dysgonomonas(100)	Otu004024	0	0.000124968757810547	0	0	0
unclassified(100)	Otu004056	0	0	0	0.00024965672200724	0
unclassified(100)	Otu004068	0.0000778513040093422	0	0	0	0
Aquabacterium(67)	Otu004081	0	0	0	0	0.000110387459984546
unclassified(100)	Otu004162	0	0.00112471882029493	0	0	0
unclassified(100)	Otu004175	0	0.000124968757810547	0	0	0
unclassified(67)	Otu004192	0	0	0	0	0.000110387459984546
unclassified(67)	Otu004195	0	0	0	0	0.000110387459984546
Simplicispira(100)	Otu004197	0	0	0	0	0.000110387459984546
Aquabacterium(100)	Otu004201	0.0000778513040093422	0	0	0	0
Pedobacter(100)	Otu004205	0	0	0.000109075043630017	0	0

Chryseobacterium(100)	Otu004216	0	0	0	0.00012482836100362	0
Sphingobacterium(100)	Otu004217	0	0	0	0.00024965672200724	0
unclassified(100)	Otu004226	0	0	0	0.00012482836100362	0
Aquabacterium(100)	Otu004270	0	0	0.000109075043630017	0	0
Negativicoccus(100)	Otu004271	0	0.000749812546863284	0.000109075043630017	0	0
Chryseobacterium(100)	Otu004274	0	0	0	0	0.000110387459984546
Aquabacterium(67)	Otu004286	0	0	0	0	0.000110387459984546
unclassified(100)	Otu004289	0	0	0	0	0.000110387459984546
unclassified(100)	Otu004316	0	0	0.000109075043630017	0	0
Afipia(67)	Otu004331	0.0000778513040093422	0	0	0	0
Bradyrhizobium(100)	Otu004332	0.0000778513040093422	0	0	0	0
Dysgonomonas(100)	Otu004376	0	0	0	0	0.000110387459984546
Dysgonomonas(100)	Otu004378	0	0	0.000109075043630017	0	0
Novosphingobium(100)	Otu004407	0.0000778513040093422	0	0	0	0
uncultured(100)	Otu004428	0	0	0	0	0.000220774919969092
Legionella(100)	Otu004440	0	0	0	0.00037448508301086	0
Novosphingobium(67)	Otu004452	0.0000778513040093422	0	0	0	0
Novosphingobium(100)	Otu004454	0	0	0	0	0.000110387459984546
Novosphingobium(100)	Otu004456	0.0000778513040093422	0	0	0	0
Pseudomonas(100)	Otu004477	0	0	0	0	0.000110387459984546
Rhodobacter(67)	Otu004505	0	0	0	0.00012482836100362	0
Staphylococcus(100)	Otu004519	0	0.000124968757810547	0	0	0
Haematobacter(100)	Otu004535	0	0	0	0.00049931344401448	0
Paracoccus(100)	Otu004539	0	0.000124968757810547	0	0	0
Aureimonas(100)	Otu004549	0	0	0	0	0.000993487139860912
unclassified(100)	Otu004559	0	0.000124968757810547	0	0	0
unclassified(100)	Otu004594	0	0.000124968757810547	0	0	0
unclassified(100)	Otu004607	0	0	0	0.00012482836100362	0
unclassified(100)	Otu004615	0	0.000124968757810547	0	0	0
Bacteroides(100)	Otu004717	0	0	0.00043630017452007	0	0
unclassified(100)	Otu004721	0	0	0	0.00012482836100362	0
Salinicoccus(67)	Otu004724	0	0	0	0	0.00110387459984546
Methylobacterium(100)	Otu004784	0	0	0	0.00074897016602172	0

Aeromonas(100)	Otu004793	0	0	0	0	0.000110387459984546
Aeromonas(100)	Otu004800	0	0	0.000109075043630017	0	0
unclassified(67)	Otu004837	0.0000778513040093422	0	0	0	0
unclassified(100)	Otu004840	0	0	0	0	0.000110387459984546
Aquabacterium(100)	Otu004843	0.0000778513040093422	0	0	0	0
unclassified(100)	Otu004856	0.0000778513040093422	0	0	0	0
Pseudomonas(100)	Otu004890	0	0	0	0	0.000110387459984546
Pseudomonas(67)	Otu004903	0	0	0	0	0.000110387459984546
Caulobacter(100)	Otu004955	0.0000778513040093422	0	0	0	0
Brevundimonas(100)	Otu004957	0	0	0.000109075043630017	0	0
Phenylobacterium(100)	Otu004961	0	0.000124968757810547	0	0	0
uncultured(100)	Otu004965	0	0	0	0.00012482836100362	0
unclassified(100)	Otu004982	0	0	0.000109075043630017	0	0
unclassified(100)	Otu004983	0	0.000124968757810547	0	0	0
Pseudomonas(100)	Otu004997	0	0	0.000109075043630017	0	0
Brevundimonas(100)	Otu005006	0	0	0	0	0.000110387459984546
Caulobacter(67)	Otu005013	0.0000778513040093422	0	0	0	0
Defluviicoccus(100)	Otu005014	0	0	0	0	0.00077271221989182
Brevundimonas(100)	Otu005021	0	0	0	0	0.000110387459984546
Brevundimonas(100)	Otu005023	0	0	0	0	0.000110387459984546
unclassified(67)	Otu005024	0.0000778513040093422	0	0	0	0
Aquabacterium(100)	Otu005032	0.0000778513040093422	0	0	0	0
Aquabacterium(100)	Otu005048	0	0	0	0	0.000110387459984546
Aquabacterium(100)	Otu005061	0	0	0.000109075043630017	0	0
Aquabacterium(100)	Otu005063	0	0	0.000109075043630017	0	0
uncultured(100)	Otu005164	0	0	0	0	0.000551937299922729
unclassified(100)	Otu005171	0.0000778513040093422	0	0	0	0
unclassified(100)	Otu005174	0	0	0.000327225130890052	0	0
unclassified(100)	Otu005185	0	0.000749812546863284	0	0	0
Roseomonas(100)	Otu005186	0	0	0	0.0006241418050181	0
unclassified(100)	Otu005204	0	0	0	0	0.000110387459984546
Gluconobacter(100)	Otu005227	0	0	0	0	0.000110387459984546
Rhizobium(100)	Otu005242	0	0	0	0	0.000110387459984546

Roseomonas(100)	Otu005251	0	0	0	0.00074897016602172	0
Tanticharoenia(67)	Otu005253	0	0	0	0	0.00231813665967546
Incertae_Sedis(100)	Otu005308	0	0	0	0.0018724254150543	0
Hydrotalea(67)	Otu005315	0.0000778513040093422	0	0	0	0
Ferruginibacter(100)	Otu005321	0	0	0.000218150087260035	0	0
unclassified(100)	Otu005324	0	0.000124968757810547	0	0	0
unclassified(100)	Otu005337	0	0	0	0	0.000110387459984546
unclassified(100)	Otu005339	0	0	0.000109075043630017	0	0
Incertae_Sedis(100)	Otu005346	0	0	0.000763525305410122	0	0
Bacillus(67)	Otu005356	0	0	0.00272687609075044	0	0
unclassified(100)	Otu005373	0.000155702608018684	0	0	0	0
unclassified(100)	Otu005376	0	0	0.000109075043630017	0	0
unclassified(100)	Otu005395	0.000155702608018684	0	0	0	0
unclassified(100)	Otu005408	0.0000778513040093422	0	0	0	0
Enterococcus(100)	Otu005442	0	0	0.000109075043630017	0	0
unclassified(100)	Otu005489	0	0	0	0.00012482836100362	0
Adhaeribacter(100)	Otu005503	0	0	0.00109075043630017	0	0
unclassified(100)	Otu005523	0	0	0	0.00012482836100362	0
unclassified(100)	Otu005619	0	0	0	0	0.000110387459984546
unclassified(100)	Otu005643	0	0	0	0.00074897016602172	0
Hymenobacter(100)	Otu005678	0	0	0	0.00012482836100362	0
unclassified(100)	Otu005712	0	0	0	0	0.000220774919969092
Dysgonomonas(100)	Otu005725	0	0.000124968757810547	0	0	0
Dysgonomonas(100)	Otu005737	0	0	0	0	0.000110387459984546
Incertae_Sedis(100)	Otu005835	0	0	0	0	0.000220774919969092
Phenylobacterium(100)	Otu005850	0	0	0	0.00012482836100362	0
unclassified	Otu005855	0	0	0	0.00012482836100362	0
Caulobacter(100)	Otu005862	0.0000778513040093422	0	0	0	0
Blastomonas(100)	Otu005869	0.0000778513040093422	0	0	0	0
unclassified(100)	Otu005894	0	0	0	0.00037448508301086	0
Aeromonas(100)	Otu005914	0	0	0	0	0.000110387459984546
Aeromonas(100)	Otu005951	0	0	0	0	0.000110387459984546
Defluviicoccus(100)	Otu005966	0	0	0	0.00037448508301086	0

unclassified(100)	Otu006006	0	0.000499875031242189	0	0	0
unclassified(100)	Otu006015	0.000155702608018684	0	0	0	0
Novosphingobium(100)	Otu006047	0.0000778513040093422	0	0	0	0
Sphingopyxis(100)	Otu006068	0.0000778513040093422	0	0	0	0
unclassified	Otu006107	0	0.000749812546863284	0	0	0
unclassified(100)	Otu006124	0.0000778513040093422	0	0	0	0
unclassified(100)	Otu006127	0	0	0	0	0.000110387459984546
unclassified(100)	Otu006391	0	0.000124968757810547	0	0	0
Fusobacterium(100)	Otu006413	0	0	0	0.00012482836100362	0
unclassified(100)	Otu006416	0	0	0	0.00012482836100362	0
unclassified(100)	Otu006445	0.0000778513040093422	0	0	0	0
unclassified(100)	Otu006473	0	0	0	0.00012482836100362	0
unclassified(100)	Otu006490	0	0	0.000109075043630017	0	0
Sphingobium(100)	Otu006502	0.0000778513040093422	0	0	0	0
unclassified(100)	Otu006509	0	0.000124968757810547	0	0	0
unclassified(100)	Otu006516	0	0	0.000109075043630017	0	0
unclassified(100)	Otu006615	0	0	0	0	0.000110387459984546
unclassified(100)	Otu006632	0.0000778513040093422	0	0	0	0
unclassified(100)	Otu006635	0	0	0	0	0.000110387459984546
unclassified(100)	Otu006672	0.0000778513040093422	0	0	0	0
unclassified(100)	Otu006691	0	0	0	0	0.000110387459984546
unclassified(100)	Otu006692	0	0.000124968757810547	0	0	0
unclassified(100)	Otu006719	0	0	0	0	0.000110387459984546
unclassified(100)	Otu006767	0.0000778513040093422	0	0	0	0
Pedobacter(100)	Otu006773	0	0.000124968757810547	0	0	0
Pedobacter(100)	Otu006775	0	0	0	0.00087379852702534	0
unclassified(100)	Otu006801	0.0000778513040093422	0	0	0	0
Cardiobacterium(100)	Otu006824	0	0.000374906273431642	0	0	0
unclassified(100)	Otu006861	0	0.000124968757810547	0	0	0
unclassified(100)	Otu006875	0.0000778513040093422	0	0	0	0
unclassified(100)	Otu006912	0	0	0	0.00012482836100362	0
unclassified(100)	Otu006996	0	0	0	0.00012482836100362	0
unclassified(100)	Otu007006	0.0000778513040093422	0	0	0	0

unclassified(100)	Otu007010	0	0.000124968757810547	0.000109075043630017	0	0
unclassified(100)	Otu007012	0	0	0	0	0.000110387459984546
unclassified(100)	Otu007039	0	0	0	0.00012482836100362	0
Dysgonomonas(100)	Otu007074	0	0.000124968757810547	0	0	0
Dysgonomonas(100)	Otu007092	0	0	0.000109075043630017	0	0
Dysgonomonas(100)	Otu007100	0	0	0	0	0.000110387459984546
Dysgonomonas(100)	Otu007134	0	0	0	0	0.000110387459984546
Dysgonomonas(100)	Otu007152	0	0.000124968757810547	0	0	0
Dysgonomonas(100)	Otu007182	0	0	0.000109075043630017	0	0
Dysgonomonas(100)	Otu007205	0	0.000124968757810547	0	0	0
Dysgonomonas(100)	Otu007229	0	0.000124968757810547	0	0	0
Dysgonomonas(100)	Otu007251	0	0	0	0	0.000110387459984546
Dysgonomonas(100)	Otu007257	0	0	0.000109075043630017	0	0
Dysgonomonas(100)	Otu007267	0	0	0	0.00012482836100362	0
Dysgonomonas(100)	Otu007281	0	0	0	0.00012482836100362	0
Dysgonomonas(100)	Otu007314	0	0.000124968757810547	0	0	0
Dysgonomonas(100)	Otu007317	0	0	0	0.00012482836100362	0
Dysgonomonas(100)	Otu007333	0	0.000124968757810547	0	0	0
unclassified	Otu007365	0	0	0	0.00012482836100362	0
Dysgonomonas(100)	Otu007391	0	0	0	0.00012482836100362	0
Dysgonomonas(100)	Otu007396	0	0	0	0.00012482836100362	0
Dysgonomonas(100)	Otu007423	0	0	0	0.00012482836100362	0
Dysgonomonas(100)	Otu007446	0	0.000124968757810547	0	0	0
Dysgonomonas(100)	Otu007452	0	0	0	0.00012482836100362	0
unclassified(100)	Otu007543	0	0	0	0.00012482836100362	0
Flavisolibacter(100)	Otu007571	0	0	0.00043630017452007	0	0.000551937299922729
Sphingomonas(100)	Otu007577	0	0	0	0.00012482836100362	0
unclassified(100)	Otu007597	0	0	0.000109075043630017	0	0
uncultured_bacterium(100)	Otu007609	0	0	0	0	0.000551937299922729
unclassified	Otu007629	0	0	0	0	0.000220774919969092
unclassified(100)	Otu007646	0	0	0	0.00012482836100362	0
unclassified(100)	Otu007658	0	0	0	0	0.000110387459984546
unclassified(100)	Otu007664	0	0	0	0	0.000331162379953637

unclassified(100)	Otu007667	0.0000778513040093422	0	0	0	0
unclassified	Otu007668	0	0	0	0.00099862688802896	0
unclassified	Otu007707	0.0000778513040093422	0	0	0	0
unclassified(100)	Otu007726	0.000155702608018684	0	0	0	0
unclassified(100)	Otu007760	0	0	0.000218150087260035	0	0
unclassified(100)	Otu007811	0.0000778513040093422	0	0	0	0
unclassified(100)	Otu007820	0.0000778513040093422	0	0	0	0
Dysgonomonas(100)	Otu007835	0	0.000124968757810547	0	0	0
Dysgonomonas(100)	Otu007838	0	0	0	0	0.000110387459984546
unclassified(100)	Otu007844	0	0	0	0	0.000110387459984546
unclassified(100)	Otu007847	0.0000778513040093422	0	0	0	0
unclassified(100)	Otu007853	0.0000778513040093422	0	0	0	0
Legionella(100)	Otu007859	0	0.000124968757810547	0	0	0
Streptococcus(100)	Otu007871	0	0	0	0.00012482836100362	0
unclassified(100)	Otu007909	0	0	0	0.00012482836100362	0
unclassified(100)	Otu007934	0	0	0	0	0.000110387459984546
unclassified(100)	Otu007941	0.0000778513040093422	0	0	0	0
unclassified(100)	Otu007952	0	0	0	0	0.000110387459984546
unclassified(100)	Otu007962	0.0000778513040093422	0	0	0	0
Chryseobacterium(100)	Otu007987	0	0.000124968757810547	0	0	0
unclassified(100)	Otu007999	0	0	0	0	0.000110387459984546
unclassified(100)	Otu008066	0	0.000999750062484379	0	0	0
Bdellovibrio(100)	Otu008091	0	0	0	0	0.000110387459984546
unclassified(100)	Otu008187	0	0	0	0.00012482836100362	0
Pseudomonas(100)	Otu008247	0	0	0	0.00012482836100362	0
unclassified(100)	Otu008259	0	0	0	0.0006241418050181	0
Nevskia(100)	Otu008296	0	0	0.000109075043630017	0	0
unclassified(100)	Otu008319	0	0	0.000109075043630017	0	0
Pseudomonas(100)	Otu008375	0	0	0	0.00012482836100362	0
Pseudomonas(100)	Otu008433	0	0	0	0.00012482836100362	0
unclassified(100)	Otu008479	0.0000778513040093422	0	0	0	0
Johnsonella(100)	Otu008486	0	0	0	0	0.000662324759907275
unclassified(100)	Otu008528	0.0000778513040093422	0	0	0	0

unclassified(100)	Otu008532	0	0	0	0.00099862688802896	0
unclassified(100)	Otu008534	0.0000778513040093422	0	0	0	0
unclassified(100)	Otu008683	0.0000778513040093422	0	0	0	0
unclassified	Otu008721	0	0	0	0	0.000110387459984546
unclassified(100)	Otu008722	0	0.000249937515621095	0	0	0
unclassified(100)	Otu008727	0	0	0	0	0.00132464951981455
unclassified(100)	Otu008737	0	0	0	0.00237173885906878	0.00342201125952092
Craurococcus(100)	Otu008755	0	0	0	0	0.000662324759907275
unclassified	Otu008760	0	0	0	0.00012482836100362	0
Gluconobacter(100)	Otu008778	0	0	0	0	0.000110387459984546
Craurococcus(100)	Otu008780	0	0	0	0.00012482836100362	0
Gluconobacter(100)	Otu008857	0	0	0	0	0.000110387459984546
unclassified(100)	Otu008950	0	0	0	0.00012482836100362	0
Pseudomonas(100)	Otu009235	0	0.000124968757810547	0	0	0
unclassified	Otu009382	0	0	0	0	0.000110387459984546
Pseudomonas(100)	Otu009405	0	0	0.00043630017452007	0	0
Pseudomonas(100)	Otu009438	0	0	0	0.00012482836100362	0
unclassified(100)	Otu009489	0	0.000124968757810547	0	0	0
unclassified(100)	Otu009507	0	0	0	0.00012482836100362	0
unclassified(100)	Otu009537	0	0	0	0	0.000110387459984546
unclassified(100)	Otu009544	0	0	0	0	0.000110387459984546
unclassified(100)	Otu009547	0	0	0	0.00012482836100362	0
Phaselicystis(100)	Otu009556	0	0	0.000109075043630017	0	0
unclassified(100)	Otu009569	0	0.000124968757810547	0	0	0
unclassified(100)	Otu009580	0	0	0	0	0.000110387459984546
unclassified(100)	Otu009582	0	0	0	0.00012482836100362	0
unclassified(100)	Otu009590	0	0.000124968757810547	0	0	0
unclassified(100)	Otu009608	0	0	0.000109075043630017	0	0
unclassified(100)	Otu009613	0	0.000124968757810547	0	0	0
unclassified(100)	Otu009622	0	0.000124968757810547	0.000109075043630017	0	0
unclassified(100)	Otu009655	0	0	0	0	0.000331162379953637
unclassified(100)	Otu009797	0	0	0	0.00112345524903258	0
unclassified(100)	Otu009798	0.0000778513040093422	0	0	0	0

unclassified(100)	Otu009800	0	0	0.000109075043630017	0	0
unclassified(100)	Otu009845	0.0000778513040093422	0	0	0	0
Pseudomonas(100)	Otu009887	0	0	0	0.00012482836100362	0
Pseudomonas(100)	Otu009891	0	0.000124968757810547	0	0	0
unclassified(100)	Otu009946	0.000155702608018684	0	0	0	0
Novosphingobium(100)	Otu009979	0.0000778513040093422	0	0	0	0
Prevotella(100)	Otu009991	0.0000778513040093422	0	0	0	0
Prevotella(100)	Otu009998	0	0	0.000109075043630017	0	0
unclassified(100)	Otu010008	0	0	0	0	0.000331162379953637
Chryseobacterium(100)	Otu010045	0	0	0	0.00012482836100362	0
Candidatus_Rhabdochlamydia(100)	Otu010056	0	0	0.000654450261780105	0	0
Flavobacterium(100)	Otu010061	0	0	0	0	0.000220774919969092
unclassified(100)	Otu010078	0	0	0	0	0.000110387459984546
unclassified(100)	Otu010084	0	0	0	0	0.000110387459984546
unclassified(100)	Otu010087	0	0	0	0	0.000110387459984546
unclassified	Otu010092	0	0	0.000109075043630017	0	0
Aquabacterium(100)	Otu010104	0	0	0	0	0.000110387459984546
unclassified(100)	Otu010105	0	0	0.000109075043630017	0	0
unclassified(100)	Otu010107	0	0	0	0	0.000110387459984546
Rhizobacter(100)	Otu010108	0	0	0.000109075043630017	0	0
Aquabacterium(100)	Otu010133	0	0	0.000109075043630017	0	0
unclassified	Otu010148	0	0	0	0	0.000110387459984546
unclassified	Otu010158	0.0000778513040093422	0	0	0	0
Novosphingobium(100)	Otu010160	0.0000778513040093422	0	0	0	0
unclassified(100)	Otu010169	0.0000778513040093422	0	0.000109075043630017	0	0
unclassified(100)	Otu010185	0.0000778513040093422	0	0	0	0
Aquabacterium(100)	Otu010187	0	0	0.000109075043630017	0	0
unclassified(100)	Otu010189	0	0.000124968757810547	0	0	0
unclassified	Otu010201	0.0000778513040093422	0	0	0	0
Flavobacterium(100)	Otu010203	0	0	0	0.0006241418050181	0
Marinobacter(100)	Otu010218	0	0	0	0	0.00132464951981455
Rheinheimera(100)	Otu010278	0	0	0	0.0006241418050181	0
unclassified(100)	Otu010300	0	0	0	0	0.000110387459984546

unclassified(100)	Otu010303	0	0	0.000218150087260035	0	0
unclassified(100)	Otu010355	0	0	0	0	0.000551937299922729
unclassified	Otu010401	0.0000778513040093422	0	0	0	0
unclassified(100)	Otu010402	0	0	0	0	0.000110387459984546
Flavobacterium(100)	Otu010410	0	0.000124968757810547	0	0	0
unclassified(100)	Otu010448	0	0	0	0	0.000110387459984546
unclassified	Otu010505	0	0	0	0	0.000110387459984546
unclassified(100)	Otu010509	0	0	0.000109075043630017	0	0
Aquabacterium(100)	Otu010518	0.0000778513040093422	0	0	0	0
unclassified(100)	Otu010558	0	0.000124968757810547	0	0	0
unclassified(100)	Otu010559	0	0	0	0	0.000110387459984546
Brevundimonas(100)	Otu010592	0	0	0	0	0.000110387459984546
Caulobacter(100)	Otu010603	0.0000778513040093422	0	0	0	0
Brevundimonas(100)	Otu010610	0	0	0	0	0.000110387459984546
unclassified(100)	Otu010611	0	0.000124968757810547	0	0	0
unclassified(100)	Otu010660	0.0000778513040093422	0	0	0	0
unclassified	Otu010684	0	0	0	0	0.000110387459984546
unclassified(100)	Otu010712	0	0	0.000109075043630017	0	0
Staphylococcus(100)	Otu010734	0	0.000124968757810547	0	0	0
unclassified	Otu010777	0	0	0	0	0.000110387459984546
Aquabacterium(100)	Otu010781	0	0	0	0	0.000110387459984546
Aquabacterium(100)	Otu010801	0	0	0.000109075043630017	0	0
unclassified(100)	Otu010815	0	0	0	0	0.000110387459984546
unclassified	Otu010818	0	0	0	0	0.000110387459984546
Aquabacterium(100)	Otu010824	0.0000778513040093422	0	0	0	0
unclassified(100)	Otu010832	0.0000778513040093422	0	0	0	0
Rhizobacter(100)	Otu010855	0.0000778513040093422	0	0	0	0
Aquabacterium(100)	Otu010868	0	0	0	0	0.000110387459984546
unclassified	Otu010871	0	0	0	0	0.000110387459984546
unclassified(100)	Otu010907	0	0	0.000109075043630017	0	0
Aquabacterium(100)	Otu010909	0	0	0.000109075043630017	0	0
unclassified(100)	Otu010914	0	0.000124968757810547	0	0	0.000110387459984546
unclassified(100)	Otu010915	0	0	0	0	0.000110387459984546

unclassified	Otu010917	0	0	0.000109075043630017	0	0
unclassified	Otu010920	0	0	0	0	0.000110387459984546
unclassified(100)	Otu010936	0	0.000124968757810547	0	0	0
unclassified(100)	Otu011028	0	0	0	0	0.000110387459984546
Acinetobacter(100)	Otu011072	0	0	0.000109075043630017	0	0
Acinetobacter(100)	Otu011073	0	0.000124968757810547	0	0	0
unclassified(100)	Otu011113	0	0	0	0.00074897016602172	0
Flavobacterium(100)	Otu011148	0	0	0.000109075043630017	0	0
unclassified(100)	Otu011173	0	0.000124968757810547	0	0	0
unclassified(100)	Otu011193	0.0000778513040093422	0	0	0	0
unclassified(100)	Otu011230	0	0	0.000109075043630017	0	0
unclassified(100)	Otu011233	0	0	0.000109075043630017	0	0
unclassified(100)	Otu011325	0	0	0.000654450261780105	0	0
unclassified(100)	Otu011361	0	0	0	0.00012482836100362	0
unclassified(100)	Otu011362	0	0	0	0.00012482836100362	0
Chryseobacterium(100)	Otu011440	0	0	0.000109075043630017	0	0
Methylobacterium(100)	Otu011451	0	0	0	0	0.000110387459984546
Methylobacterium(100)	Otu011459	0	0	0	0	0.000110387459984546
Chryseobacterium(100)	Otu011477	0	0	0	0	0.000110387459984546
Chryseobacterium(100)	Otu011516	0	0	0.000109075043630017	0	0
unclassified(100)	Otu011522	0	0.000124968757810547	0	0	0
Chryseobacterium(100)	Otu011527	0	0	0.000327225130890052	0	0
Bergeyella(100)	Otu011538	0	0	0	0.00012482836100362	0
Fluviicola(100)	Otu011600	0	0.000124968757810547	0	0	0
Chryseobacterium(100)	Otu011641	0	0	0.000109075043630017	0	0
unclassified	Otu011718	0	0.000124968757810547	0	0	0
Acinetobacter(100)	Otu011740	0	0.000124968757810547	0	0	0
Acinetobacter(100)	Otu011755	0	0	0	0	0.000110387459984546
Perlucidibaca(100)	Otu011773	0.000155702608018684	0	0	0	0
Rhizobium(100)	Otu011796	0	0	0	0.00012482836100362	0
Rhizobium(100)	Otu011811	0	0.000124968757810547	0	0	0
unclassified(100)	Otu011849	0	0	0	0.00012482836100362	0
unclassified(100)	Otu011850	0	0	0	0.00012482836100362	0

Aquicella(100)	Otu011891	0	0	0	0	0.000110387459984546
Dysgonomonas(100)	Otu013332	0	0.000124968757810547	0	0	0
Dysgonomonas(100)	Otu013358	0	0.000124968757810547	0	0	0
Dysgonomonas(100)	Otu013369	0	0.000124968757810547	0	0	0
Dysgonomonas(100)	Otu013370	0	0.000124968757810547	0	0	0
Dysgonomonas(100)	Otu014327	0	0.000124968757810547	0	0	0
Dysgonomonas(100)	Otu014437	0	0.000124968757810547	0	0	0
Dysgonomonas(100)	Otu014440	0	0.000124968757810547	0	0	0
Dysgonomonas(100)	Otu014441	0	0.000124968757810547	0	0	0
Dysgonomonas(100)	Otu014448	0.0000778513040093422	0	0	0	0
Dysgonomonas(100)	Otu014451	0	0.000124968757810547	0	0	0
Dysgonomonas(100)	Otu014461	0	0.000124968757810547	0	0	0
Dysgonomonas(100)	Otu014584	0	0.000124968757810547	0	0	0
Dysgonomonas(100)	Otu014942	0.0000778513040093422	0	0	0	0
Dysgonomonas(100)	Otu014968	0	0.000124968757810547	0	0	0
Dysgonomonas(100)	Otu015022	0	0.000124968757810547	0	0	0
Dysgonomonas(100)	Otu015025	0	0.000124968757810547	0	0	0
Porphyromonas(100)	Otu015044	0	0	0.000218150087260035	0	0
Dysgonomonas(100)	Otu015076	0	0.000124968757810547	0	0	0
Dysgonomonas(100)	Otu015082	0	0.000124968757810547	0	0	0
Dysgonomonas(100)	Otu015086	0	0.000124968757810547	0	0	0
Dysgonomonas(100)	Otu015092	0	0.000124968757810547	0	0	0
Dysgonomonas(100)	Otu015131	0	0.000124968757810547	0	0	0
Dysgonomonas(100)	Otu015138	0	0.000124968757810547	0	0	0
Dysgonomonas(100)	Otu015169	0	0.000124968757810547	0	0	0
Dysgonomonas(100)	Otu015177	0	0.000124968757810547	0	0	0
Dysgonomonas(100)	Otu015190	0	0.000124968757810547	0	0	0
Dysgonomonas(100)	Otu015205	0	0.000124968757810547	0	0	0
Dysgonomonas(100)	Otu015215	0	0.000124968757810547	0	0	0
Dysgonomonas(100)	Otu015241	0	0.000124968757810547	0	0	0
Dysgonomonas(100)	Otu015265	0	0.000124968757810547	0	0	0
Dysgonomonas(100)	Otu015269	0	0.000124968757810547	0	0	0
Dysgonomonas(100)	Otu015279	0	0.000124968757810547	0	0	0

Dysgonomonas(100)	Otu015280	0	0.000124968757810547	0	0	0
Dysgonomonas(100)	Otu015281	0	0.000124968757810547	0	0	0
Dysgonomonas(100)	Otu015284	0	0.000124968757810547	0	0	0
Dysgonomonas(100)	Otu015288	0	0.000124968757810547	0	0	0
Dysgonomonas(100)	Otu015320	0	0.000124968757810547	0	0	0
Dysgonomonas(100)	Otu015329	0	0.000124968757810547	0	0	0
Dysgonomonas(100)	Otu015333	0	0.000124968757810547	0	0	0
Dysgonomonas(100)	Otu015362	0	0.000124968757810547	0	0	0
Dysgonomonas(100)	Otu015378	0	0.000124968757810547	0	0	0
Dysgonomonas(100)	Otu015379	0	0.000124968757810547	0	0	0
Dysgonomonas(100)	Otu015390	0	0.000124968757810547	0	0	0
Dysgonomonas(100)	Otu015393	0	0.000124968757810547	0	0	0
Dysgonomonas(100)	Otu015403	0	0.000124968757810547	0	0	0
Dysgonomonas(100)	Otu015411	0	0.000124968757810547	0	0	0
Dysgonomonas(100)	Otu015420	0	0.000124968757810547	0	0	0
Dysgonomonas(100)	Otu015461	0	0.000124968757810547	0	0	0
Dysgonomonas(100)	Otu015464	0	0.000124968757810547	0	0	0
Dysgonomonas(100)	Otu015468	0	0.000124968757810547	0	0	0
Dysgonomonas(100)	Otu015483	0	0.000124968757810547	0	0	0
Dysgonomonas(100)	Otu015490	0	0.000124968757810547	0	0	0
Dysgonomonas(100)	Otu015501	0	0.000124968757810547	0	0	0
Dysgonomonas(100)	Otu015511	0	0.000124968757810547	0	0	0
Dysgonomonas(100)	Otu015525	0	0.000124968757810547	0	0	0
Dysgonomonas(100)	Otu015532	0	0.000124968757810547	0	0	0
Dysgonomonas(100)	Otu015545	0	0.000124968757810547	0	0	0
Dysgonomonas(100)	Otu015546	0	0.000124968757810547	0	0	0
Dysgonomonas(100)	Otu015577	0	0.000124968757810547	0	0	0
Dysgonomonas(100)	Otu015579	0	0.000124968757810547	0	0	0
Dysgonomonas(100)	Otu015592	0	0.000124968757810547	0	0	0
Dysgonomonas(100)	Otu015594	0	0.000124968757810547	0	0	0
Dysgonomonas(100)	Otu015595	0	0.000124968757810547	0	0	0
Dysgonomonas(100)	Otu015606	0	0.000124968757810547	0	0	0
Dysgonomonas(100)	Otu015608	0	0.000124968757810547	0	0	0

Dysgonomonas(100)	Otu015611	0	0.000124968757810547	0	0	0
Dysgonomonas(100)	Otu015615	0	0.000124968757810547	0	0	0
Dysgonomonas(100)	Otu015617	0	0.000124968757810547	0	0	0
Dysgonomonas(100)	Otu015620	0	0.000124968757810547	0	0	0
Dysgonomonas(100)	Otu015628	0	0.000124968757810547	0	0	0
Dysgonomonas(100)	Otu015631	0	0.000124968757810547	0	0	0
Dysgonomonas(100)	Otu015640	0	0.000124968757810547	0	0	0
Dysgonomonas(100)	Otu015642	0	0.000124968757810547	0	0	0
Dysgonomonas(100)	Otu015650	0	0.000124968757810547	0	0	0
Dysgonomonas(100)	Otu015657	0	0.000124968757810547	0	0	0
unclassified(100)	Otu015664	0	0.000124968757810547	0	0	0
Dysgonomonas(100)	Otu015673	0	0.000124968757810547	0	0	0
Dysgonomonas(100)	Otu015717	0	0.000124968757810547	0	0	0
Dysgonomonas(100)	Otu015724	0	0.000124968757810547	0	0	0
Dysgonomonas(100)	Otu015744	0	0.000124968757810547	0	0	0
Dysgonomonas(100)	Otu015749	0	0.000124968757810547	0	0	0
Dysgonomonas(100)	Otu015764	0	0.000124968757810547	0	0	0
Dysgonomonas(100)	Otu015765	0	0.000124968757810547	0	0	0
Dysgonomonas(100)	Otu015767	0	0.000124968757810547	0	0	0
Dysgonomonas(100)	Otu015782	0	0.000124968757810547	0	0	0
unclassified(100)	Otu016330	0	0	0	0	0.000110387459984546
unclassified(100)	Otu016335	0	0	0	0	0.000110387459984546
unclassified(100)	Otu016337	0	0	0	0	0.000110387459984546
unclassified(100)	Otu016339	0	0	0	0	0.000110387459984546
unclassified(100)	Otu016341	0	0	0	0	0.000110387459984546
unclassified(100)	Otu016344	0	0	0	0	0.000110387459984546
unclassified(100)	Otu016347	0	0	0	0	0.000110387459984546
unclassified(100)	Otu016350	0	0	0	0	0.000110387459984546
unclassified(100)	Otu016356	0	0	0	0	0.000110387459984546
unclassified(100)	Otu016359	0	0	0	0	0.000110387459984546
unclassified(100)	Otu016389	0	0	0	0	0.000110387459984546
unclassified(100)	Otu016391	0	0	0	0	0.000110387459984546
unclassified(100)	Otu016398	0	0	0	0	0.000110387459984546

unclassified(100)	Otu016399	0	0	0	0	0.000110387459984546
Aquabacterium(100)	Otu016408	0	0	0	0	0.000110387459984546
Aquabacterium(100)	Otu016409	0	0	0	0	0.000110387459984546
unclassified(100)	Otu016423	0	0	0	0	0.000110387459984546
unclassified(100)	Otu016424	0	0	0	0	0.000110387459984546
unclassified(100)	Otu016436	0	0	0	0	0.000110387459984546
unclassified(100)	Otu016438	0	0	0	0	0.000110387459984546
unclassified(100)	Otu016440	0	0	0	0	0.000110387459984546
unclassified(100)	Otu016441	0	0	0	0	0.000110387459984546
Aquabacterium(100)	Otu016442	0	0	0	0	0.000110387459984546
Rhizobacter(100)	Otu016474	0	0	0	0	0.000110387459984546
unclassified(100)	Otu016501	0	0	0	0	0.000110387459984546
unclassified(100)	Otu016505	0	0	0	0	0.000110387459984546
unclassified(100)	Otu016508	0	0	0	0	0.000110387459984546
unclassified(100)	Otu016529	0	0	0	0	0.000110387459984546
Aquabacterium(100)	Otu016531	0	0	0	0	0.000110387459984546
unclassified(100)	Otu016534	0	0	0	0	0.000110387459984546
unclassified(100)	Otu016538	0	0	0	0	0.000110387459984546
Rhizobacter(100)	Otu016539	0	0	0	0	0.000110387459984546
unclassified(100)	Otu016545	0	0	0	0	0.000110387459984546
Aquabacterium(100)	Otu016546	0	0	0	0	0.000110387459984546
unclassified(100)	Otu016620	0	0	0	0	0.000110387459984546
unclassified(100)	Otu016622	0	0	0	0	0.000110387459984546
unclassified(100)	Otu016627	0	0	0	0	0.000110387459984546
unclassified(100)	Otu016628	0	0	0	0	0.000110387459984546
unclassified(100)	Otu016636	0	0	0	0	0.000110387459984546
unclassified(100)	Otu016675	0	0	0	0	0.000110387459984546
Tepidimonas(100)	Otu016680	0	0	0	0	0.000110387459984546
unclassified(100)	Otu016681	0	0	0	0	0.000110387459984546
unclassified(100)	Otu016682	0	0	0	0	0.000110387459984546
unclassified(100)	Otu016687	0	0	0	0	0.000110387459984546
Rhizobacter(100)	Otu016689	0	0	0	0	0.000110387459984546
Aquabacterium(100)	Otu016694	0	0	0	0	0.000110387459984546

unclassified(100)	Otu016702	0	0	0	0	0.000110387459984546
Aquabacterium(100)	Otu016709	0	0	0	0	0.000110387459984546
unclassified(100)	Otu016711	0	0	0	0	0.000110387459984546
Aquabacterium(100)	Otu016713	0	0	0	0	0.000110387459984546
unclassified(100)	Otu016714	0	0	0	0	0.000110387459984546
Aquabacterium(100)	Otu016719	0	0	0	0	0.000110387459984546
unclassified(100)	Otu016720	0	0	0	0	0.000110387459984546
Aquabacterium(100)	Otu016721	0	0	0	0	0.000110387459984546
unclassified(100)	Otu016724	0	0	0	0	0.000110387459984546
Aquabacterium(100)	Otu016725	0	0	0	0	0.000110387459984546
unclassified(100)	Otu016727	0	0	0	0	0.000110387459984546
Aquabacterium(100)	Otu016728	0	0	0	0	0.000110387459984546
unclassified(100)	Otu016763	0	0	0	0	0.000110387459984546
unclassified(100)	Otu016765	0	0	0	0	0.000110387459984546
Aquabacterium(100)	Otu016767	0	0	0	0	0.000110387459984546
Rhizobacter(100)	Otu016776	0	0	0	0	0.000110387459984546
unclassified(100)	Otu016777	0	0	0	0	0.000110387459984546
unclassified(100)	Otu016782	0	0	0	0	0.000110387459984546
unclassified(100)	Otu016855	0	0	0	0	0.000110387459984546
unclassified(100)	Otu016857	0	0	0	0	0.000110387459984546
unclassified(100)	Otu016858	0	0	0	0	0.000110387459984546
unclassified(100)	Otu016859	0	0	0	0	0.000110387459984546
unclassified(100)	Otu016860	0	0	0	0	0.000110387459984546
unclassified(100)	Otu016861	0	0	0	0	0.000110387459984546
unclassified(100)	Otu016862	0	0	0	0	0.000110387459984546
unclassified(100)	Otu016864	0	0	0	0	0.000110387459984546
unclassified(100)	Otu016866	0	0	0	0	0.000110387459984546
unclassified(100)	Otu016905	0	0	0	0	0.000110387459984546
unclassified(100)	Otu016916	0	0	0	0	0.000110387459984546
unclassified(100)	Otu016917	0	0	0	0	0.000110387459984546
unclassified(100)	Otu016920	0	0	0	0	0.000110387459984546
Aquabacterium(100)	Otu016947	0	0	0	0	0.000110387459984546
Dysgonomonas(100)	Otu016967	0	0	0	0.00012482836100362	0

Dysgonomonas(100)	Otu016971	0	0	0	0.00012482836100362	0
Dysgonomonas(100)	Otu016981	0	0	0	0.00012482836100362	0
unclassified(100)	Otu017018	0	0	0	0.00012482836100362	0
unclassified(100)	Otu017483	0	0	0	0	0.000110387459984546
unclassified(100)	Otu017518	0	0	0	0	0.000110387459984546
unclassified(100)	Otu017720	0	0.000124968757810547	0	0	0
Aquabacterium(100)	Otu017723	0	0.000124968757810547	0	0	0
unclassified(100)	Otu017733	0	0.000124968757810547	0	0	0
unclassified(100)	Otu017783	0	0	0.000109075043630017	0	0
Aquabacterium(100)	Otu017791	0	0.000124968757810547	0	0	0
unclassified(100)	Otu017798	0	0.000124968757810547	0	0	0
unclassified(100)	Otu017815	0	0.000124968757810547	0	0	0
unclassified(100)	Otu017832	0	0.000124968757810547	0	0	0
Tepidimonas(100)	Otu017844	0	0.000124968757810547	0	0	0
unclassified(100)	Otu017888	0	0.000124968757810547	0	0	0
unclassified(100)	Otu017900	0	0.000124968757810547	0	0	0
unclassified(100)	Otu017903	0	0.000124968757810547	0	0	0
unclassified(100)	Otu017906	0	0.000124968757810547	0	0	0
unclassified(100)	Otu017908	0	0.000124968757810547	0	0	0
unclassified(100)	Otu017934	0	0.000124968757810547	0	0	0
unclassified(100)	Otu017942	0	0.000124968757810547	0	0	0
unclassified(100)	Otu017947	0	0	0	0.00012482836100362	0
unclassified(100)	Otu017958	0	0	0	0.00012482836100362	0
unclassified(100)	Otu017960	0	0	0	0.00012482836100362	0
unclassified(100)	Otu017970	0	0	0	0.00012482836100362	0
unclassified(100)	Otu017971	0	0	0	0.00012482836100362	0
unclassified(100)	Otu017980	0	0.000124968757810547	0	0	0
Aquabacterium(100)	Otu017984	0	0.000124968757810547	0	0	0
Aquabacterium(100)	Otu017991	0	0.000124968757810547	0	0	0
unclassified(100)	Otu017995	0	0.000124968757810547	0	0	0
unclassified(100)	Otu018012	0	0	0.000109075043630017	0	0
unclassified(100)	Otu018014	0	0	0.000109075043630017	0	0
Aquabacterium(100)	Otu018021	0	0	0.000109075043630017	0	0

Aquabacterium(100)	Otu018023	0	0	0.000109075043630017	0	0
unclassified(100)	Otu018038	0	0.000124968757810547	0	0	0
unclassified(100)	Otu018060	0	0.000124968757810547	0	0	0
unclassified(100)	Otu018061	0	0.000124968757810547	0	0	0
unclassified(100)	Otu018069	0	0.000124968757810547	0	0	0
unclassified(100)	Otu018082	0	0.000124968757810547	0	0	0
Aquabacterium(100)	Otu018083	0	0.000124968757810547	0	0	0
unclassified(100)	Otu018091	0	0	0.000109075043630017	0	0
Tepidimonas(100)	Otu018098	0	0.000124968757810547	0	0	0
Tepidimonas(100)	Otu018100	0	0.000124968757810547	0	0	0
Aquabacterium(100)	Otu018105	0	0.000124968757810547	0	0	0
unclassified(100)	Otu018115	0	0.000124968757810547	0	0	0
unclassified(100)	Otu018121	0	0	0.000109075043630017	0	0
unclassified(100)	Otu018131	0	0	0.000109075043630017	0	0
Aquabacterium(100)	Otu018132	0	0	0.000109075043630017	0	0
unclassified(100)	Otu018136	0	0	0.000109075043630017	0	0
Aquabacterium(100)	Otu018137	0	0	0.000109075043630017	0	0
Aquabacterium(100)	Otu018139	0	0	0.000109075043630017	0	0
unclassified(100)	Otu018145	0	0.000124968757810547	0	0	0
unclassified(100)	Otu018148	0	0	0.000109075043630017	0	0
unclassified(100)	Otu018149	0	0	0.000109075043630017	0	0
unclassified(100)	Otu018151	0	0	0.000109075043630017	0	0
unclassified(100)	Otu018153	0	0	0.000109075043630017	0	0
Aquabacterium(100)	Otu018156	0	0	0.000109075043630017	0	0
unclassified(100)	Otu018158	0	0	0.000109075043630017	0	0
Aquabacterium(100)	Otu018163	0	0	0.000109075043630017	0	0
unclassified(100)	Otu018164	0	0	0.000109075043630017	0	0
unclassified(100)	Otu018167	0	0	0.000109075043630017	0	0
unclassified(100)	Otu018171	0	0	0.000109075043630017	0	0
Aquabacterium(100)	Otu018172	0	0	0.000109075043630017	0	0
Aquabacterium(100)	Otu018180	0	0	0.000109075043630017	0	0
Aquabacterium(100)	Otu018190	0	0	0.000109075043630017	0	0
Aquabacterium(100)	Otu018207	0	0	0.000109075043630017	0	0

Aquabacterium(100)	Otu018225	0	0	0.000109075043630017	0	0
unclassified(100)	Otu018227	0	0	0.000109075043630017	0	0
Aquabacterium(100)	Otu018228	0	0	0.000109075043630017	0	0
unclassified(100)	Otu018235	0	0.000124968757810547	0	0	0
unclassified(100)	Otu018238	0	0.000124968757810547	0	0	0
unclassified(100)	Otu018251	0	0.000124968757810547	0	0	0
unclassified(100)	Otu018258	0	0	0.000109075043630017	0	0
unclassified(100)	Otu018261	0	0	0.000109075043630017	0	0
unclassified(100)	Otu018269	0	0.000124968757810547	0	0	0
unclassified(100)	Otu018272	0.0000778513040093422	0	0	0	0
unclassified(100)	Otu018273	0	0	0.000109075043630017	0	0
unclassified(100)	Otu018275	0	0	0.000109075043630017	0	0
unclassified(100)	Otu018279	0	0	0.000109075043630017	0	0
unclassified(100)	Otu018282	0	0	0.000109075043630017	0	0
unclassified(100)	Otu018287	0	0.000124968757810547	0	0	0
Aquabacterium(100)	Otu018312	0	0	0.000109075043630017	0	0
unclassified(100)	Otu018315	0	0	0.000109075043630017	0	0
Rhizobacter(100)	Otu018319	0	0	0.000109075043630017	0	0
Aquabacterium(100)	Otu018320	0.0000778513040093422	0	0	0	0
unclassified(100)	Otu018325	0	0	0.000109075043630017	0	0
unclassified(100)	Otu018326	0.0000778513040093422	0	0	0	0
Aquabacterium(100)	Otu018342	0	0	0.000109075043630017	0	0
unclassified(100)	Otu018343	0	0	0.000109075043630017	0	0
unclassified(100)	Otu018345	0	0	0.000109075043630017	0	0
unclassified(100)	Otu018348	0.0000778513040093422	0	0	0	0
unclassified(100)	Otu018355	0.0000778513040093422	0	0	0	0
unclassified(100)	Otu018370	0.0000778513040093422	0	0	0	0
unclassified(100)	Otu018371	0.0000778513040093422	0	0	0	0
unclassified(100)	Otu018372	0.0000778513040093422	0	0	0	0
unclassified(100)	Otu018387	0.0000778513040093422	0	0	0	0
unclassified(100)	Otu018388	0.0000778513040093422	0	0	0	0
Aquabacterium(100)	Otu018393	0.0000778513040093422	0	0	0	0
unclassified(100)	Otu018410	0.0000778513040093422	0	0	0	0

unclassified(100)	Otu018436	0.0000778513040093422	0	0	0	0
unclassified(100)	Otu018443	0.0000778513040093422	0	0	0	0
Aquabacterium(100)	Otu018449	0.0000778513040093422	0	0	0	0
unclassified(100)	Otu018452	0.0000778513040093422	0	0	0	0
unclassified(100)	Otu018462	0.0000778513040093422	0	0	0	0
Aquabacterium(100)	Otu018463	0.0000778513040093422	0	0	0	0
unclassified(100)	Otu018464	0.0000778513040093422	0	0	0	0
unclassified(100)	Otu018465	0.0000778513040093422	0	0	0	0
unclassified(100)	Otu018466	0.0000778513040093422	0	0	0	0
unclassified(100)	Otu018468	0.0000778513040093422	0	0	0	0
unclassified(100)	Otu018470	0.0000778513040093422	0	0	0	0
unclassified(100)	Otu018471	0.0000778513040093422	0	0	0	0
unclassified(100)	Otu018475	0.0000778513040093422	0	0	0	0
unclassified(100)	Otu018479	0.0000778513040093422	0	0	0	0
Aquabacterium(100)	Otu018482	0.0000778513040093422	0	0	0	0
unclassified(100)	Otu018492	0.0000778513040093422	0	0	0	0
unclassified(100)	Otu018497	0.0000778513040093422	0	0	0	0
unclassified(100)	Otu018504	0.0000778513040093422	0	0	0	0
unclassified(100)	Otu018521	0.0000778513040093422	0	0	0	0
unclassified(100)	Otu018524	0.0000778513040093422	0	0	0	0
unclassified(100)	Otu018528	0.0000778513040093422	0	0	0	0
unclassified(100)	Otu018539	0.0000778513040093422	0	0	0	0
unclassified(100)	Otu018540	0.0000778513040093422	0	0	0	0
unclassified(100)	Otu018541	0.0000778513040093422	0	0	0	0
unclassified(100)	Otu018544	0.0000778513040093422	0	0	0	0
Aquabacterium(100)	Otu018548	0.0000778513040093422	0	0	0	0
unclassified(100)	Otu018549	0.0000778513040093422	0	0	0	0
unclassified(100)	Otu018551	0.0000778513040093422	0	0	0	0
Tepidimonas(100)	Otu018567	0.0000778513040093422	0	0	0	0
unclassified(100)	Otu018601	0	0.000124968757810547	0	0	0
unclassified(100)	Otu018603	0	0.000124968757810547	0	0	0
unclassified(100)	Otu018608	0.0000778513040093422	0	0	0	0
unclassified(100)	Otu018614	0.0000778513040093422	0	0	0	0

unclassified(100)	Otu018621	0.0000778513040093422	0	0	0	0
unclassified(100)	Otu018630	0.0000778513040093422	0	0	0	0
Aquabacterium(100)	Otu018637	0	0	0	0.00012482836100362	0
unclassified(100)	Otu018641	0	0	0.000109075043630017	0	0
unclassified(100)	Otu018657	0	0	0.000109075043630017	0	0
unclassified(100)	Otu018663	0	0	0.000109075043630017	0	0
unclassified(100)	Otu018676	0.0000778513040093422	0	0	0	0
unclassified(100)	Otu018682	0	0	0.000109075043630017	0	0
unclassified(100)	Otu018689	0	0	0.000109075043630017	0	0
unclassified(100)	Otu018691	0	0	0.000109075043630017	0	0
unclassified(100)	Otu018703	0.0000778513040093422	0	0	0	0
unclassified(100)	Otu018716	0.0000778513040093422	0	0	0	0
unclassified(100)	Otu018718	0.0000778513040093422	0	0	0	0
Rhizobacter(100)	Otu018722	0.0000778513040093422	0	0	0	0
Aquabacterium(100)	Otu018723	0.0000778513040093422	0	0	0	0
unclassified(100)	Otu018730	0.0000778513040093422	0	0	0	0
unclassified(100)	Otu018732	0.0000778513040093422	0	0	0	0
Aquabacterium(100)	Otu018734	0.0000778513040093422	0	0	0	0
unclassified(100)	Otu018748	0.0000778513040093422	0	0	0	0
unclassified(100)	Otu018760	0.0000778513040093422	0	0	0	0
Rhizobacter(100)	Otu018768	0.0000778513040093422	0	0	0	0
Aquabacterium(100)	Otu018769	0.0000778513040093422	0	0	0	0
unclassified(100)	Otu018772	0.0000778513040093422	0	0	0	0
Aquabacterium(100)	Otu018774	0.0000778513040093422	0	0	0	0
unclassified(100)	Otu018779	0.0000778513040093422	0	0	0	0
unclassified(100)	Otu018792	0.0000778513040093422	0	0	0	0
unclassified(100)	Otu018797	0.0000778513040093422	0	0	0	0
unclassified(100)	Otu018799	0.0000778513040093422	0	0	0	0
unclassified(100)	Otu018805	0.0000778513040093422	0	0	0	0
unclassified(100)	Otu018807	0.0000778513040093422	0	0	0	0
unclassified(100)	Otu018812	0	0	0	0.00012482836100362	0
Tepidimonas(100)	Otu018813	0	0	0	0.00012482836100362	0
unclassified(100)	Otu018831	0	0	0	0.00012482836100362	0

unclassified(100)	Otu018852	0	0	0	0.00012482836100362	0
unclassified(100)	Otu018854	0	0	0	0.00012482836100362	0
unclassified(100)	Otu018855	0	0	0	0.00012482836100362	0
Aquabacterium(100)	Otu018866	0	0	0	0.00012482836100362	0
Aquabacterium(100)	Otu018871	0	0	0	0	0.000110387459984546
Aquabacterium(100)	Otu018872	0	0	0	0	0.000110387459984546
unclassified(100)	Otu018875	0	0	0	0	0.000110387459984546
unclassified(100)	Otu018894	0	0	0	0	0.000110387459984546
Aquabacterium(100)	Otu018898	0	0	0	0.00012482836100362	0
Aquabacterium(100)	Otu018904	0	0	0	0	0.000110387459984546
unclassified(100)	Otu018911	0	0	0	0	0.000110387459984546
unclassified(100)	Otu018920	0	0	0	0	0.000110387459984546
Rhizobacter(100)	Otu018923	0	0	0	0	0.000110387459984546
unclassified(100)	Otu018927	0	0	0.000109075043630017	0	0
unclassified(100)	Otu018931	0	0	0.000109075043630017	0	0
unclassified(100)	Otu018943	0	0	0.000109075043630017	0	0
unclassified(100)	Otu018962	0	0	0.000109075043630017	0	0
Aquabacterium(100)	Otu018969	0	0	0.000109075043630017	0	0
unclassified(100)	Otu018975	0	0	0.000109075043630017	0	0
unclassified(100)	Otu018976	0	0	0.000109075043630017	0	0
Aquabacterium(100)	Otu018979	0	0	0.000109075043630017	0	0
unclassified(100)	Otu018986	0	0	0.000109075043630017	0	0
unclassified(100)	Otu018987	0	0	0.000109075043630017	0	0
unclassified(100)	Otu018989	0	0	0.000109075043630017	0	0
unclassified(100)	Otu019002	0	0	0	0.00012482836100362	0
unclassified(100)	Otu019003	0	0	0	0.00012482836100362	0
Tepidimonas(100)	Otu019013	0	0	0	0.00012482836100362	0
unclassified(100)	Otu019019	0	0	0.000109075043630017	0	0
Aquabacterium(100)	Otu019035	0	0	0.000109075043630017	0	0
unclassified(100)	Otu019036	0	0	0.000109075043630017	0	0
unclassified(100)	Otu019047	0	0	0	0	0.000110387459984546
unclassified(100)	Otu019054	0	0	0	0	0.000110387459984546
unclassified(100)	Otu019056	0	0	0	0	0.000110387459984546

unclassified(100)	Otu019062	0	0	0	0	0.000110387459984546
unclassified(100)	Otu019070	0	0	0	0	0.000110387459984546
unclassified(100)	Otu019074	0	0	0	0	0.000110387459984546
unclassified(100)	Otu019100	0	0	0.000109075043630017	0	0
unclassified(100)	Otu019102	0	0	0.000109075043630017	0	0
unclassified(100)	Otu019110	0	0	0.000109075043630017	0	0
unclassified(100)	Otu019112	0	0	0.000109075043630017	0	0
unclassified(100)	Otu019124	0	0	0.000109075043630017	0	0
Aquabacterium(100)	Otu019128	0	0	0	0	0.000110387459984546
unclassified(100)	Otu019129	0	0	0	0	0.000110387459984546
unclassified(100)	Otu019134	0	0	0	0	0.000110387459984546
unclassified(100)	Otu019135	0	0	0	0	0.000110387459984546
Aquabacterium(100)	Otu019143	0	0	0.000109075043630017	0	0
Aquabacterium(100)	Otu019148	0	0	0	0	0.000110387459984546
unclassified(100)	Otu019151	0	0	0.000109075043630017	0	0
Aquabacterium(100)	Otu019153	0	0	0.000109075043630017	0	0
unclassified(100)	Otu019154	0	0	0.000109075043630017	0	0
Aquabacterium(100)	Otu019156	0	0	0.000109075043630017	0	0
unclassified(100)	Otu019166	0	0	0	0.00012482836100362	0
Aquabacterium(100)	Otu019189	0	0	0	0.00012482836100362	0
Aquabacterium(100)	Otu019202	0	0	0	0.00012482836100362	0
unclassified(100)	Otu019208	0	0	0	0.00012482836100362	0
unclassified(100)	Otu019218	0	0	0	0	0.000110387459984546
unclassified(100)	Otu019220	0	0	0	0	0.000110387459984546
unclassified(100)	Otu019222	0	0	0	0	0.000110387459984546
unclassified(100)	Otu019226	0	0	0	0	0.000110387459984546
unclassified(100)	Otu019231	0	0	0	0	0.000110387459984546
unclassified(100)	Otu019234	0	0	0	0	0.000110387459984546
unclassified(100)	Otu019235	0	0	0	0	0.000110387459984546
unclassified(100)	Otu019241	0	0	0	0	0.000110387459984546
unclassified(100)	Otu019250	0	0	0	0	0.000110387459984546
unclassified(100)	Otu019251	0	0	0	0	0.000110387459984546
Rhizobacter(100)	Otu019260	0	0	0	0	0.000110387459984546

unclassified(100)	Otu019261	0	0	0	0	0.000110387459984546
unclassified(100)	Otu019270	0	0	0	0	0.000110387459984546
unclassified(100)	Otu019276	0	0	0.000109075043630017	0	0
unclassified(100)	Otu019279	0	0	0.000109075043630017	0	0
unclassified(100)	Otu019283	0	0	0.000109075043630017	0	0
Aquabacterium(100)	Otu019284	0	0	0.000109075043630017	0	0
unclassified(100)	Otu019286	0	0	0.000109075043630017	0	0
unclassified(100)	Otu019292	0	0	0.000109075043630017	0	0
Rhizobacter(100)	Otu019304	0	0	0.000109075043630017	0	0
Aquabacterium(100)	Otu019305	0	0	0.000109075043630017	0	0
unclassified(100)	Otu019310	0	0	0.000109075043630017	0	0
unclassified(100)	Otu019311	0	0	0.000109075043630017	0	0
Rhizobacter(100)	Otu019325	0	0	0.000109075043630017	0	0
unclassified(100)	Otu019329	0	0	0.000109075043630017	0	0
unclassified(100)	Otu019337	0.0000778513040093422	0	0	0	0
unclassified(100)	Otu019339	0.0000778513040093422	0	0	0	0
unclassified(100)	Otu019340	0.0000778513040093422	0	0	0	0
unclassified(100)	Otu019343	0.0000778513040093422	0	0	0	0
unclassified(100)	Otu019346	0.0000778513040093422	0	0	0	0
unclassified(100)	Otu019354	0.0000778513040093422	0	0	0	0
Aquabacterium(100)	Otu019356	0.0000778513040093422	0	0	0	0
unclassified(100)	Otu019368	0	0	0.000109075043630017	0	0
unclassified(100)	Otu019369	0	0	0.000109075043630017	0	0
unclassified(100)	Otu019378	0.0000778513040093422	0	0	0	0
Aquabacterium(100)	Otu019381	0.0000778513040093422	0	0	0	0
unclassified(100)	Otu019383	0.0000778513040093422	0	0	0	0
unclassified(100)	Otu019385	0.0000778513040093422	0	0	0	0
unclassified(100)	Otu019388	0	0	0.000109075043630017	0	0
unclassified(100)	Otu019392	0	0	0.000109075043630017	0	0
unclassified(100)	Otu019394	0	0	0.000109075043630017	0	0
unclassified(100)	Otu019398	0	0	0.000109075043630017	0	0
unclassified(100)	Otu019405	0	0	0.000109075043630017	0	0
Aquabacterium(100)	Otu019412	0	0	0.000109075043630017	0	0

unclassified(100)	Otu019417	0	0	0.000109075043630017	0	0
unclassified(100)	Otu019421	0	0	0.000109075043630017	0	0
unclassified(100)	Otu019430	0	0	0.000109075043630017	0	0
unclassified(100)	Otu019432	0	0	0.000109075043630017	0	0
unclassified(100)	Otu019433	0	0	0.000109075043630017	0	0
Rhizobacter(100)	Otu019434	0	0	0.000109075043630017	0	0
unclassified(100)	Otu019436	0	0	0.000109075043630017	0	0
Pelomonas(100)	Otu019437	0	0	0.000109075043630017	0	0
unclassified(100)	Otu019438	0	0	0.000109075043630017	0	0
unclassified(100)	Otu019441	0	0	0.000109075043630017	0	0
unclassified(100)	Otu019442	0	0	0.000109075043630017	0	0
unclassified(100)	Otu019451	0	0	0.000109075043630017	0	0
unclassified(100)	Otu019454	0	0	0.000109075043630017	0	0
Aquabacterium(100)	Otu019456	0	0	0.000109075043630017	0	0
Aquabacterium(100)	Otu019457	0	0	0.000109075043630017	0	0
unclassified(100)	Otu019460	0	0	0.000109075043630017	0	0
unclassified(100)	Otu019467	0	0	0.000109075043630017	0	0
unclassified(100)	Otu019474	0	0	0.000109075043630017	0	0
unclassified(100)	Otu019475	0	0	0.000109075043630017	0	0
unclassified(100)	Otu019476	0	0	0.000109075043630017	0	0
Aquabacterium(100)	Otu019477	0	0	0.000109075043630017	0	0
unclassified(100)	Otu019478	0	0	0.000109075043630017	0	0
Aquabacterium(100)	Otu019480	0	0	0.000109075043630017	0	0
unclassified(100)	Otu019491	0	0	0.000109075043630017	0	0
unclassified(100)	Otu019496	0	0	0.000109075043630017	0	0
unclassified(100)	Otu019498	0	0	0.000109075043630017	0	0
unclassified(100)	Otu019499	0	0	0.000109075043630017	0	0
Dysgonomonas(100)	Otu019505	0	0	0	0	0.000110387459984546
unclassified(100)	Otu019511	0	0	0	0	0.000110387459984546
Dysgonomonas(100)	Otu019516	0	0	0	0	0.000110387459984546
Dysgonomonas(100)	Otu019520	0	0	0	0	0.000110387459984546
Dysgonomonas(100)	Otu019523	0	0	0	0	0.000110387459984546
Dysgonomonas(100)	Otu019531	0	0	0	0.00012482836100362	0

Dysgonomonas(100)	Otu019537	0	0	0	0.00012482836100362	0
Dysgonomonas(100)	Otu019539	0	0	0	0.00012482836100362	0
Dysgonomonas(100)	Otu019547	0	0	0	0.00012482836100362	0
Dysgonomonas(100)	Otu019550	0	0	0	0.00012482836100362	0
Dysgonomonas(100)	Otu019551	0	0	0	0	0.000110387459984546
Dysgonomonas(100)	Otu019552	0	0	0	0	0.000110387459984546
Dysgonomonas(100)	Otu019554	0	0	0	0	0.000110387459984546
Dysgonomonas(100)	Otu019557	0	0	0	0	0.000110387459984546
Dysgonomonas(100)	Otu019559	0	0	0	0	0.000110387459984546
Dysgonomonas(100)	Otu019567	0	0	0	0	0.000110387459984546
Dysgonomonas(100)	Otu019572	0	0	0	0	0.000110387459984546
Dysgonomonas(100)	Otu019573	0	0	0	0	0.000110387459984546
Dysgonomonas(100)	Otu019576	0	0	0	0	0.000110387459984546
Dysgonomonas(100)	Otu019578	0	0	0	0	0.000110387459984546
Dysgonomonas(100)	Otu019583	0	0	0	0	0.000110387459984546
Dysgonomonas(100)	Otu019584	0	0	0	0	0.000110387459984546
Dysgonomonas(100)	Otu019585	0	0	0	0	0.000110387459984546
Dysgonomonas(100)	Otu019586	0	0	0	0	0.000110387459984546
Dysgonomonas(100)	Otu019587	0	0	0	0	0.000110387459984546
Dysgonomonas(100)	Otu019589	0	0	0	0	0.000110387459984546
Dysgonomonas(100)	Otu019590	0	0	0	0	0.000110387459984546
Dysgonomonas(100)	Otu019602	0	0	0	0	0.000110387459984546
Dysgonomonas(100)	Otu019609	0	0	0	0	0.000110387459984546
Dysgonomonas(100)	Otu019660	0	0	0	0.00012482836100362	0
Dysgonomonas(100)	Otu019670	0	0	0	0.00012482836100362	0
Dysgonomonas(100)	Otu019680	0	0	0	0.00012482836100362	0
Dysgonomonas(100)	Otu019689	0	0	0	0.00012482836100362	0
Dysgonomonas(100)	Otu019691	0	0	0	0.00012482836100362	0
Dysgonomonas(100)	Otu019853	0	0	0	0.00012482836100362	0
Dysgonomonas(100)	Otu019854	0	0	0	0.00012482836100362	0
Dysgonomonas(100)	Otu019855	0	0	0	0.00012482836100362	0
Dysgonomonas(100)	Otu019866	0	0	0	0.00012482836100362	0
Dysgonomonas(100)	Otu019868	0	0	0	0.00012482836100362	0

Dysgonomonas(100)	Otu019876	0	0	0	0	0.000110387459984546
Dysgonomonas(100)	Otu019881	0	0	0	0.00012482836100362	0
Dysgonomonas(100)	Otu019891	0	0	0	0.00012482836100362	0
unclassified(100)	Otu019893	0	0	0	0	0.000110387459984546
Dysgonomonas(100)	Otu019896	0	0	0	0.00012482836100362	0
Dysgonomonas(100)	Otu019936	0	0	0	0	0.000110387459984546
Dysgonomonas(100)	Otu019937	0	0	0	0	0.000110387459984546
Dysgonomonas(100)	Otu019938	0	0	0	0	0.000110387459984546
Dysgonomonas(100)	Otu019941	0	0	0	0	0.000110387459984546
Dysgonomonas(100)	Otu019942	0	0	0	0	0.000110387459984546
Dysgonomonas(100)	Otu019960	0	0	0	0.00012482836100362	0
Dysgonomonas(100)	Otu019962	0	0	0	0.00012482836100362	0
Dysgonomonas(100)	Otu020269	0	0	0	0.00012482836100362	0
Dysgonomonas(100)	Otu020280	0	0	0	0.00012482836100362	0
Dysgonomonas(100)	Otu020284	0	0	0	0.00012482836100362	0
Dysgonomonas(100)	Otu020289	0	0	0	0.00012482836100362	0
unclassified(100)	Otu020298	0	0	0	0.00012482836100362	0
Dysgonomonas(100)	Otu020658	0	0.000124968757810547	0	0	0
Dysgonomonas(100)	Otu020660	0	0.000124968757810547	0	0	0
Dysgonomonas(100)	Otu020663	0	0.000124968757810547	0	0	0
Dysgonomonas(100)	Otu020695	0	0.000124968757810547	0	0	0
Dysgonomonas(100)	Otu020801	0	0.000124968757810547	0	0	0
Dysgonomonas(100)	Otu020831	0	0.000124968757810547	0	0	0
Dysgonomonas(100)	Otu020843	0	0.000124968757810547	0	0	0
Dysgonomonas(100)	Otu020850	0	0.000124968757810547	0	0	0
Dysgonomonas(100)	Otu020886	0	0.000124968757810547	0	0	0
Dysgonomonas(100)	Otu021354	0	0	0.000109075043630017	0	0
Dysgonomonas(100)	Otu021358	0	0	0.000109075043630017	0	0
Dysgonomonas(100)	Otu021360	0	0	0.000109075043630017	0	0
Dysgonomonas(100)	Otu021361	0	0	0.000109075043630017	0	0
Dysgonomonas(100)	Otu021362	0	0	0.000109075043630017	0	0
Dysgonomonas(100)	Otu021367	0	0	0.000109075043630017	0	0
Dysgonomonas(100)	Otu021373	0	0	0.000109075043630017	0	0

Dysgonomonas(100)	Otu021374	0	0	0.000109075043630017	0	0
Dysgonomonas(100)	Otu021376	0	0	0.000109075043630017	0	0
Dysgonomonas(100)	Otu021393	0	0	0.000109075043630017	0	0
Dysgonomonas(100)	Otu021399	0	0	0.000109075043630017	0	0
Dysgonomonas(100)	Otu021402	0	0	0.000109075043630017	0	0
Dysgonomonas(100)	Otu021403	0	0	0.000109075043630017	0	0
Dysgonomonas(100)	Otu021404	0	0	0.000109075043630017	0	0
Dysgonomonas(100)	Otu021415	0	0	0.000109075043630017	0	0
Dysgonomonas(100)	Otu021417	0	0	0.000109075043630017	0	0
Dysgonomonas(100)	Otu021429	0	0	0.000109075043630017	0	0
unclassified(100)	Otu021442	0	0	0.000109075043630017	0	0
Dysgonomonas(100)	Otu021445	0	0	0.000109075043630017	0	0
Dysgonomonas(100)	Otu021447	0	0	0.000109075043630017	0	0
Dysgonomonas(100)	Otu021449	0	0	0.000109075043630017	0	0
Dysgonomonas(100)	Otu021460	0	0	0.000109075043630017	0	0
Dysgonomonas(100)	Otu021466	0	0	0	0.00012482836100362	0
Dysgonomonas(100)	Otu021470	0	0	0.000109075043630017	0	0
Dysgonomonas(100)	Otu021482	0	0	0	0.00012482836100362	0
unclassified(100)	Otu021483	0	0	0	0.00012482836100362	0
Dysgonomonas(100)	Otu021490	0	0	0	0.00012482836100362	0
Dysgonomonas(100)	Otu021491	0	0	0.000109075043630017	0	0
Dysgonomonas(100)	Otu021502	0	0	0	0	0.000110387459984546
Dysgonomonas(100)	Otu021507	0	0	0	0	0.000110387459984546
Dysgonomonas(100)	Otu021509	0	0	0	0	0.000110387459984546
Dysgonomonas(100)	Otu021515	0	0	0	0	0.000110387459984546
unclassified(100)	Otu021519	0	0	0	0	0.000110387459984546
Dysgonomonas(100)	Otu021520	0	0	0	0	0.000110387459984546
Dysgonomonas(100)	Otu021521	0	0	0	0	0.000110387459984546
Dysgonomonas(100)	Otu021555	0	0	0.000109075043630017	0	0
Dysgonomonas(100)	Otu021558	0	0	0.000109075043630017	0	0
Dysgonomonas(100)	Otu021560	0	0	0.000109075043630017	0	0
Dysgonomonas(100)	Otu021562	0	0	0.000109075043630017	0	0
Dysgonomonas(100)	Otu021563	0	0	0.000109075043630017	0	0

Dysgonomonas(100)	Otu021565	0	0	0.000109075043630017	0	0
Dysgonomonas(100)	Otu021628	0	0	0	0	0.000110387459984546
Dysgonomonas(100)	Otu021629	0	0	0	0	0.000110387459984546
Dysgonomonas(100)	Otu021761	0	0	0	0	0.000110387459984546
Dysgonomonas(100)	Otu021762	0	0	0	0	0.000110387459984546
Dysgonomonas(100)	Otu021934	0	0	0	0.00012482836100362	0
Dysgonomonas(100)	Otu021949	0	0	0	0.00012482836100362	0
Dysgonomonas(100)	Otu021967	0	0	0	0.00012482836100362	0
Dysgonomonas(100)	Otu021976	0	0	0	0.00012482836100362	0
Dysgonomonas(100)	Otu021989	0	0	0	0.00012482836100362	0
Dysgonomonas(100)	Otu021997	0	0	0	0.00012482836100362	0
Dysgonomonas(100)	Otu022022	0	0	0	0.00012482836100362	0
Dysgonomonas(100)	Otu022024	0	0	0	0.00012482836100362	0
Dysgonomonas(100)	Otu022035	0	0	0	0.00012482836100362	0
Dysgonomonas(100)	Otu022042	0	0	0	0.00012482836100362	0
Dysgonomonas(100)	Otu022045	0	0	0.000109075043630017	0	0
Dysgonomonas(100)	Otu022047	0	0	0.000109075043630017	0	0
Dysgonomonas(100)	Otu022049	0	0	0.000109075043630017	0	0
Dysgonomonas(100)	Otu022066	0	0	0	0	0.000110387459984546
Dysgonomonas(100)	Otu022069	0	0	0	0.00012482836100362	0
Dysgonomonas(100)	Otu022084	0	0	0.000109075043630017	0	0
Dysgonomonas(100)	Otu022090	0	0	0.000109075043630017	0	0
Dysgonomonas(100)	Otu022094	0	0	0.000109075043630017	0	0
Dysgonomonas(100)	Otu022107	0	0	0	0.00012482836100362	0
Dysgonomonas(100)	Otu022125	0	0	0	0	0.000110387459984546
Dysgonomonas(100)	Otu022126	0	0	0	0	0.000110387459984546
Dysgonomonas(100)	Otu022128	0	0	0	0	0.000110387459984546
Dysgonomonas(100)	Otu022129	0	0	0	0	0.000110387459984546
unclassified(100)	Otu022138	0	0	0	0	0.000110387459984546
Dysgonomonas(100)	Otu022139	0	0	0	0	0.000110387459984546
Dysgonomonas(100)	Otu022140	0	0	0	0	0.000110387459984546
Dysgonomonas(100)	Otu022144	0	0	0	0.00012482836100362	0
Dysgonomonas(100)	Otu022153	0	0	0	0.00012482836100362	0

Dysgonomonas(100)	Otu022220	0	0	0.000109075043630017	0	0
Dysgonomonas(100)	Otu022223	0	0	0.000109075043630017	0	0
Dysgonomonas(100)	Otu022224	0	0	0.000109075043630017	0	0
Dysgonomonas(100)	Otu022228	0	0	0.000109075043630017	0	0
Dysgonomonas(100)	Otu022233	0	0	0.000109075043630017	0	0
Dysgonomonas(100)	Otu022241	0	0	0.000109075043630017	0	0
Dysgonomonas(100)	Otu022244	0	0	0.000109075043630017	0	0
Dysgonomonas(100)	Otu022258	0	0	0.000109075043630017	0	0
Dysgonomonas(100)	Otu022261	0	0	0.000109075043630017	0	0
Dysgonomonas(100)	Otu022263	0	0	0.000109075043630017	0	0
Dysgonomonas(100)	Otu022266	0	0	0.000109075043630017	0	0
Dysgonomonas(100)	Otu022992	0	0	0	0	0.000110387459984546
Dysgonomonas(100)	Otu022993	0	0	0	0	0.000110387459984546
Dysgonomonas(100)	Otu023007	0	0	0	0	0.000110387459984546
Dysgonomonas(100)	Otu023013	0	0	0	0	0.000110387459984546
Dysgonomonas(100)	Otu023014	0	0	0	0	0.000110387459984546
Dysgonomonas(100)	Otu023015	0	0	0	0	0.000110387459984546
Dysgonomonas(100)	Otu023022	0	0	0	0	0.000110387459984546
unclassified(100)	Otu023026	0	0	0	0	0.000110387459984546
Dysgonomonas(100)	Otu023034	0	0	0	0	0.000110387459984546
Dysgonomonas(100)	Otu023036	0	0	0	0	0.000110387459984546
Streptococcus(100)	Otu023322	0	0	0	0	0.000110387459984546
Streptococcus(100)	Otu023326	0	0	0	0	0.000110387459984546
Streptococcus(100)	Otu023331	0	0	0	0	0.000110387459984546
Streptococcus(100)	Otu023334	0	0	0	0	0.000110387459984546
Streptococcus(100)	Otu023353	0	0.000124968757810547	0	0	0
Streptococcus(100)	Otu023355	0	0.000124968757810547	0	0	0
Streptococcus(100)	Otu023361	0	0.000124968757810547	0	0	0
Streptococcus(100)	Otu023377	0	0	0	0	0.000110387459984546
Streptococcus(100)	Otu023390	0	0	0	0	0.000110387459984546
Streptococcus(100)	Otu023682	0	0	0	0.00012482836100362	0
Streptococcus(100)	Otu023689	0	0	0	0.00012482836100362	0
Streptococcus(100)	Otu023708	0	0	0	0.00012482836100362	0

Streptococcus(100)	Otu023713	0	0	0.000109075043630017	0	0
Streptococcus(100)	Otu023721	0	0	0	0.00012482836100362	0
unclassified(100)	Otu023723	0	0	0	0.00012482836100362	0
Streptococcus(100)	Otu023725	0	0	0	0.00012482836100362	0
Streptococcus(100)	Otu023731	0	0	0	0.00012482836100362	0
Streptococcus(100)	Otu023734	0	0	0	0.00012482836100362	0
Streptococcus(100)	Otu023738	0	0	0	0.00012482836100362	0
Streptococcus(100)	Otu023767	0	0	0	0	0.00077271221989182
Streptococcus(100)	Otu023784	0	0	0	0.00012482836100362	0
Streptococcus(100)	Otu023872	0	0.000124968757810547	0	0	0
Streptococcus(100)	Otu023888	0	0	0.000109075043630017	0	0
Streptococcus(100)	Otu023995	0.0000778513040093422	0	0	0	0
Streptococcus(100)	Otu024001	0.0000778513040093422	0	0	0	0
Streptococcus(100)	Otu024838	0	0	0	0	0.000110387459984546
Pedobacter(100)	Otu025128	0	0.000124968757810547	0	0	0
Pedobacter(100)	Otu025150	0	0.000124968757810547	0	0	0
Pedobacter(100)	Otu025157	0	0.000124968757810547	0	0	0
Pedobacter(100)	Otu025175	0	0.000124968757810547	0	0	0
Pedobacter(100)	Otu025179	0	0.000124968757810547	0	0	0
Pedobacter(100)	Otu025405	0	0	0.000109075043630017	0	0
Pedobacter(100)	Otu025443	0	0.000124968757810547	0	0	0
Pedobacter(100)	Otu025457	0	0	0	0.00012482836100362	0
Ralstonia(100)	Otu025750	0.0000778513040093422	0	0	0	0
Ralstonia(100)	Otu025752	0.0000778513040093422	0	0	0	0
Polynucleobacter(100)	Otu025759	0	0.000124968757810547	0	0	0
unclassified(100)	Otu025762	0	0	0	0	0.000110387459984546
unclassified(100)	Otu025765	0	0	0	0	0.000110387459984546
unclassified(100)	Otu025823	0	0.000124968757810547	0	0	0
Pedobacter(100)	Otu026703	0	0.000124968757810547	0	0	0
Pedobacter(100)	Otu026706	0	0.000124968757810547	0	0	0
unclassified(100)	Otu026735	0	0	0	0.00012482836100362	0
Sphingobacterium(100)	Otu026767	0	0	0	0.00012482836100362	0
Pedobacter(100)	Otu026774	0	0	0	0	0.000110387459984546

Pedobacter(100)	Otu026809	0	0	0	0.00012482836100362	0
unclassified(100)	Otu027081	0	0	0.000109075043630017	0	0
unclassified(100)	Otu027110	0	0.000124968757810547	0	0	0
unclassified(100)	Otu027138	0	0	0	0.00012482836100362	0
unclassified(100)	Otu027167	0	0	0.000109075043630017	0	0
unclassified(100)	Otu027219	0	0.000124968757810547	0	0	0
unclassified(100)	Otu027256	0	0	0	0.00012482836100362	0
unclassified(100)	Otu027258	0	0	0	0.00012482836100362	0
unclassified(100)	Otu027265	0	0	0	0.00012482836100362	0
unclassified(100)	Otu027275	0	0	0	0.00012482836100362	0
unclassified(100)	Otu027280	0	0	0	0.00012482836100362	0
unclassified(100)	Otu027292	0	0	0	0.00012482836100362	0
unclassified(100)	Otu027295	0	0	0	0.00012482836100362	0
unclassified(100)	Otu027344	0	0	0	0	0.000110387459984546
unclassified(100)	Otu027360	0	0	0	0	0.000110387459984546
unclassified(100)	Otu027381	0	0	0	0.00012482836100362	0
unclassified(100)	Otu027412	0	0	0	0.00012482836100362	0
unclassified(100)	Otu027419	0	0	0	0.00012482836100362	0
unclassified(100)	Otu027435	0	0	0	0.00012482836100362	0
unclassified(100)	Otu027494	0	0	0	0.00012482836100362	0
unclassified(100)	Otu027513	0	0	0	0.00012482836100362	0
unclassified(100)	Otu027539	0	0	0	0	0.000110387459984546
unclassified(100)	Otu027548	0	0	0	0.00012482836100362	0
unclassified(100)	Otu027550	0	0	0	0.00012482836100362	0
unclassified(100)	Otu027552	0	0	0	0.00012482836100362	0
unclassified(100)	Otu027560	0	0	0	0	0.000110387459984546
unclassified(100)	Otu027562	0	0	0	0	0.000110387459984546
unclassified(100)	Otu027567	0	0	0	0.00012482836100362	0
unclassified(100)	Otu027573	0	0	0	0	0.000110387459984546
unclassified(100)	Otu027574	0	0	0	0	0.000110387459984546
unclassified(100)	Otu027624	0	0	0	0	0.000110387459984546
unclassified(100)	Otu027816	0	0.000124968757810547	0	0	0
unclassified(100)	Otu027824	0	0.000124968757810547	0	0	0

unclassified(100)	Otu027830	0	0.000124968757810547	0	0	0
unclassified(100)	Otu027840	0	0.000124968757810547	0	0	0
unclassified(100)	Otu027886	0	0.000124968757810547	0	0	0
unclassified(100)	Otu027910	0	0.000124968757810547	0	0	0
unclassified(100)	Otu027916	0	0.000124968757810547	0	0	0
unclassified(100)	Otu027920	0	0.000124968757810547	0	0	0
unclassified(100)	Otu027924	0	0.000124968757810547	0	0	0
unclassified(100)	Otu027928	0	0.000124968757810547	0	0	0
unclassified(100)	Otu027930	0	0.000124968757810547	0	0	0
unclassified(100)	Otu027934	0	0	0	0	0.000110387459984546
unclassified(100)	Otu027943	0	0	0	0	0.000110387459984546
unclassified(100)	Otu027986	0	0.000124968757810547	0	0	0
unclassified(100)	Otu027988	0	0.000124968757810547	0	0	0
unclassified(100)	Otu028005	0	0.000124968757810547	0	0	0
unclassified(100)	Otu028009	0	0.000124968757810547	0	0	0
unclassified(100)	Otu028311	0	0.000124968757810547	0	0	0
unclassified(100)	Otu028320	0.0000778513040093422	0	0	0	0
unclassified(100)	Otu028332	0	0.000124968757810547	0	0	0
unclassified(100)	Otu028379	0	0.000124968757810547	0	0	0
unclassified(100)	Otu028398	0	0.000124968757810547	0	0	0
unclassified(100)	Otu028406	0	0.000124968757810547	0	0	0
Escherichia-Shigella(100)	Otu028407	0	0.000124968757810547	0	0	0
unclassified(100)	Otu028628	0	0	0.000109075043630017	0	0
Ewingella(100)	Otu028634	0	0	0.000109075043630017	0	0
unclassified(100)	Otu028636	0	0	0.000109075043630017	0	0
unclassified(100)	Otu028644	0	0	0.000109075043630017	0	0
unclassified(100)	Otu028665	0	0	0.000109075043630017	0	0
unclassified(100)	Otu028668	0	0	0.000109075043630017	0	0
unclassified(100)	Otu028671	0	0	0.000109075043630017	0	0
unclassified(100)	Otu028680	0	0	0	0.00012482836100362	0
unclassified(100)	Otu028722	0	0	0.000109075043630017	0	0
unclassified(100)	Otu028729	0	0	0	0.00012482836100362	0
unclassified(100)	Otu028734	0	0	0.000109075043630017	0	0

unclassified(100)	Otu029111	0	0.000124968757810547	0	0	0
unclassified(100)	Otu029113	0	0	0	0.00012482836100362	0
unclassified(100)	Otu029120	0	0	0	0.00012482836100362	0
unclassified(100)	Otu029122	0	0	0	0.00012482836100362	0
unclassified(100)	Otu029125	0	0	0	0.00012482836100362	0
unclassified(100)	Otu029168	0	0	0	0.00012482836100362	0
unclassified(100)	Otu029179	0	0	0	0	0.000110387459984546
unclassified(100)	Otu029203	0	0.000124968757810547	0	0	0
unclassified(100)	Otu029210	0	0	0	0	0.000110387459984546
unclassified(100)	Otu029672	0.0000778513040093422	0	0	0	0
unclassified(100)	Otu029699	0	0.000124968757810547	0	0	0
unclassified(100)	Otu034823	0	0	0	0	0.000110387459984546
Hyphomicrobium(100)	Otu035288	0	0	0	0.00012482836100362	0
Hyphomicrobium(100)	Otu035293	0	0	0.000109075043630017	0	0
Hyphomicrobium(100)	Otu035313	0	0	0	0	0.000110387459984546
Hyphomicrobium(100)	Otu035442	0	0	0	0	0.000110387459984546
Devosia(100)	Otu035445	0	0.000124968757810547	0	0	0
Hyphomicrobium(100)	Otu035460	0	0	0.000109075043630017	0	0
Hyphomicrobium(100)	Otu035486	0.0000778513040093422	0	0	0	0
Hyphomicrobium(100)	Otu035489	0.0000778513040093422	0	0	0	0
unclassified(100)	Otu035492	0.0000778513040093422	0	0	0	0
unclassified(100)	Otu035523	0	0	0	0.00012482836100362	0
unclassified(100)	Otu035526	0.0000778513040093422	0	0	0	0
unclassified(100)	Otu035527	0.0000778513040093422	0	0	0	0
unclassified(100)	Otu035585	0	0	0	0.00012482836100362	0
unclassified(100)	Otu035591	0	0.000124968757810547	0	0	0
unclassified(100)	Otu035594	0	0.000124968757810547	0	0	0
unclassified(100)	Otu035595	0	0	0.000109075043630017	0	0
Gemella(100)	Otu035622	0	0	0	0.00012482836100362	0
Gemella(100)	Otu035631	0	0	0	0.00012482836100362	0
Gemella(100)	Otu035636	0	0	0	0	0.000110387459984546
Gemella(100)	Otu035641	0	0	0.000109075043630017	0	0
unclassified(100)	Otu035803	0.0000778513040093422	0	0	0	0

unclassified(100)	Otu035804	0.0000778513040093422	0	0	0	0
unclassified(100)	Otu035850	0.0000778513040093422	0	0	0	0
unclassified(100)	Otu035851	0.0000778513040093422	0	0	0	0
unclassified(100)	Otu036061	0	0	0	0	0.000110387459984546
unclassified(100)	Otu036105	0.0000778513040093422	0	0	0	0
unclassified(100)	Otu036118	0.0000778513040093422	0	0	0	0
unclassified(100)	Otu036119	0.0000778513040093422	0	0	0	0
unclassified(100)	Otu036120	0.0000778513040093422	0	0	0	0
Ralstonia(100)	Otu036122	0.0000778513040093422	0	0	0	0
Ralstonia(100)	Otu036123	0.0000778513040093422	0	0	0	0
unclassified(100)	Otu036128	0	0	0.000109075043630017	0	0
unclassified(100)	Otu036130	0	0	0.000109075043630017	0	0
unclassified(100)	Otu036131	0	0	0.000109075043630017	0	0
unclassified(100)	Otu036138	0.0000778513040093422	0	0	0	0
unclassified(100)	Otu036140	0.0000778513040093422	0	0	0	0
Ralstonia(100)	Otu036143	0	0	0.000109075043630017	0	0
unclassified(100)	Otu036144	0	0	0.000109075043630017	0	0
unclassified(100)	Otu036145	0	0	0.000109075043630017	0	0
unclassified(100)	Otu036148	0	0.000124968757810547	0	0	0
Limnobacter(100)	Otu036149	0	0	0	0.00012482836100362	0
Ralstonia(100)	Otu036150	0	0	0	0.00012482836100362	0
unclassified(100)	Otu036157	0	0	0	0	0.000110387459984546
unclassified(100)	Otu036161	0	0	0	0	0.000110387459984546
Anaerococcus(100)	Otu036345	0	0.000124968757810547	0	0	0
Finegoldia(100)	Otu036355	0	0.000124968757810547	0	0	0
Anaerococcus(100)	Otu036363	0	0	0	0.00012482836100362	0
Anaerococcus(100)	Otu036364	0	0	0	0.00012482836100362	0
Peptoniphilus(100)	Otu036399	0	0.000124968757810547	0	0	0
Anaerococcus(100)	Otu036405	0	0	0	0.00012482836100362	0
Anaerococcus(100)	Otu036409	0	0	0	0.00012482836100362	0
Anaerococcus(100)	Otu036411	0	0	0	0	0.000110387459984546
Anaerococcus(100)	Otu036422	0	0	0	0.00012482836100362	0
Finegoldia(100)	Otu036449	0	0	0	0	0.000110387459984546

Prevotella(100)	Otu036598	0	0	0.000109075043630017	0	0
Prevotella(100)	Otu036610	0	0	0.000327225130890052	0	0
Prevotella(100)	Otu036612	0	0	0.000109075043630017	0	0
Prevotella(100)	Otu036678	0	0	0	0	0.000110387459984546
Prevotella(100)	Otu036679	0	0	0	0	0.000110387459984546
Prevotella(100)	Otu036680	0	0	0	0	0.000110387459984546
unclassified(100)	Otu037718	0	0	0	0.00012482836100362	0
unclassified(100)	Otu037725	0	0	0.000109075043630017	0	0
unclassified(100)	Otu037824	0	0	0	0.00012482836100362	0
unclassified(100)	Otu037826	0	0	0	0	0.000110387459984546
unclassified(100)	Otu037827	0	0	0	0	0.000110387459984546
Reyranella(100)	Otu038043	0.0000778513040093422	0	0	0	0
unclassified(100)	Otu038107	0	0	0	0	0.000110387459984546
unclassified(100)	Otu038117	0	0	0	0	0.000441549839938183
Treponema(100)	Otu038118	0	0	0	0	0.000662324759907275
unclassified(100)	Otu038288	0	0.000499875031242189	0.000218150087260035	0.00074897016602172	0.00121426205983
unclassified(100)	Otu038311	0	0	0	0.00037448508301086	0
Blastocatella(100)	Otu038383	0.0000778513040093422	0	0	0	0
uncultured(100)	Otu038416	0	0	0.000109075043630017	0	0
unclassified(100)	Otu038431	0	0	0.000109075043630017	0	0
uncultured(100)	Otu038432	0	0	0	0	0.000110387459984546
Halomonas(100)	Otu038457	0	0	0	0.00012482836100362	0
Rheinheimera(100)	Otu038515	0	0	0.000109075043630017	0	0
unclassified(100)	Otu038538	0	0	0	0	0.000331162379953637
SM1A02(100)	Otu038549	0	0	0.000109075043630017	0	0
unclassified(100)	Otu038578	0	0.000124968757810547	0	0	0
unclassified(100)	Otu038637	0	0.000124968757810547	0	0	0
unclassified(100)	Otu038658	0	0.000124968757810547	0	0	0
unclassified(100)	Otu038664	0	0	0	0	0.000110387459984546
unclassified(100)	Otu038665	0	0	0	0	0.000110387459984546
unclassified(100)	Otu038666	0.0000778513040093422	0	0	0	0
unclassified(100)	Otu038667	0	0	0	0	0.000110387459984546
unclassified(100)	Otu038668	0	0	0	0	0.000110387459984546

unclassified(100)	Otu038673	0	0	0	0	0.000110387459984546
unclassified(100)	Otu038675	0	0	0	0	0.000110387459984546
unclassified(100)	Otu038679	0	0	0	0	0.000110387459984546
unclassified(100)	Otu038683	0	0	0	0	0.000110387459984546
unclassified(100)	Otu038684	0	0	0	0	0.000110387459984546
unclassified(100)	Otu038696	0	0.000124968757810547	0	0	0
unclassified(100)	Otu038704	0	0.000124968757810547	0	0	0
unclassified(100)	Otu038705	0	0.000124968757810547	0	0	0
unclassified(100)	Otu038706	0	0.000124968757810547	0	0	0
unclassified(100)	Otu038709	0	0.000124968757810547	0	0	0
unclassified(100)	Otu038712	0	0	0.000109075043630017	0	0
unclassified(100)	Otu038714	0	0	0.000109075043630017	0	0
unclassified(100)	Otu038719	0	0	0.000109075043630017	0	0
unclassified(100)	Otu038721	0	0.000124968757810547	0	0	0
unclassified(100)	Otu038732	0	0.000124968757810547	0	0	0
unclassified(100)	Otu038739	0	0.000124968757810547	0	0	0
unclassified(100)	Otu038740	0	0.000124968757810547	0	0	0
unclassified(100)	Otu038750	0	0.000124968757810547	0	0	0
unclassified(100)	Otu038754	0	0.000124968757810547	0	0	0
unclassified(100)	Otu038758	0	0	0	0	0.000110387459984546
unclassified(100)	Otu038759	0	0	0	0	0.000110387459984546
unclassified(100)	Otu038760	0	0	0	0	0.000110387459984546
unclassified(100)	Otu038763	0	0	0	0	0.000110387459984546
unclassified(100)	Otu038764	0.0000778513040093422	0	0	0	0
unclassified(100)	Otu038766	0	0	0	0	0.000110387459984546
unclassified(100)	Otu038768	0	0	0	0	0.000110387459984546
unclassified(100)	Otu038781	0	0	0	0	0.000110387459984546
unclassified(100)	Otu038784	0.0000778513040093422	0	0	0	0
unclassified(100)	Otu038785	0	0	0	0	0.000110387459984546
unclassified(100)	Otu038787	0	0	0	0	0.000110387459984546
unclassified(100)	Otu038792	0	0	0	0.00012482836100362	0
unclassified(100)	Otu038795	0	0	0	0	0.000110387459984546
unclassified(100)	Otu038796	0	0	0	0	0.000110387459984546

unclassified(100)	Otu038801	0	0	0	0	0.000110387459984546
unclassified(100)	Otu038805	0.0000778513040093422	0	0	0	0
unclassified(100)	Otu038807	0	0	0	0	0.000110387459984546
unclassified(100)	Otu038808	0.0000778513040093422	0	0	0	0
unclassified(100)	Otu038809	0	0	0	0	0.000110387459984546
unclassified(100)	Otu038838	0	0	0	0	0.000110387459984546
unclassified(100)	Otu038845	0	0	0	0	0.000110387459984546
unclassified(100)	Otu038847	0.0000778513040093422	0	0	0	0
unclassified(100)	Otu038851	0	0	0	0	0.000110387459984546
unclassified(100)	Otu038855	0	0	0	0	0.000110387459984546
unclassified(100)	Otu038869	0	0	0	0	0.000110387459984546
unclassified(100)	Otu038870	0	0	0	0	0.000110387459984546
unclassified(100)	Otu038871	0	0	0	0	0.000110387459984546
unclassified(100)	Otu038872	0.0000778513040093422	0	0	0	0
unclassified(100)	Otu038874	0	0	0	0	0.000110387459984546
unclassified(100)	Otu038875	0	0	0	0	0.000110387459984546
unclassified(100)	Otu038888	0	0.000124968757810547	0	0	0
unclassified(100)	Otu038889	0	0.000124968757810547	0	0	0
unclassified(100)	Otu038913	0	0.000124968757810547	0	0	0
unclassified(100)	Otu038921	0	0.000124968757810547	0	0	0
unclassified(100)	Otu038922	0	0.000124968757810547	0	0	0
unclassified(100)	Otu038924	0	0.000124968757810547	0	0	0
unclassified(100)	Otu038929	0	0.000124968757810547	0	0	0
unclassified(100)	Otu038940	0	0.000124968757810547	0	0	0
unclassified(100)	Otu038987	0	0.000124968757810547	0	0	0
unclassified(100)	Otu039004	0	0.000124968757810547	0	0	0
unclassified(100)	Otu039005	0	0.000124968757810547	0	0	0
unclassified(100)	Otu039012	0	0.000124968757810547	0	0	0
unclassified(100)	Otu039016	0	0.000124968757810547	0	0	0
unclassified(100)	Otu039020	0	0.000124968757810547	0	0	0
unclassified(100)	Otu039021	0	0.000124968757810547	0	0	0
unclassified(100)	Otu039026	0	0.000124968757810547	0	0	0
unclassified(100)	Otu039027	0	0.000124968757810547	0	0	0

unclassified(100)	Otu039031	0	0.000124968757810547	0	0	0
unclassified(100)	Otu039032	0	0.000124968757810547	0	0	0
unclassified(100)	Otu039033	0	0.000124968757810547	0	0	0
unclassified(100)	Otu039037	0	0.000124968757810547	0	0	0
unclassified(100)	Otu039043	0	0.000124968757810547	0	0	0
unclassified(100)	Otu039052	0	0.000124968757810547	0	0	0
unclassified(100)	Otu039053	0	0.000124968757810547	0	0	0
unclassified(100)	Otu039082	0	0.000124968757810547	0	0	0
unclassified(100)	Otu039083	0	0.000124968757810547	0	0	0
unclassified(100)	Otu039090	0	0.000124968757810547	0	0	0
unclassified(100)	Otu039093	0	0.000124968757810547	0	0	0
unclassified(100)	Otu039096	0	0.000124968757810547	0	0	0
unclassified(100)	Otu039110	0	0.000124968757810547	0	0	0
unclassified(100)	Otu039111	0	0.000124968757810547	0	0	0
unclassified(100)	Otu039114	0	0.000124968757810547	0	0	0
unclassified(100)	Otu039222	0	0	0	0.00012482836100362	0
Terrimonas(100)	Otu039229	0	0	0	0.00012482836100362	0
unclassified(100)	Otu039370	0	0	0	0.00012482836100362	0
unclassified(100)	Otu039371	0	0	0	0.00012482836100362	0
unclassified(100)	Otu039372	0	0	0	0.00012482836100362	0
unclassified(100)	Otu039383	0.0000778513040093422	0	0	0	0
unclassified(100)	Otu039390	0.0000778513040093422	0	0	0	0
unclassified(100)	Otu039392	0.0000778513040093422	0	0	0	0
unclassified(100)	Otu039395	0	0	0	0.00012482836100362	0
unclassified(100)	Otu039396	0	0	0	0.00012482836100362	0
unclassified(100)	Otu039399	0	0	0	0.00012482836100362	0
unclassified(100)	Otu039403	0.0000778513040093422	0	0	0	0
unclassified(100)	Otu039410	0.0000778513040093422	0	0	0	0
unclassified(100)	Otu039411	0	0	0	0.00012482836100362	0
unclassified(100)	Otu039413	0	0	0	0	0.000110387459984546
unclassified(100)	Otu039414	0	0	0	0	0.000110387459984546
unclassified(100)	Otu039416	0	0	0	0.00012482836100362	0
unclassified(100)	Otu039419	0	0	0	0.00012482836100362	0

unclassified(100)	Otu039426	0	0	0	0	0.000331162379953637
unclassified(100)	Otu039428	0.0000778513040093422	0	0	0	0
unclassified(100)	Otu039430	0	0	0	0.00012482836100362	0
unclassified(100)	Otu039433	0	0.000124968757810547	0	0	0
unclassified(100)	Otu039435	0	0.000124968757810547	0	0	0
unclassified(100)	Otu039436	0	0	0	0	0.000110387459984546
unclassified(100)	Otu039439	0.0000778513040093422	0	0	0	0
unclassified(100)	Otu039441	0	0	0	0	0.000110387459984546
unclassified(100)	Otu039442	0	0	0	0	0.000110387459984546
unclassified(100)	Otu039443	0	0	0	0	0.000110387459984546
unclassified(100)	Otu039446	0	0	0	0	0.000110387459984546
unclassified(100)	Otu039447	0	0	0	0	0.000110387459984546
unclassified(100)	Otu039450	0	0	0	0	0.000110387459984546
unclassified(100)	Otu039455	0	0	0	0.00012482836100362	0
unclassified(100)	Otu039462	0	0	0	0	0.000110387459984546
unclassified(100)	Otu039465	0	0.000124968757810547	0	0	0
unclassified(100)	Otu039485	0	0.000124968757810547	0	0	0
unclassified(100)	Otu039578	0	0	0	0.00012482836100362	0
unclassified(100)	Otu039584	0	0	0	0	0.000220774919969092
unclassified(100)	Otu039600	0.0000778513040093422	0	0	0	0
unclassified(100)	Otu039740	0.0000778513040093422	0	0	0	0
unclassified(100)	Otu039849	0.0000778513040093422	0	0	0	0
unclassified(100)	Otu039865	0.0000778513040093422	0	0	0	0
unclassified(100)	Otu039867	0.0000778513040093422	0	0	0	0
unclassified(100)	Otu039868	0.0000778513040093422	0	0	0	0
unclassified(100)	Otu039870	0.0000778513040093422	0	0	0	0
unclassified(100)	Otu039890	0.0000778513040093422	0	0	0	0
unclassified(100)	Otu039892	0	0	0	0	0.000110387459984546
unclassified(100)	Otu039895	0	0	0	0	0.000110387459984546
unclassified(100)	Otu039896	0	0	0	0	0.000110387459984546
unclassified(100)	Otu039899	0	0	0	0	0.000110387459984546
unclassified(100)	Otu039902	0	0	0	0	0.000110387459984546
unclassified(100)	Otu039913	0	0	0	0	0.000110387459984546

unclassified(100)	Otu039917	0	0	0	0	0.000110387459984546
unclassified(100)	Otu039920	0	0	0	0	0.000110387459984546
unclassified(100)	Otu039921	0.0000778513040093422	0	0	0	0
unclassified(100)	Otu039937	0	0	0	0	0.000110387459984546
unclassified(100)	Otu039941	0	0	0	0	0.000110387459984546
unclassified(100)	Otu039943	0.0000778513040093422	0	0	0	0
unclassified(100)	Otu039944	0	0	0	0	0.000110387459984546
unclassified(100)	Otu039955	0.0000778513040093422	0	0	0	0
unclassified(100)	Otu039961	0.0000778513040093422	0	0	0	0
unclassified(100)	Otu039963	0.0000778513040093422	0	0	0	0
unclassified(100)	Otu039985	0.0000778513040093422	0	0	0	0
unclassified(100)	Otu040001	0	0	0	0	0.000110387459984546
unclassified(100)	Otu040003	0	0	0	0	0.000110387459984546
unclassified(100)	Otu040007	0.0000778513040093422	0	0	0	0
unclassified(100)	Otu040011	0	0	0	0	0.000110387459984546
unclassified(100)	Otu040012	0	0	0	0	0.000110387459984546
unclassified(100)	Otu040014	0	0	0	0	0.000110387459984546
unclassified(100)	Otu040034	0	0	0	0	0.000110387459984546
unclassified(100)	Otu040037	0.0000778513040093422	0	0	0	0
unclassified(100)	Otu040041	0.0000778513040093422	0	0	0	0
unclassified(100)	Otu040052	0	0	0	0	0.000110387459984546
unclassified(100)	Otu040055	0	0	0	0	0.000110387459984546
unclassified(100)	Otu040059	0	0	0	0	0.000110387459984546
unclassified(100)	Otu040063	0.0000778513040093422	0	0	0	0
unclassified(100)	Otu040086	0	0	0	0	0.000110387459984546
unclassified(100)	Otu040087	0	0	0	0	0.000110387459984546
unclassified(100)	Otu040090	0	0	0	0	0.000110387459984546
unclassified(100)	Otu040093	0	0	0	0	0.000110387459984546
unclassified(100)	Otu040096	0	0	0	0	0.000110387459984546
unclassified(100)	Otu040097	0	0	0	0	0.000110387459984546
unclassified(100)	Otu040099	0	0	0	0	0.000110387459984546
unclassified(100)	Otu040102	0	0	0	0	0.000110387459984546
unclassified(100)	Otu040173	0	0	0	0.00012482836100362	0

unclassified(100)	Otu040181	0.0000778513040093422	0	0	0	0
unclassified(100)	Otu040192	0.0000778513040093422	0	0	0	0
unclassified(100)	Otu040194	0	0	0	0.00012482836100362	0
unclassified(100)	Otu040195	0	0	0	0.00012482836100362	0
unclassified(100)	Otu040220	0.0000778513040093422	0	0	0	0
unclassified(100)	Otu040223	0	0	0	0.00012482836100362	0
unclassified(100)	Otu040224	0	0	0	0.00012482836100362	0
unclassified(100)	Otu040225	0	0	0	0.00012482836100362	0
unclassified(100)	Otu040260	0	0	0	0.00012482836100362	0
unclassified(100)	Otu040261	0	0	0	0.00012482836100362	0
unclassified(100)	Otu040278	0	0	0	0.00012482836100362	0
unclassified(100)	Otu040279	0	0	0	0.00012482836100362	0
unclassified(100)	Otu040288	0.0000778513040093422	0	0	0	0
unclassified(100)	Otu040337	0	0	0	0.00012482836100362	0
unclassified(100)	Otu040352	0.0000778513040093422	0	0	0	0
unclassified(100)	Otu040353	0	0	0	0.00012482836100362	0
unclassified(100)	Otu040356	0	0	0	0.00012482836100362	0
unclassified(100)	Otu040357	0.0000778513040093422	0	0	0	0
unclassified(100)	Otu040364	0	0	0	0.00012482836100362	0
unclassified(100)	Otu040366	0	0	0	0.00012482836100362	0
unclassified(100)	Otu040370	0	0	0	0.00012482836100362	0
unclassified(100)	Otu040376	0.0000778513040093422	0	0	0	0
unclassified(100)	Otu040381	0.0000778513040093422	0	0	0	0
unclassified(100)	Otu040386	0.0000778513040093422	0	0	0	0
unclassified(100)	Otu040389	0	0	0	0.00012482836100362	0
unclassified(100)	Otu040393	0	0	0	0.00012482836100362	0
unclassified(100)	Otu040394	0	0	0	0.00012482836100362	0
unclassified(100)	Otu040396	0	0	0	0.00012482836100362	0
unclassified(100)	Otu040398	0	0	0	0.00012482836100362	0
unclassified(100)	Otu040401	0.0000778513040093422	0	0	0	0
unclassified(100)	Otu040404	0.0000778513040093422	0	0	0	0
unclassified(100)	Otu040414	0.0000778513040093422	0	0	0	0
unclassified(100)	Otu040426	0.0000778513040093422	0	0	0	0

unclassified(100)	Otu040427	0.0000778513040093422	0	0	0	0
unclassified(100)	Otu040463	0.0000778513040093422	0	0	0	0
unclassified(100)	Otu040467	0.0000778513040093422	0	0	0	0
unclassified(100)	Otu040471	0	0	0	0.00012482836100362	0
unclassified(100)	Otu040472	0	0	0	0.00012482836100362	0
unclassified(100)	Otu040474	0.0000778513040093422	0	0	0	0
unclassified(100)	Otu040477	0	0	0	0.00012482836100362	0
unclassified(100)	Otu040479	0	0	0	0.00012482836100362	0
unclassified(100)	Otu040484	0	0	0	0.00012482836100362	0
unclassified(100)	Otu040490	0.0000778513040093422	0	0	0	0
unclassified(100)	Otu040492	0	0	0	0	0.000110387459984546
unclassified(100)	Otu040498	0	0	0	0	0.000110387459984546
unclassified(100)	Otu040499	0	0	0	0	0.000110387459984546
unclassified(100)	Otu040501	0.0000778513040093422	0	0	0	0
unclassified(100)	Otu040504	0	0	0	0	0.000110387459984546
unclassified(100)	Otu040507	0	0	0	0.00012482836100362	0
unclassified(100)	Otu040515	0	0	0	0.00012482836100362	0
unclassified(100)	Otu040525	0	0.000124968757810547	0	0	0
unclassified(100)	Otu040528	0	0	0	0.00012482836100362	0
unclassified(100)	Otu040530	0	0	0	0.00012482836100362	0
unclassified(100)	Otu040534	0	0	0	0.00012482836100362	0
unclassified(100)	Otu040535	0	0	0	0	0.000110387459984546
unclassified(100)	Otu040536	0.0000778513040093422	0	0	0	0
unclassified(100)	Otu040541	0	0	0	0.00012482836100362	0
unclassified(100)	Otu040554	0	0	0	0.00012482836100362	0
unclassified(100)	Otu040845	0.0000778513040093422	0	0	0	0
unclassified(100)	Otu040850	0.0000778513040093422	0	0	0	0
unclassified(100)	Otu040852	0.0000778513040093422	0	0	0	0
unclassified(100)	Otu040857	0.0000778513040093422	0	0	0	0
Hydrotalea(100)	Otu040858	0.0000778513040093422	0	0	0	0
unclassified(100)	Otu040880	0.0000778513040093422	0	0	0	0
unclassified(100)	Otu040884	0.0000778513040093422	0	0	0	0
Hydrotalea(100)	Otu040885	0.0000778513040093422	0	0	0	0

Hydrotalea(100)	Otu040895	0.0000778513040093422	0	0	0	0
unclassified(100)	Otu040900	0.0000778513040093422	0	0	0	0
Hydrotalea(100)	Otu040903	0.0000778513040093422	0	0	0	0
Hydrotalea(100)	Otu040908	0.0000778513040093422	0	0	0	0
Hydrotalea(100)	Otu040913	0.0000778513040093422	0	0	0	0
Sediminibacterium(100)	Otu040920	0.0000778513040093422	0	0	0	0
Hydrotalea(100)	Otu040938	0.0000778513040093422	0	0	0	0
unclassified(100)	Otu040943	0.0000778513040093422	0	0	0	0
unclassified(100)	Otu040958	0.0000778513040093422	0	0	0	0
Sediminibacterium(100)	Otu040962	0.0000778513040093422	0	0	0	0
unclassified(100)	Otu040990	0.0000778513040093422	0	0	0	0
unclassified(100)	Otu040991	0.0000778513040093422	0	0	0	0
Hydrotalea(100)	Otu040996	0.0000778513040093422	0	0	0	0
Sediminibacterium(100)	Otu041031	0.0000778513040093422	0	0	0	0
unclassified(100)	Otu041050	0.0000778513040093422	0	0	0	0
unclassified(100)	Otu041061	0.0000778513040093422	0	0	0	0
Hydrotalea(100)	Otu041069	0.0000778513040093422	0	0	0	0
Hydrotalea(100)	Otu041080	0.0000778513040093422	0	0	0	0
Hydrotalea(100)	Otu041085	0.0000778513040093422	0	0	0	0
unclassified(100)	Otu041704	0	0	0	0	0.000110387459984546
Lacibacter(100)	Otu041733	0	0	0.000109075043630017	0	0
Lacibacter(100)	Otu041742	0	0	0.000109075043630017	0	0
Lacibacter(100)	Otu041760	0	0	0	0	0.000110387459984546
unclassified(100)	Otu041763	0	0	0	0	0.000110387459984546
Sediminibacterium(100)	Otu041987	0	0	0.000109075043630017	0	0
Lacibacter(100)	Otu041988	0	0	0.000109075043630017	0	0
Lacibacter(100)	Otu041991	0	0	0.000109075043630017	0	0
unclassified(100)	Otu041995	0	0	0.000109075043630017	0	0
unclassified(100)	Otu041996	0	0.000124968757810547	0	0	0
unclassified(100)	Otu041999	0	0	0.000109075043630017	0	0
unclassified(100)	Otu042132	0.0000778513040093422	0	0	0	0
Hydrotalea(100)	Otu042144	0.0000778513040093422	0	0	0	0
Hydrotalea(100)	Otu042179	0.0000778513040093422	0	0	0	0

Hydrotalea(100)	Otu042197	0.0000778513040093422	0	0	0	0
Hydrotalea(100)	Otu042204	0.0000778513040093422	0	0	0	0
Hydrotalea(100)	Otu042212	0.0000778513040093422	0	0	0	0
Hydrotalea(100)	Otu042220	0.0000778513040093422	0	0	0	0
unclassified(100)	Otu042226	0.0000778513040093422	0	0	0	0
unclassified(100)	Otu042237	0.0000778513040093422	0	0	0	0
Sediminibacterium(100)	Otu042243	0.0000778513040093422	0	0	0	0
unclassified(100)	Otu042281	0.0000778513040093422	0	0	0	0
unclassified(100)	Otu042316	0.0000778513040093422	0	0	0	0
Lacibacter(100)	Otu042317	0	0	0.000109075043630017	0	0
Lacibacter(100)	Otu042321	0	0	0.000109075043630017	0	0
Hydrotalea(100)	Otu042346	0.0000778513040093422	0	0	0	0
unclassified(100)	Otu042347	0	0	0.000109075043630017	0	0
uncultured(100)	Otu042352	0	0.000124968757810547	0	0	0
unclassified(100)	Otu042359	0	0.000124968757810547	0	0	0
Lacibacter(100)	Otu042362	0	0	0.000109075043630017	0	0
unclassified(100)	Otu042370	0	0	0.000109075043630017	0	0
unclassified(100)	Otu042371	0	0	0.000109075043630017	0	0
Flavisolibacter(100)	Otu042372	0	0	0.000109075043630017	0	0
Lacibacter(100)	Otu042374	0	0	0.000109075043630017	0	0
unclassified(100)	Otu042377	0	0	0.000109075043630017	0	0
unclassified(100)	Otu042385	0	0	0.000109075043630017	0	0
Lacibacter(100)	Otu042415	0	0	0.000109075043630017	0	0
unclassified(100)	Otu042416	0	0	0.000109075043630017	0	0
unclassified(100)	Otu042420	0	0	0.000109075043630017	0	0
unclassified(100)	Otu042422	0	0	0.000109075043630017	0	0
unclassified(100)	Otu042438	0	0	0	0.00012482836100362	0
unclassified(100)	Otu042512	0	0.000124968757810547	0	0	0
uncultured(100)	Otu042514	0	0.000124968757810547	0	0	0
Lacibacter(100)	Otu042527	0	0	0.000109075043630017	0	0
uncultured(100)	Otu042549	0	0.000124968757810547	0	0	0
unclassified(100)	Otu042554	0.0000778513040093422	0	0	0	0
Flavisolibacter(100)	Otu042559	0	0.000124968757810547	0	0	0

unclassified(100)	Otu044656	0.0000778513040093422	0	0	0	0
unclassified(100)	Otu044663	0.000155702608018684	0	0	0	0
unclassified(100)	Otu044670	0.0000778513040093422	0	0	0	0
unclassified(100)	Otu044689	0.0000778513040093422	0	0	0	0
unclassified(100)	Otu044720	0.0000778513040093422	0	0	0	0
unclassified(100)	Otu044749	0.0000778513040093422	0	0	0	0
unclassified(100)	Otu044762	0.0000778513040093422	0	0	0	0
unclassified(100)	Otu045016	0	0.000124968757810547	0	0	0
unclassified(100)	Otu045018	0	0	0.000109075043630017	0	0
unclassified(100)	Otu045020	0	0	0.000109075043630017	0	0
unclassified(100)	Otu045024	0	0	0.000109075043630017	0	0
unclassified(100)	Otu045025	0	0	0.000109075043630017	0	0
unclassified(100)	Otu045029	0	0.000124968757810547	0	0	0
unclassified(100)	Otu045030	0	0	0.000109075043630017	0	0
unclassified(100)	Otu045032	0	0	0.000109075043630017	0	0
unclassified(100)	Otu045045	0	0	0.000109075043630017	0	0
unclassified(100)	Otu045055	0	0	0.000109075043630017	0	0
unclassified(100)	Otu045058	0	0	0.000109075043630017	0	0
unclassified(100)	Otu045059	0	0.000124968757810547	0	0	0
unclassified(100)	Otu045068	0.0000778513040093422	0	0	0	0
unclassified(100)	Otu045080	0	0	0.000109075043630017	0	0
unclassified(100)	Otu045081	0	0	0.000109075043630017	0	0
unclassified(100)	Otu045082	0	0	0.000109075043630017	0	0
unclassified(100)	Otu045087	0	0.000124968757810547	0	0	0
unclassified(100)	Otu045091	0	0	0.000109075043630017	0	0
unclassified(100)	Otu045092	0	0	0.000109075043630017	0	0
unclassified(100)	Otu045093	0	0	0.000109075043630017	0	0
unclassified(100)	Otu045097	0	0	0.000109075043630017	0	0
unclassified(100)	Otu045101	0	0	0.000109075043630017	0	0
unclassified(100)	Otu045105	0	0	0.000109075043630017	0	0
unclassified(100)	Otu045108	0	0	0.000109075043630017	0	0
unclassified(100)	Otu045111	0	0	0.000109075043630017	0	0
unclassified(100)	Otu045113	0	0	0.000109075043630017	0	0

unclassified(100)	Otu045115	0	0	0.000109075043630017	0	0
unclassified(100)	Otu045120	0	0	0.000109075043630017	0	0
unclassified(100)	Otu045121	0	0.000124968757810547	0	0	0
unclassified(100)	Otu045128	0	0.000124968757810547	0	0	0
unclassified(100)	Otu045130	0	0	0.000109075043630017	0	0
unclassified(100)	Otu045134	0	0.000124968757810547	0	0	0
unclassified(100)	Otu045159	0	0	0.000109075043630017	0	0
unclassified(100)	Otu045160	0	0	0.000109075043630017	0	0
unclassified(100)	Otu045165	0	0.000124968757810547	0	0	0
unclassified(100)	Otu045166	0	0	0.000109075043630017	0	0
unclassified(100)	Otu045168	0	0.000124968757810547	0	0	0
unclassified(100)	Otu045172	0	0	0	0	0.000110387459984546
unclassified(100)	Otu045173	0	0	0	0	0.000110387459984546
unclassified(100)	Otu045174	0	0	0	0	0.000110387459984546
unclassified(100)	Otu045175	0	0	0	0	0.000110387459984546
unclassified(100)	Otu045176	0	0	0	0	0.000110387459984546
unclassified(100)	Otu045178	0	0	0	0	0.000110387459984546
unclassified(100)	Otu045180	0	0	0	0	0.000110387459984546
unclassified(100)	Otu045183	0	0	0	0	0.000110387459984546
unclassified(100)	Otu045187	0.0000778513040093422	0	0	0	0
unclassified(100)	Otu045208	0	0.000124968757810547	0	0	0
unclassified(100)	Otu045216	0	0.000124968757810547	0	0	0
unclassified(100)	Otu045222	0	0.000124968757810547	0	0	0
unclassified(100)	Otu045237	0	0	0.000109075043630017	0	0
unclassified(100)	Otu045238	0	0	0.000109075043630017	0	0
unclassified(100)	Otu045239	0	0	0.000109075043630017	0	0
unclassified(100)	Otu045242	0	0	0.000109075043630017	0	0
unclassified(100)	Otu045243	0	0	0.000109075043630017	0	0
unclassified(100)	Otu045244	0	0	0.000109075043630017	0	0
unclassified(100)	Otu045245	0	0	0.000109075043630017	0	0
unclassified(100)	Otu045250	0	0	0.000109075043630017	0	0
unclassified(100)	Otu045272	0.0000778513040093422	0	0	0	0
unclassified(100)	Otu045312	0.0000778513040093422	0	0	0	0

unclassified(100)	Otu045314	0.0000778513040093422	0	0	0	0
unclassified(100)	Otu045375	0.0000778513040093422	0	0	0	0
unclassified(100)	Otu045386	0	0.000124968757810547	0	0	0
unclassified(100)	Otu045388	0	0.000124968757810547	0	0	0
unclassified(100)	Otu045391	0	0	0.000109075043630017	0	0
unclassified(100)	Otu045394	0	0.000124968757810547	0	0	0
unclassified(100)	Otu045400	0	0	0.000109075043630017	0	0
unclassified(100)	Otu045402	0	0	0.000109075043630017	0	0
unclassified(100)	Otu045403	0	0	0.000109075043630017	0	0
unclassified(100)	Otu045407	0	0	0.000109075043630017	0	0
unclassified(100)	Otu045408	0	0	0.000109075043630017	0	0
unclassified(100)	Otu045414	0	0	0.000109075043630017	0	0
unclassified(100)	Otu045417	0	0	0.000109075043630017	0	0
unclassified(100)	Otu045418	0	0	0.000109075043630017	0	0
unclassified(100)	Otu045423	0	0	0.000109075043630017	0	0
unclassified(100)	Otu045434	0	0	0.000109075043630017	0	0
unclassified(100)	Otu045438	0	0	0.000109075043630017	0	0
unclassified(100)	Otu045442	0	0	0.000109075043630017	0	0
unclassified(100)	Otu045481	0	0	0.000109075043630017	0	0
unclassified(100)	Otu045482	0	0.000124968757810547	0	0	0
unclassified(100)	Otu045484	0	0	0.000109075043630017	0	0
unclassified(100)	Otu045487	0	0	0.000109075043630017	0	0
Neochlamydia(100)	Otu046558	0.0000778513040093422	0	0	0	0
Neochlamydia(100)	Otu046561	0.0000778513040093422	0	0	0	0
unclassified(100)	Otu046586	0	0	0.000109075043630017	0	0
unclassified(100)	Otu046666	0	0	0.000109075043630017	0	0
unclassified(100)	Otu046704	0	0	0	0	0.000110387459984546
unclassified(100)	Otu046707	0	0	0	0	0.000441549839938183
unclassified(100)	Otu046708	0	0	0	0.00012482836100362	0
unclassified(100)	Otu046709	0	0	0.000109075043630017	0	0
unclassified(100)	Otu046712	0	0.000124968757810547	0	0	0
unclassified(100)	Otu046845	0	0	0.000109075043630017	0	0
unclassified(100)	Otu046890	0	0	0	0	0.000110387459984546

Neochlamydia(100)	Otu046895	0	0.000124968757810547	0	0	0
Hirschia(100)	Otu046982	0	0	0	0.00012482836100362	0
unclassified(100)	Otu047001	0	0	0	0	0.000110387459984546
uncultured(100)	Otu047030	0	0	0	0.00012482836100362	0
Incertae_Sedis(100)	Otu047055	0	0	0	0	0.000110387459984546
unclassified(100)	Otu047069	0	0	0.000109075043630017	0	0
unclassified(100)	Otu047070	0	0	0	0	0.000110387459984546
Sorangium(100)	Otu047181	0	0	0	0	0.000220774919969092
Coprococcus(100)	Otu047611	0	0	0.000109075043630017	0	0
Blautia(100)	Otu047612	0	0	0.000654450261780105	0	0
unclassified(100)	Otu047789	0	0.000124968757810547	0	0	0
unclassified(100)	Otu047817	0	0	0.000109075043630017	0	0
unclassified(100)	Otu047818	0	0	0.000109075043630017	0	0
unclassified(100)	Otu047819	0	0	0.000109075043630017	0	0
unclassified(100)	Otu047872	0	0	0	0.00012482836100362	0
unclassified(100)	Otu047880	0	0	0	0.00012482836100362	0
unclassified(100)	Otu047884	0	0.000124968757810547	0	0	0
unclassified(100)	Otu047893	0	0.000124968757810547	0	0	0
Ochrobactrum(100)	Otu047967	0	0	0	0	0.000110387459984546
Ochrobactrum(100)	Otu047992	0	0.000124968757810547	0	0	0
unclassified(100)	Otu048001	0	0.000124968757810547	0	0	0
unclassified(100)	Otu048041	0	0	0	0	0.000110387459984546
Dolosigranulum(100)	Otu048105	0	0.000124968757810547	0	0	0
Alkalibacterium(100)	Otu048167	0	0	0	0.00012482836100362	0
unclassified(100)	Otu048383	0	0.000124968757810547	0	0	0
unclassified(100)	Otu048387	0	0	0.000109075043630017	0	0
unclassified(100)	Otu050908	0	0.000124968757810547	0	0	0
unclassified(100)	Otu050910	0	0.000124968757810547	0	0	0
unclassified(100)	Otu050925	0	0	0	0.00012482836100362	0
unclassified(100)	Otu050927	0	0	0	0.00012482836100362	0
unclassified(100)	Otu050931	0	0	0	0.00012482836100362	0
unclassified(100)	Otu050935	0	0	0.000109075043630017	0	0
unclassified(100)	Otu050946	0	0.000124968757810547	0	0	0

unclassified(100)	Otu050971	0	0	0	0.00012482836100362	0
unclassified(100)	Otu050972	0	0	0.000109075043630017	0	0
unclassified(100)	Otu050975	0	0	0.000109075043630017	0	0
unclassified(100)	Otu051008	0	0.000124968757810547	0	0	0
unclassified(100)	Otu051012	0	0	0	0.00012482836100362	0
unclassified(100)	Otu051024	0	0.000124968757810547	0	0	0
unclassified(100)	Otu051025	0	0.000124968757810547	0	0	0
unclassified(100)	Otu051111	0	0.000124968757810547	0	0	0
unclassified(100)	Otu051113	0	0.000124968757810547	0	0	0
unclassified(100)	Otu051120	0.0000778513040093422	0	0	0	0
Leuconostoc(100)	Otu051789	0	0.000124968757810547	0	0	0
unclassified(100)	Otu052392	0	0	0	0.00012482836100362	0
unclassified(100)	Otu052414	0	0	0	0	0.000110387459984546
unclassified(100)	Otu052449	0.0000778513040093422	0	0	0	0
unclassified(100)	Otu052505	0	0	0.000109075043630017	0	0
unclassified(100)	Otu052506	0	0	0.000109075043630017	0	0
unclassified(100)	Otu052507	0	0	0.000109075043630017	0	0
unclassified(100)	Otu052509	0	0	0.000109075043630017	0	0
unclassified(100)	Otu052510	0	0	0.000109075043630017	0	0
unclassified(100)	Otu052516	0.0000778513040093422	0	0	0	0
unclassified(100)	Otu052517	0.0000778513040093422	0	0	0	0
unclassified(100)	Otu052519	0.0000778513040093422	0	0	0	0
unclassified(100)	Otu052523	0.0000778513040093422	0	0	0	0
unclassified(100)	Otu052538	0.0000778513040093422	0	0	0	0
unclassified(100)	Otu052543	0.0000778513040093422	0	0	0	0
unclassified(100)	Otu052546	0.0000778513040093422	0	0	0	0
unclassified(100)	Otu052554	0	0.000124968757810547	0	0	0
unclassified(100)	Otu052557	0	0.000124968757810547	0	0	0
unclassified(100)	Otu052561	0	0.000124968757810547	0	0	0
unclassified(100)	Otu052566	0	0	0	0.00012482836100362	0
unclassified(100)	Otu052569	0	0	0	0.00012482836100362	0
unclassified(100)	Otu052598	0.0000778513040093422	0	0	0	0
unclassified(100)	Otu052602	0.0000778513040093422	0	0	0	0

unclassified(100)	Otu052603	0.0000778513040093422	0	0	0	0
unclassified(100)	Otu052604	0.0000778513040093422	0	0	0	0
unclassified(100)	Otu052606	0	0.000124968757810547	0	0	0
unclassified(100)	Otu053155	0.0000778513040093422	0	0	0	0
unclassified(100)	Otu053159	0.0000778513040093422	0	0	0	0
unclassified(100)	Otu053161	0.0000778513040093422	0	0	0	0
unclassified(100)	Otu053162	0.0000778513040093422	0	0	0	0
unclassified(100)	Otu053169	0.0000778513040093422	0	0	0	0
unclassified(100)	Otu053181	0.0000778513040093422	0	0	0	0
unclassified(100)	Otu053182	0.0000778513040093422	0	0	0	0
unclassified(100)	Otu053191	0.0000778513040093422	0	0	0	0
unclassified(100)	Otu053192	0.0000778513040093422	0	0	0	0
unclassified(100)	Otu053197	0.0000778513040093422	0	0	0	0
unclassified(100)	Otu053198	0.0000778513040093422	0	0	0	0
unclassified(100)	Otu053199	0.0000778513040093422	0	0	0	0
unclassified(100)	Otu053238	0.0000778513040093422	0	0	0	0
unclassified(100)	Otu053744	0	0	0	0	0.000110387459984546
unclassified(100)	Otu053745	0	0	0	0	0.000110387459984546
unclassified(100)	Otu053767	0	0	0	0	0.000110387459984546
unclassified(100)	Otu053771	0	0	0	0	0.000110387459984546
unclassified(100)	Otu053772	0	0	0	0	0.000110387459984546
unclassified(100)	Otu053774	0	0	0	0	0.000110387459984546
unclassified(100)	Otu053779	0	0	0	0	0.000110387459984546
unclassified(100)	Otu053781	0	0	0	0	0.000110387459984546
unclassified(100)	Otu053823	0	0	0	0.00012482836100362	0
unclassified(100)	Otu053824	0	0	0	0.00012482836100362	0
unclassified(100)	Otu054166	0	0.000124968757810547	0	0	0
unclassified(100)	Otu054170	0	0	0.000109075043630017	0	0.000110387459984546
unclassified(100)	Otu054173	0.0000778513040093422	0	0	0	0
unclassified(100)	Otu054199	0	0	0.000109075043630017	0	0
unclassified(100)	Otu054201	0	0.000124968757810547	0	0	0
unclassified(100)	Otu054204	0	0	0.000109075043630017	0	0
unclassified(100)	Otu054207	0	0	0.000218150087260035	0	0

unclassified(100)	Otu059291	0.0000778513040093422	0	0	0	0
Aquabacterium(100)	Otu059295	0.0000778513040093422	0	0	0	0
Pelomonas(100)	Otu059449	0.0000778513040093422	0	0	0	0
unclassified(100)	Otu059452	0.0000778513040093422	0	0	0	0
unclassified(100)	Otu059458	0.0000778513040093422	0	0	0	0
unclassified(100)	Otu059462	0.0000778513040093422	0	0	0	0
unclassified(100)	Otu059503	0.0000778513040093422	0	0	0	0
Aquabacterium(100)	Otu059517	0.0000778513040093422	0	0	0	0
unclassified(100)	Otu059519	0.0000778513040093422	0	0	0	0
unclassified(100)	Otu059557	0.0000778513040093422	0	0	0	0
unclassified(100)	Otu059576	0.0000778513040093422	0	0	0	0
unclassified(100)	Otu059591	0.0000778513040093422	0	0	0	0
unclassified(100)	Otu059604	0.0000778513040093422	0	0	0	0
unclassified(100)	Otu059619	0.0000778513040093422	0	0	0	0
unclassified(100)	Otu059676	0.0000778513040093422	0	0	0	0
unclassified(100)	Otu059692	0.0000778513040093422	0	0	0	0
Aquabacterium(100)	Otu059703	0.0000778513040093422	0	0	0	0
unclassified(100)	Otu059711	0.0000778513040093422	0	0	0	0
unclassified(100)	Otu059712	0.0000778513040093422	0	0	0	0
Aquabacterium(100)	Otu059718	0.0000778513040093422	0	0	0	0
unclassified(100)	Otu059720	0.0000778513040093422	0	0	0	0
unclassified(100)	Otu064517	0.0000778513040093422	0	0	0	0
unclassified(100)	Otu065143	0.0000778513040093422	0	0	0	0
unclassified(100)	Otu065147	0.0000778513040093422	0	0	0	0
unclassified(100)	Otu065153	0.0000778513040093422	0	0	0	0
unclassified(100)	Otu065155	0.0000778513040093422	0	0	0	0
unclassified(100)	Otu065172	0.0000778513040093422	0	0	0	0
unclassified(100)	Otu065253	0.0000778513040093422	0	0	0	0
unclassified(100)	Otu065255	0.0000778513040093422	0	0	0	0
unclassified(100)	Otu065404	0.0000778513040093422	0	0	0	0
unclassified(100)	Otu065411	0.0000778513040093422	0	0	0	0
unclassified(100)	Otu065419	0.0000778513040093422	0	0	0	0
unclassified(100)	Otu065429	0.0000778513040093422	0	0	0	0

unclassified(100)	Otu065446	0.0000778513040093422	0	0	0	0
unclassified(100)	Otu065449	0.0000778513040093422	0	0	0	0
unclassified(100)	Otu065521	0.0000778513040093422	0	0	0	0
unclassified(100)	Otu065523	0.0000778513040093422	0	0	0	0
unclassified(100)	Otu065585	0.0000778513040093422	0	0	0	0
unclassified(100)	Otu065621	0.0000778513040093422	0	0	0	0
unclassified(100)	Otu065622	0.0000778513040093422	0	0	0	0
unclassified(100)	Otu065623	0.0000778513040093422	0	0	0	0
Pseudomonas(100)	Otu070303	0	0	0.000109075043630017	0	0
Pseudomonas(100)	Otu070311	0	0	0.000109075043630017	0	0
Cellvibrio(100)	Otu070320	0	0	0	0.00012482836100362	0
Pseudomonas(100)	Otu070325	0	0	0	0.00012482836100362	0
Pseudomonas(100)	Otu070341	0	0	0.000109075043630017	0	0
Pseudomonas(100)	Otu070346	0	0	0.000109075043630017	0	0
Pseudomonas(100)	Otu070368	0	0	0	0.00012482836100362	0
Pseudomonas(100)	Otu070380	0	0	0	0.00012482836100362	0
Pseudomonas(100)	Otu070386	0	0	0	0.00012482836100362	0
Pseudomonas(100)	Otu070392	0	0	0	0.00012482836100362	0
Pseudomonas(100)	Otu070403	0	0	0	0.00012482836100362	0
unclassified(100)	Otu070410	0	0	0	0.00012482836100362	0
Pseudomonas(100)	Otu070412	0	0	0	0.00012482836100362	0
Pseudomonas(100)	Otu071083	0	0.000124968757810547	0	0	0
Pseudomonas(100)	Otu071112	0	0	0	0	0.000110387459984546
Pseudomonas(100)	Otu071113	0	0	0	0	0.000110387459984546
Pseudomonas(100)	Otu071139	0	0	0	0.00012482836100362	0
Pseudomonas(100)	Otu071145	0	0	0	0.00012482836100362	0
Pseudomonas(100)	Otu071189	0	0	0.000109075043630017	0	0
unclassified(100)	Otu071201	0	0.000124968757810547	0	0	0
Pseudomonas(100)	Otu071210	0	0.000124968757810547	0	0	0
Pseudomonas(100)	Otu071215	0	0.000124968757810547	0	0	0
Pseudomonas(100)	Otu071284	0	0.000124968757810547	0	0	0
Pseudomonas(100)	Otu071316	0	0.000124968757810547	0	0	0
Pseudomonas(100)	Otu071324	0	0.000124968757810547	0	0	0

Pseudomonas(100)	Otu071326	0	0.000124968757810547	0	0	0
Pseudomonas(100)	Otu071328	0	0.000124968757810547	0	0	0
Pseudomonas(100)	Otu071330	0	0.000124968757810547	0	0	0
Pseudomonas(100)	Otu071333	0	0.000124968757810547	0	0	0
Pseudomonas(100)	Otu071345	0	0.000124968757810547	0	0	0
Pseudomonas(100)	Otu071347	0	0.000124968757810547	0	0	0
Pseudomonas(100)	Otu071352	0	0.000124968757810547	0	0	0
Pseudomonas(100)	Otu071367	0	0.000124968757810547	0	0	0
unclassified(100)	Otu071373	0	0.000124968757810547	0	0	0
Pseudomonas(100)	Otu071389	0	0	0	0.00012482836100362	0
Pseudomonas(100)	Otu071417	0	0	0	0	0.000110387459984546
Pseudomonas(100)	Otu071420	0	0	0	0	0.000110387459984546
Pseudomonas(100)	Otu071430	0	0	0	0	0.000110387459984546
Pseudomonas(100)	Otu071435	0	0	0	0.00012482836100362	0
Pseudomonas(100)	Otu071448	0	0	0	0	0.000110387459984546
Pseudomonas(100)	Otu071491	0	0.000124968757810547	0	0	0
Pseudomonas(100)	Otu071496	0	0	0	0	0.000110387459984546
uncultured(100)	Otu071497	0	0	0	0	0.000110387459984546
unclassified(100)	Otu071502	0	0	0	0	0.000110387459984546
Pseudomonas(100)	Otu071503	0	0	0	0	0.000110387459984546
Pseudomonas(100)	Otu071505	0	0	0	0	0.000110387459984546
unclassified(100)	Otu071517	0	0	0	0	0.000110387459984546
Pseudomonas(100)	Otu071582	0	0.000124968757810547	0	0	0
Pseudomonas(100)	Otu071653	0	0	0	0.00012482836100362	0
Pseudomonas(100)	Otu071660	0	0	0	0.00012482836100362	0
Pseudomonas(100)	Otu071685	0	0	0	0.00012482836100362	0
uncultured(100)	Otu071697	0	0	0.000109075043630017	0	0
Pseudomonas(100)	Otu071700	0	0	0.000109075043630017	0	0
Pseudomonas(100)	Otu071701	0	0	0.000109075043630017	0	0
Pseudomonas(100)	Otu071702	0	0	0.000109075043630017	0	0
Pseudomonas(100)	Otu071800	0	0.000124968757810547	0	0	0
Pseudomonas(100)	Otu071802	0	0.000124968757810547	0	0	0
Pseudomonas(100)	Otu071815	0	0	0.000109075043630017	0	0

Pseudomonas(100)	Otu071817	0	0.000124968757810547	0	0	0
Pseudomonas(100)	Otu071837	0	0.000124968757810547	0	0	0
Pseudomonas(100)	Otu071853	0	0	0.000109075043630017	0	0
Pseudomonas(100)	Otu071857	0	0	0.000109075043630017	0	0
Pseudomonas(100)	Otu071863	0	0.000124968757810547	0	0	0
Pseudomonas(100)	Otu071882	0	0.000124968757810547	0	0	0
Pseudomonas(100)	Otu071978	0	0.000124968757810547	0	0	0
Pseudomonas(100)	Otu076133	0	0	0	0	0.000110387459984546
uncultured(100)	Otu076217	0.0000778513040093422	0	0	0	0
uncultured(100)	Otu076219	0.0000778513040093422	0	0	0	0
uncultured(100)	Otu076236	0.0000778513040093422	0	0	0	0
uncultured(100)	Otu077778	0.0000778513040093422	0	0	0	0
unclassified(100)	Otu079862	0	0	0	0	0.000110387459984546
unclassified(100)	Otu079878	0	0	0	0	0.000110387459984546
unclassified(100)	Otu079879	0	0	0	0	0.000110387459984546
unclassified(100)	Otu079892	0	0	0	0.00012482836100362	0
unclassified(100)	Otu079960	0	0	0	0	0.000110387459984546
unclassified(100)	Otu080010	0	0	0	0	0.000110387459984546
unclassified(100)	Otu080035	0	0	0	0.00012482836100362	0
unclassified(100)	Otu080092	0	0.000124968757810547	0	0	0
unclassified(100)	Otu080096	0	0.000124968757810547	0	0	0
unclassified(100)	Otu080099	0	0.000124968757810547	0	0	0
unclassified(100)	Otu080134	0	0	0	0.00012482836100362	0
unclassified(100)	Otu080136	0	0	0	0.00012482836100362	0
unclassified(100)	Otu080209	0	0	0	0.00012482836100362	0
unclassified(100)	Otu080218	0	0	0	0	0.000993487139860912
unclassified(100)	Otu080415	0.0000778513040093422	0	0	0	0
unclassified(100)	Otu080511	0	0.000124968757810547	0	0	0
unclassified(100)	Otu080616	0	0	0	0.00012482836100362	0
unclassified(100)	Otu080639	0	0	0	0.00012482836100362	0
unclassified(100)	Otu080643	0	0	0	0.00012482836100362	0
unclassified(100)	Otu080646	0	0	0	0.00012482836100362	0
unclassified(100)	Otu080665	0	0	0	0	0.000110387459984546

unclassified(100)	Otu080666	0	0	0	0	0.000110387459984546
unclassified(100)	Otu080672	0	0	0	0	0.000110387459984546
unclassified(100)	Otu080682	0	0	0	0.00012482836100362	0
unclassified(100)	Otu080684	0	0	0	0.00012482836100362	0
unclassified(100)	Otu080686	0	0	0	0.00012482836100362	0
unclassified(100)	Otu080692	0	0	0	0	0.000110387459984546
unclassified(100)	Otu080693	0	0	0	0	0.000110387459984546
unclassified(100)	Otu080694	0	0	0	0	0.000110387459984546
unclassified(100)	Otu080703	0	0	0	0	0.000110387459984546
unclassified(100)	Otu080707	0	0	0	0	0.000110387459984546
unclassified(100)	Otu080708	0	0	0	0	0.000110387459984546
unclassified(100)	Otu080717	0	0	0	0	0.000110387459984546
unclassified(100)	Otu080720	0	0	0	0	0.000110387459984546
unclassified(100)	Otu080760	0.0000778513040093422	0	0	0	0
unclassified(100)	Otu080763	0.0000778513040093422	0	0	0	0
unclassified(100)	Otu080764	0.0000778513040093422	0	0	0	0
unclassified(100)	Otu080792	0	0	0	0.00012482836100362	0
unclassified(100)	Otu080803	0	0	0	0.00012482836100362	0
unclassified(100)	Otu080806	0	0	0	0.00012482836100362	0
unclassified(100)	Otu084290	0	0.000124968757810547	0	0	0
unclassified(100)	Otu084339	0	0.000124968757810547	0	0	0
unclassified(100)	Otu084360	0	0.000124968757810547	0	0	0
unclassified(100)	Otu084365	0	0.000124968757810547	0	0	0
unclassified(100)	Otu084381	0	0.000124968757810547	0	0	0
unclassified(100)	Otu084391	0	0.000124968757810547	0	0	0
unclassified(100)	Otu084530	0	0	0	0.00012482836100362	0
unclassified(100)	Otu084533	0	0	0	0.00012482836100362	0
unclassified(100)	Otu084535	0	0	0	0.00012482836100362	0
unclassified(100)	Otu084541	0	0	0	0.00012482836100362	0
unclassified(100)	Otu084547	0	0	0	0.00012482836100362	0
unclassified(100)	Otu084548	0	0	0	0.00012482836100362	0
unclassified(100)	Otu084559	0	0	0.000109075043630017	0	0
unclassified(100)	Otu084562	0	0	0.000109075043630017	0	0

unclassified(100)	Otu084563	0	0	0.000109075043630017	0	0
unclassified(100)	Otu084564	0	0	0.000109075043630017	0	0
unclassified(100)	Otu084566	0	0	0.000109075043630017	0	0
unclassified(100)	Otu084572	0	0	0.000109075043630017	0	0
unclassified(100)	Otu084573	0	0	0.000109075043630017	0	0
unclassified(100)	Otu084575	0	0	0	0.00012482836100362	0
unclassified(100)	Otu084582	0	0	0.000109075043630017	0	0
unclassified(100)	Otu084585	0	0	0	0.00012482836100362	0
unclassified(100)	Otu084596	0	0	0	0.00012482836100362	0
unclassified(100)	Otu084612	0	0	0	0.00012482836100362	0
unclassified(100)	Otu084615	0	0	0	0.00012482836100362	0
unclassified(100)	Otu084618	0	0	0	0.00012482836100362	0
unclassified(100)	Otu084630	0	0	0.000109075043630017	0	0
unclassified(100)	Otu084632	0	0	0	0.00012482836100362	0
unclassified(100)	Otu084636	0	0	0	0.00012482836100362	0
unclassified(100)	Otu084639	0	0	0	0.00012482836100362	0
unclassified(100)	Otu084647	0	0.000124968757810547	0	0	0
unclassified(100)	Otu084675	0	0.000124968757810547	0	0	0
unclassified(100)	Otu084681	0	0.000124968757810547	0	0	0
unclassified(100)	Otu084684	0	0.000124968757810547	0	0	0
unclassified(100)	Otu084699	0	0.000124968757810547	0	0	0
unclassified(100)	Otu084704	0	0.000124968757810547	0	0	0
unclassified(100)	Otu084713	0	0	0.000109075043630017	0	0
unclassified(100)	Otu084758	0	0	0.000109075043630017	0	0
unclassified(100)	Otu084766	0	0	0.000109075043630017	0	0
Methylobacterium(100)	Otu086055	0.0000778513040093422	0	0	0	0
Methylobacterium(100)	Otu086117	0.0000778513040093422	0	0	0	0
Methylobacterium(100)	Otu086126	0.0000778513040093422	0	0	0	0
Methylobacterium(100)	Otu086128	0.0000778513040093422	0	0	0	0
Methylobacterium(100)	Otu086182	0.0000778513040093422	0	0	0	0
Methylobacterium(100)	Otu086212	0.0000778513040093422	0	0	0	0
Methylobacterium(100)	Otu086220	0.0000778513040093422	0	0	0	0
Methylobacterium(100)	Otu086856	0	0	0	0.00012482836100362	0

Methylobacterium(100)	Otu086881	0	0	0	0	0.000110387459984546
Methylobacterium(100)	Otu086885	0	0	0	0	0.000110387459984546
Methylobacterium(100)	Otu086889	0	0.000124968757810547	0	0	0
Methylobacterium(100)	Otu086907	0.0000778513040093422	0	0	0	0
Methylobacterium(100)	Otu086912	0	0	0	0.00012482836100362	0
Methylobacterium(100)	Otu086923	0	0	0	0	0.000110387459984546
Methylobacterium(100)	Otu086926	0	0	0	0	0.000110387459984546
Methylobacterium(100)	Otu086991	0	0	0.000109075043630017	0	0
Methylobacterium(100)	Otu087015	0	0	0.000109075043630017	0	0
Methylobacterium(100)	Otu087020	0	0	0.000109075043630017	0	0
Methylobacterium(100)	Otu087023	0	0	0.000109075043630017	0	0
Methylobacterium(100)	Otu087026	0	0	0.000109075043630017	0	0
Methylobacterium(100)	Otu087027	0	0	0.000109075043630017	0	0
Methylobacterium(100)	Otu087028	0	0	0.000109075043630017	0	0
Methylobacterium(100)	Otu087031	0	0	0.000109075043630017	0	0
Methylobacterium(100)	Otu087033	0	0	0.000218150087260035	0	0
Methylobacterium(100)	Otu087205	0	0	0	0	0.000110387459984546
Methylobacterium(100)	Otu087220	0.0000778513040093422	0	0	0	0
Methylobacterium(100)	Otu087231	0.0000778513040093422	0	0	0	0
Methylobacterium(100)	Otu087232	0.0000778513040093422	0	0	0	0
Methylobacterium(100)	Otu087235	0.0000778513040093422	0	0	0	0
Methylobacterium(100)	Otu087236	0.0000778513040093422	0	0	0	0
Methylobacterium(100)	Otu087241	0.0000778513040093422	0	0	0	0
Methylobacterium(100)	Otu087291	0.0000778513040093422	0	0	0	0
Methylobacterium(100)	Otu087317	0.0000778513040093422	0	0	0	0
Methylobacterium(100)	Otu087336	0.0000778513040093422	0	0	0	0
Methylobacterium(100)	Otu087355	0.0000778513040093422	0	0	0	0
Methylobacterium(100)	Otu087397	0.0000778513040093422	0	0	0	0
Methylobacterium(100)	Otu087404	0.0000778513040093422	0	0	0	0
Methylobacterium(100)	Otu087416	0.0000778513040093422	0	0	0	0
Methylobacterium(100)	Otu087425	0.0000778513040093422	0	0	0	0
Methylobacterium(100)	Otu087464	0.0000778513040093422	0	0	0	0
Methylobacterium(100)	Otu087469	0	0	0.000109075043630017	0	0

Methylobacterium(100)	Otu087474	0.0000778513040093422	0	0	0	0
Methylobacterium(100)	Otu087476	0.0000778513040093422	0	0	0	0
Methylobacterium(100)	Otu087478	0.0000778513040093422	0	0	0	0
Methylobacterium(100)	Otu087483	0.0000778513040093422	0	0	0	0
Methylobacterium(100)	Otu087484	0.0000778513040093422	0	0	0	0
Methylobacterium(100)	Otu087488	0.0000778513040093422	0	0	0	0
Methylobacterium(100)	Otu087497	0.0000778513040093422	0	0	0	0
Methylobacterium(100)	Otu087517	0	0.000124968757810547	0	0	0
Methylobacterium(100)	Otu087522	0	0.000124968757810547	0	0	0
Methylobacterium(100)	Otu087528	0	0	0	0	0.000110387459984546
Methylobacterium(100)	Otu087529	0	0	0	0	0.000110387459984546
Methylobacterium(100)	Otu087532	0	0	0	0	0.000110387459984546
Methylobacterium(100)	Otu087534	0	0	0	0	0.000110387459984546
Methylobacterium(100)	Otu087536	0	0	0	0.00012482836100362	0
Methylobacterium(100)	Otu087546	0	0	0	0	0.000110387459984546
Methylobacterium(100)	Otu087553	0	0	0	0	0.000110387459984546
Methylobacterium(100)	Otu087575	0	0	0.000109075043630017	0	0
Methylobacterium(100)	Otu087582	0	0.000124968757810547	0	0	0
Methylobacterium(100)	Otu087585	0	0.000124968757810547	0	0	0
Methylobacterium(100)	Otu087627	0.0000778513040093422	0	0	0	0
Methylobacterium(100)	Otu087658	0.0000778513040093422	0	0	0	0
Methylobacterium(100)	Otu087692	0.0000778513040093422	0	0	0	0
Methylobacterium(100)	Otu087699	0.0000778513040093422	0	0	0	0
Novosphingobium(100)	Otu087802	0	0	0	0	0.000110387459984546
Sphingobium(100)	Otu087855	0	0	0	0	0.000110387459984546
unclassified(100)	Otu087858	0	0	0	0	0.000110387459984546
Sphingobium(100)	Otu087939	0	0	0	0	0.000110387459984546
Sphingopyxis(100)	Otu087953	0	0	0	0	0.000110387459984546
Sphingomonas(100)	Otu088373	0	0	0	0	0.000110387459984546
Novosphingobium(100)	Otu088378	0	0	0	0	0.000110387459984546
Aeromonas(100)	Otu088695	0	0	0	0.00012482836100362	0
Aeromonas(100)	Otu088698	0	0	0	0.00012482836100362	0
Aeromonas(100)	Otu088750	0.0000778513040093422	0	0	0	0

Aeromonas(100)	Otu088798	0	0	0	0	0.000110387459984546
Aeromonas(100)	Otu088833	0	0	0	0.00012482836100362	0
Aeromonas(100)	Otu088848	0	0.000124968757810547	0	0	0
Aeromonas(100)	Otu088853	0	0	0	0.00012482836100362	0
Aeromonas(100)	Otu088856	0	0.000124968757810547	0	0	0
Aeromonas(100)	Otu088865	0	0	0	0.00012482836100362	0
Aeromonas(100)	Otu088895	0.0000778513040093422	0	0	0	0
Aeromonas(100)	Otu088897	0	0	0.000109075043630017	0	0
Aeromonas(100)	Otu088916	0	0	0	0.00012482836100362	0
Aeromonas(100)	Otu088982	0	0	0	0.00012482836100362	0
Aeromonas(100)	Otu089063	0	0	0	0	0.000110387459984546
Aeromonas(100)	Otu089085	0	0	0	0.00012482836100362	0
Aeromonas(100)	Otu089093	0	0	0	0	0.000110387459984546
Aeromonas(100)	Otu089112	0	0.000124968757810547	0	0	0
Aeromonas(100)	Otu089140	0	0.000124968757810547	0	0	0
Aeromonas(100)	Otu089155	0	0	0	0.00012482836100362	0
Aeromonas(100)	Otu089317	0	0.000124968757810547	0	0	0
Aeromonas(100)	Otu089417	0	0.000124968757810547	0	0	0
Aeromonas(100)	Otu089426	0	0	0	0.00012482836100362	0
Aeromonas(100)	Otu089586	0	0	0	0	0.000110387459984546
Enterococcus(100)	Otu089837	0	0.000124968757810547	0	0	0
unclassified(100)	Otu089949	0	0	0	0	0.000110387459984546
unclassified(100)	Otu089960	0	0	0.000109075043630017	0	0
unclassified(100)	Otu089964	0	0	0	0.00012482836100362	0
unclassified(100)	Otu089968	0	0	0	0	0.000110387459984546
unclassified(100)	Otu089969	0	0	0	0	0.000110387459984546
unclassified(100)	Otu090050	0	0.000124968757810547	0	0	0
unclassified(100)	Otu090063	0.0000778513040093422	0	0	0	0
Enterococcus(100)	Otu090498	0	0	0	0	0.000110387459984546
Enterococcus(100)	Otu090519	0	0	0.000109075043630017	0	0
Enterococcus(100)	Otu090521	0	0.000124968757810547	0	0	0
Deinococcus(100)	Otu090733	0	0.000249937515621095	0	0	0.000220774919969092
unclassified(100)	Otu090881	0	0	0	0	0.000110387459984546

unclassified(100)	Otu090882	0	0	0	0	0.00077271221989182
unclassified(100)	Otu090892	0	0	0	0.00024965672200724	0
unclassified(100)	Otu090948	0	0	0	0	0.000220774919969092
unclassified(100)	Otu090996	0	0	0.000109075043630017	0	0
uncultured_bacterium(100)	Otu091018	0	0	0.000545375218150087	0	0
uncultured_bacterium(100)	Otu091019	0	0	0.000109075043630017	0	0
unclassified(100)	Otu091079	0	0.000124968757810547	0	0	0
unclassified(100)	Otu091119	0	0	0.000109075043630017	0	0
Caedibacter(100)	Otu091172	0.0000778513040093422	0	0	0	0
Aquicella(100)	Otu091235	0	0	0	0	0.000662324759907275
Caedibacter(100)	Otu091242	0	0.000124968757810547	0	0	0
Caedibacter(100)	Otu091247	0.0000778513040093422	0	0	0	0
Aquicella(100)	Otu091515	0	0.000124968757810547	0	0	0
Coxiella(100)	Otu091553	0	0.000624843789052737	0	0	0
unclassified(100)	Otu091658	0	0	0.000109075043630017	0	0
unclassified(100)	Otu091661	0	0	0.000109075043630017	0	0
unclassified(100)	Otu091664	0	0	0.000109075043630017	0	0
unclassified(100)	Otu091700	0	0.000124968757810547	0	0	0
unclassified(100)	Otu091727	0	0	0	0	0.000110387459984546
unclassified(100)	Otu091728	0	0	0.000109075043630017	0	0
unclassified(100)	Otu091730	0	0	0.000109075043630017	0	0
unclassified(100)	Otu091732	0	0	0	0	0.000110387459984546
unclassified(100)	Otu091733	0	0	0	0	0.000110387459984546
unclassified(100)	Otu091737	0	0	0	0	0.000110387459984546
unclassified(100)	Otu091740	0	0	0.000109075043630017	0	0
unclassified(100)	Otu091744	0	0	0.000109075043630017	0	0
unclassified(100)	Otu091747	0	0	0.000109075043630017	0	0
unclassified(100)	Otu091750	0	0	0.000109075043630017	0	0
unclassified(100)	Otu091751	0	0	0.000109075043630017	0	0
unclassified(100)	Otu091752	0	0	0.000109075043630017	0	0
unclassified(100)	Otu091775	0	0.000124968757810547	0	0	0
unclassified(100)	Otu091817	0	0.000124968757810547	0	0	0
unclassified(100)	Otu091858	0.0000778513040093422	0	0	0	0

unclassified(100)	Otu091859	0.0000778513040093422	0	0	0	0
unclassified(100)	Otu092034	0	0	0	0	0.000110387459984546
unclassified(100)	Otu092035	0	0	0	0	0.000110387459984546
unclassified(100)	Otu092036	0	0	0.000109075043630017	0	0
unclassified(100)	Otu092039	0	0	0.000109075043630017	0	0
unclassified(100)	Otu092040	0	0	0.000109075043630017	0	0
unclassified(100)	Otu092042	0	0	0.000109075043630017	0	0
unclassified(100)	Otu092044	0	0	0.000109075043630017	0	0
unclassified(100)	Otu092048	0	0	0.000109075043630017	0	0
unclassified(100)	Otu092052	0	0	0.000109075043630017	0	0
unclassified(100)	Otu092054	0	0	0.000109075043630017	0	0
unclassified(100)	Otu092056	0	0	0.000109075043630017	0	0
unclassified(100)	Otu092263	0.0000778513040093422	0	0	0	0
unclassified(100)	Otu092266	0.0000778513040093422	0	0	0	0
unclassified(100)	Otu092268	0.0000778513040093422	0	0	0	0
unclassified(100)	Otu092291	0.0000778513040093422	0	0	0	0
unclassified(100)	Otu092294	0.0000778513040093422	0	0	0	0
unclassified(100)	Otu092296	0.0000778513040093422	0	0	0	0
unclassified(100)	Otu092297	0.0000778513040093422	0	0	0	0
unclassified(100)	Otu092325	0.0000778513040093422	0	0	0	0
unclassified(100)	Otu092340	0.0000778513040093422	0	0	0	0
unclassified(100)	Otu092341	0.0000778513040093422	0	0	0	0
unclassified(100)	Otu092349	0.0000778513040093422	0	0	0	0
unclassified(100)	Otu092351	0.0000778513040093422	0	0	0	0
unclassified(100)	Otu092352	0.0000778513040093422	0	0	0	0
unclassified(100)	Otu092356	0.0000778513040093422	0	0	0	0
unclassified(100)	Otu092357	0.0000778513040093422	0	0	0	0
unclassified(100)	Otu092360	0.0000778513040093422	0	0	0	0
unclassified(100)	Otu092363	0.0000778513040093422	0	0	0	0
unclassified(100)	Otu092374	0	0	0	0	0.000110387459984546
unclassified(100)	Otu092377	0	0.000124968757810547	0	0	0
unclassified(100)	Otu092386	0	0	0.000109075043630017	0	0
unclassified(100)	Otu092387	0	0	0.000109075043630017	0	0

unclassified(100)	Otu092392	0	0	0.000109075043630017	0	0
unclassified(100)	Otu092394	0	0	0.000109075043630017	0	0
unclassified(100)	Otu092395	0	0	0	0	0.000110387459984546
unclassified(100)	Otu092396	0	0	0	0	0.000110387459984546
unclassified(100)	Otu092400	0	0	0	0.00012482836100362	0
unclassified(100)	Otu092412	0	0	0	0	0.000110387459984546
unclassified(100)	Otu092415	0	0	0	0	0.000110387459984546
unclassified(100)	Otu092416	0	0	0.000109075043630017	0	0
unclassified(100)	Otu092418	0	0	0.000109075043630017	0	0
unclassified(100)	Otu092419	0	0	0.000109075043630017	0	0
unclassified(100)	Otu092421	0	0	0.000109075043630017	0	0
unclassified(100)	Otu092423	0	0	0	0	0.000110387459984546
unclassified(100)	Otu092424	0	0	0	0	0.000110387459984546
unclassified(100)	Otu092426	0	0	0.000109075043630017	0	0
unclassified(100)	Otu092427	0	0	0.000109075043630017	0	0
unclassified(100)	Otu092429	0	0	0	0	0.000110387459984546
unclassified(100)	Otu092438	0	0	0	0	0.000110387459984546
unclassified(100)	Otu092439	0	0	0	0	0.000110387459984546
unclassified(100)	Otu092440	0	0	0	0	0.000110387459984546
unclassified(100)	Otu092442	0	0	0	0	0.000110387459984546
unclassified(100)	Otu092451	0	0.000124968757810547	0	0	0
unclassified(100)	Otu092460	0.0000778513040093422	0	0	0	0
unclassified(100)	Otu092462	0.0000778513040093422	0	0	0	0
unclassified(100)	Otu092467	0	0	0	0.00012482836100362	0
unclassified(100)	Otu092472	0	0	0	0.00012482836100362	0
unclassified(100)	Otu092484	0	0	0	0	0.000110387459984546
unclassified(100)	Otu092695	0	0	0	0.00012482836100362	0
Paracoccus(100)	Otu092702	0	0	0	0.00012482836100362	0
unclassified(100)	Otu092704	0	0.000124968757810547	0	0	0
Paracoccus(100)	Otu092716	0	0	0	0.00012482836100362	0
unclassified(100)	Otu092717	0	0.000124968757810547	0	0	0
Paracoccus(100)	Otu092724	0	0.000124968757810547	0	0	0
unclassified(100)	Otu092725	0	0	0	0.00012482836100362	0

Paracoccus(100)	Otu092735	0	0	0	0.00012482836100362	0
Paracoccus(100)	Otu092807	0	0	0	0.00012482836100362	0
Rubellimicrobium(100)	Otu092817	0	0	0	0	0.000110387459984546
Rubellimicrobium(100)	Otu093016	0	0	0	0.00149794033204344	0
Rubellimicrobium(100)	Otu093019	0	0	0	0.00049931344401448	0.000110387459984546
unclassified(100)	Otu093029	0	0	0	0	0.000110387459984546
Paracoccus(100)	Otu093032	0	0	0	0	0.000110387459984546
Paracoccus(100)	Otu093039	0	0.000124968757810547	0	0	0
Sphingomonas(100)	Otu097522	0.0000778513040093422	0	0	0	0
Sphingomonas(100)	Otu097524	0.0000778513040093422	0	0	0	0
Novosphingobium(100)	Otu097525	0.0000778513040093422	0	0	0	0
Sphingobium(100)	Otu097536	0.0000778513040093422	0	0	0	0
Novosphingobium(100)	Otu097541	0	0.000124968757810547	0	0	0
Novosphingobium(100)	Otu097545	0.0000778513040093422	0	0	0	0
Sphingobium(100)	Otu097547	0.0000778513040093422	0	0	0	0
unclassified(100)	Otu097568	0.0000778513040093422	0	0	0	0
unclassified(100)	Otu097580	0.0000778513040093422	0	0	0	0
Sphingomonas(100)	Otu097583	0.0000778513040093422	0	0	0	0
Sphingomonas(100)	Otu097591	0.0000778513040093422	0	0	0	0
Sphingobium(100)	Otu097594	0.0000778513040093422	0	0	0	0
unclassified(100)	Otu097597	0	0.000124968757810547	0	0	0
Novosphingobium(100)	Otu097614	0	0.000124968757810547	0	0	0
Novosphingobium(100)	Otu097619	0.0000778513040093422	0	0	0	0
Novosphingobium(100)	Otu097623	0.0000778513040093422	0	0	0	0
Novosphingobium(100)	Otu097648	0.0000778513040093422	0	0	0	0
Novosphingobium(100)	Otu097649	0.0000778513040093422	0	0	0	0
Novosphingobium(100)	Otu097661	0.0000778513040093422	0	0	0	0
unclassified(100)	Otu097663	0.0000778513040093422	0	0	0	0
Novosphingobium(100)	Otu097673	0.0000778513040093422	0	0	0	0
Sphingomonas(100)	Otu097690	0.000155702608018684	0	0	0	0
Novosphingobium(100)	Otu097694	0.0000778513040093422	0	0	0	0
unclassified(100)	Otu097706	0.0000778513040093422	0	0	0	0
Novosphingobium(100)	Otu097726	0.0000778513040093422	0	0	0	0

Sphingomonas(100)	Otu097731	0.0000778513040093422	0	0	0	0
Novosphingobium(100)	Otu097734	0.0000778513040093422	0	0	0	0
Novosphingobium(100)	Otu097735	0.0000778513040093422	0	0	0	0
Sphingomonas(100)	Otu097746	0.0000778513040093422	0	0	0	0
Sphingomonas(100)	Otu097751	0.0000778513040093422	0	0	0	0
Sphingobium(100)	Otu097756	0.0000778513040093422	0	0	0	0
unclassified(100)	Otu097759	0.0000778513040093422	0	0	0	0
unclassified(100)	Otu097768	0.0000778513040093422	0	0	0	0
Novosphingobium(100)	Otu097773	0.0000778513040093422	0	0	0	0
unclassified(100)	Otu097781	0.0000778513040093422	0	0	0	0
unclassified(100)	Otu097787	0	0	0.000109075043630017	0	0
unclassified(100)	Otu097789	0.0000778513040093422	0	0	0	0
Novosphingobium(100)	Otu097794	0.0000778513040093422	0	0	0	0
unclassified(100)	Otu097800	0	0	0.000109075043630017	0	0
Sphingobium(100)	Otu097801	0	0	0.000109075043630017	0	0
unclassified(100)	Otu097805	0.0000778513040093422	0	0	0	0
Sphingomonas(100)	Otu097822	0	0	0.000109075043630017	0	0
unclassified(100)	Otu097824	0.0000778513040093422	0	0	0	0
unclassified(100)	Otu097831	0.0000778513040093422	0	0	0	0
unclassified(100)	Otu097836	0.0000778513040093422	0	0	0	0
Novosphingobium(100)	Otu097847	0.0000778513040093422	0	0	0	0
Novosphingobium(100)	Otu097848	0.0000778513040093422	0	0	0	0
unclassified(100)	Otu097861	0.0000778513040093422	0	0	0	0
unclassified(100)	Otu097871	0.0000778513040093422	0	0	0	0
Novosphingobium(100)	Otu097886	0.0000778513040093422	0	0	0	0
Sphingomonas(100)	Otu097892	0.0000778513040093422	0	0	0	0
unclassified(100)	Otu097914	0.0000778513040093422	0	0	0	0
Novosphingobium(100)	Otu097916	0.0000778513040093422	0	0	0	0
Novosphingobium(100)	Otu097917	0	0	0.000109075043630017	0	0
Novosphingobium(100)	Otu097923	0.0000778513040093422	0	0	0	0
Novosphingobium(100)	Otu097925	0.0000778513040093422	0	0	0	0
Novosphingobium(100)	Otu097942	0.0000778513040093422	0	0	0	0
Novosphingobium(100)	Otu097951	0.0000778513040093422	0	0	0	0

Novosphingobium(100)	Otu097954	0	0	0.000109075043630017	0	0
unclassified(100)	Otu097957	0.0000778513040093422	0	0	0	0
Sphingomonas(100)	Otu097960	0.0000778513040093422	0	0	0	0
unclassified(100)	Otu097961	0.0000778513040093422	0	0	0	0
Novosphingobium(100)	Otu097969	0.0000778513040093422	0	0	0	0
unclassified(100)	Otu097984	0.0000778513040093422	0	0	0	0
unclassified(100)	Otu097989	0.0000778513040093422	0	0	0	0
unclassified(100)	Otu097991	0.0000778513040093422	0	0	0	0
Novosphingobium(100)	Otu098000	0.0000778513040093422	0	0	0	0
unclassified(100)	Otu098011	0.0000778513040093422	0	0	0	0
Sphingobium(100)	Otu098012	0	0	0.000109075043630017	0	0
Novosphingobium(100)	Otu098018	0.0000778513040093422	0	0	0	0
Novosphingobium(100)	Otu098027	0.0000778513040093422	0	0	0	0
Novosphingobium(100)	Otu098028	0.0000778513040093422	0	0	0	0
Novosphingobium(100)	Otu098033	0	0	0.000109075043630017	0	0
Sphingopyxis(100)	Otu098041	0	0	0.000109075043630017	0	0
Novosphingobium(100)	Otu098062	0.0000778513040093422	0	0	0	0
Novosphingobium(100)	Otu098067	0	0	0.000109075043630017	0	0
unclassified(100)	Otu098078	0.0000778513040093422	0	0	0	0
unclassified(100)	Otu098079	0	0	0.000109075043630017	0	0
unclassified(100)	Otu098085	0.0000778513040093422	0	0	0	0
Novosphingobium(100)	Otu098097	0.0000778513040093422	0	0	0	0
Novosphingobium(100)	Otu098108	0.0000778513040093422	0	0	0	0
Sphingopyxis(100)	Otu098132	0	0	0.000109075043630017	0	0
Novosphingobium(100)	Otu098157	0	0	0.000109075043630017	0	0
unclassified(100)	Otu098163	0.0000778513040093422	0	0	0	0
Sphingomonas(100)	Otu098203	0	0	0.000109075043630017	0	0
Novosphingobium(100)	Otu098214	0.0000778513040093422	0	0	0	0
Novosphingobium(100)	Otu098216	0	0	0.000109075043630017	0	0
Sphingobium(100)	Otu098234	0	0	0.000109075043630017	0	0
Sphingobium(100)	Otu098241	0	0	0.000109075043630017	0	0
Sphingobium(100)	Otu098261	0.0000778513040093422	0	0	0	0
Novosphingobium(100)	Otu098267	0.0000778513040093422	0	0	0	0

Novosphingobium(100)	Otu098272	0.0000778513040093422	0	0	0	0
Novosphingobium(100)	Otu098275	0.0000778513040093422	0	0	0	0
Novosphingobium(100)	Otu098280	0.0000778513040093422	0	0	0	0
Novosphingobium(100)	Otu098287	0.0000778513040093422	0	0	0	0
unclassified(100)	Otu098295	0.0000778513040093422	0	0	0	0
Novosphingobium(100)	Otu098335	0.0000778513040093422	0	0	0	0
Sphingobium(100)	Otu098358	0.0000778513040093422	0	0	0	0
Novosphingobium(100)	Otu098359	0.0000778513040093422	0	0	0	0
unclassified(100)	Otu098362	0.0000778513040093422	0	0	0	0
Sphingomonas(100)	Otu098363	0.0000778513040093422	0	0	0	0
Novosphingobium(100)	Otu098375	0.0000778513040093422	0	0	0	0
Novosphingobium(100)	Otu098384	0.0000778513040093422	0	0	0	0
unclassified(100)	Otu098394	0.0000778513040093422	0	0	0	0
Sphingobium(100)	Otu098400	0	0	0.000109075043630017	0	0
unclassified(100)	Otu098401	0	0	0.000109075043630017	0	0
Sphingobium(100)	Otu098404	0	0	0.000109075043630017	0	0
Novosphingobium(100)	Otu098412	0.0000778513040093422	0	0	0	0
Novosphingobium(100)	Otu098425	0.0000778513040093422	0	0	0	0
Novosphingobium(100)	Otu098431	0.0000778513040093422	0	0	0	0
Novosphingobium(100)	Otu098455	0	0	0.000109075043630017	0	0
unclassified(100)	Otu098464	0.0000778513040093422	0	0	0	0
Novosphingobium(100)	Otu098468	0.0000778513040093422	0	0	0	0
unclassified(100)	Otu098485	0	0	0.000109075043630017	0	0
unclassified(100)	Otu098492	0.0000778513040093422	0	0	0	0
Novosphingobium(100)	Otu098525	0	0	0	0.00012482836100362	0
Sphingomonas(100)	Otu098556	0	0	0	0	0.000110387459984546
Novosphingobium(100)	Otu098577	0	0	0	0.00012482836100362	0
Sphingomonas(100)	Otu098650	0	0	0	0	0.000110387459984546
unclassified(100)	Otu098690	0	0	0	0.00012482836100362	0
Sphingobium(100)	Otu098710	0	0	0	0	0.000110387459984546
Sphingomonas(100)	Otu098742	0	0	0	0.00012482836100362	0
Sphingomonas(100)	Otu098743	0	0	0	0.00012482836100362	0
unclassified(100)	Otu098761	0	0	0	0.00012482836100362	0

Sphingobium(100)	Otu098762	0	0	0	0	0.000110387459984546
Sphingobium(100)	Otu098816	0	0	0	0	0.000110387459984546
unclassified(100)	Otu098822	0	0	0	0	0.000110387459984546
Sphingomonas(100)	Otu098899	0	0	0.000109075043630017	0	0
Sphingomonas(100)	Otu098911	0	0	0	0.00012482836100362	0
Sphingobium(100)	Otu098914	0	0	0	0.00012482836100362	0
Novosphingobium(100)	Otu098967	0	0	0.000109075043630017	0	0
Sphingomonas(100)	Otu098968	0	0	0	0.00012482836100362	0
Novosphingobium(100)	Otu099018	0	0	0	0.00012482836100362	0
Sphingobium(100)	Otu099069	0	0	0	0.00012482836100362	0
Novosphingobium(100)	Otu099072	0	0	0	0.00012482836100362	0
Sphingobium(100)	Otu099090	0	0	0	0.00012482836100362	0
Novosphingobium(100)	Otu099119	0.0000778513040093422	0	0	0	0
unclassified(100)	Otu099121	0.0000778513040093422	0	0	0	0
Sphingomonas(100)	Otu099122	0.0000778513040093422	0	0	0	0
Sphingopyxis(100)	Otu099134	0	0	0	0.00012482836100362	0
Blastomonas(100)	Otu099168	0.0000778513040093422	0	0	0	0
Novosphingobium(100)	Otu099175	0.0000778513040093422	0	0	0	0
unclassified(100)	Otu099190	0	0	0	0.00012482836100362	0
Sphingobium(100)	Otu099398	0	0	0	0.00012482836100362	0
Novosphingobium(100)	Otu100600	0	0.000124968757810547	0	0	0
Sphingomonas(100)	Otu100607	0.0000778513040093422	0	0	0	0
unclassified(100)	Otu100632	0.0000778513040093422	0	0	0	0
Sphingomonas(100)	Otu100638	0.0000778513040093422	0	0	0	0
Sphingomonas(100)	Otu100646	0.0000778513040093422	0	0	0	0
unclassified(100)	Otu100820	0.0000778513040093422	0	0	0	0
unclassified(100)	Otu101231	0.0000778513040093422	0	0	0	0
unclassified(100)	Otu101233	0.0000778513040093422	0	0	0	0
Novosphingobium(100)	Otu101235	0.0000778513040093422	0	0	0	0
Novosphingobium(100)	Otu101271	0.0000778513040093422	0	0	0	0
Novosphingobium(100)	Otu101289	0.0000778513040093422	0	0	0	0
Novosphingobium(100)	Otu101291	0.0000778513040093422	0	0	0	0
Sphingomonas(100)	Otu101305	0	0.000124968757810547	0	0	0

Novosphingobium(100)	Otu101368	0.0000778513040093422	0	0	0	0
unclassified(100)	Otu101387	0.0000778513040093422	0	0	0	0
unclassified(100)	Otu101400	0.0000778513040093422	0	0	0	0
Novosphingobium(100)	Otu101401	0.0000778513040093422	0	0	0	0
unclassified(100)	Otu101413	0.0000778513040093422	0	0	0	0
unclassified(100)	Otu101425	0.0000778513040093422	0	0	0	0
unclassified(100)	Otu101429	0	0.000124968757810547	0	0	0
unclassified(100)	Otu101434	0.0000778513040093422	0	0	0	0
unclassified(100)	Otu105370	0	0	0.000109075043630017	0	0
unclassified(100)	Otu105371	0	0	0	0.00099862688802896	0
unclassified(100)	Otu105372	0	0	0	0.00012482836100362	0
unclassified(100)	Otu105383	0	0	0	0	0.000110387459984546
unclassified(100)	Otu105420	0	0.000124968757810547	0	0	0
unclassified(100)	Otu105421	0	0	0.000109075043630017	0	0
unclassified(100)	Otu105428	0	0	0.000109075043630017	0	0
unclassified(100)	Otu105430	0	0	0.000109075043630017	0	0
unclassified(100)	Otu105665	0	0.000124968757810547	0	0	0
unclassified(100)	Otu105803	0	0.000124968757810547	0	0	0
unclassified(100)	Otu105805	0	0	0.000109075043630017	0	0
Legionella(100)	Otu106014	0	0	0	0	0.000110387459984546
Legionella(100)	Otu106020	0	0	0	0	0.000110387459984546
Legionella(100)	Otu106031	0	0	0	0	0.000110387459984546
Legionella(100)	Otu106040	0	0	0	0	0.000441549839938183
unclassified(100)	Otu106045	0	0.000124968757810547	0	0	0
unclassified(100)	Otu106051	0	0.000124968757810547	0	0	0
unclassified(100)	Otu106052	0	0	0	0.00012482836100362	0
unclassified(100)	Otu106053	0	0	0	0.00012482836100362	0
unclassified(100)	Otu106065	0	0	0	0	0.000110387459984546
unclassified(100)	Otu106075	0	0	0	0	0.000110387459984546
Afipia(100)	Otu106352	0	0.000124968757810547	0	0	0
unclassified(100)	Otu106354	0.0000778513040093422	0	0	0	0
unclassified(100)	Otu106410	0.0000778513040093422	0	0	0	0
Bradyrhizobium(100)	Otu106423	0	0	0.000109075043630017	0	0

Afipia(100)	Otu106462	0.0000778513040093422	0	0	0	0
Afipia(100)	Otu106486	0.0000778513040093422	0	0	0	0
unclassified(100)	Otu106487	0.0000778513040093422	0	0	0	0
unclassified(100)	Otu106499	0.0000778513040093422	0	0	0	0
unclassified(100)	Otu106509	0	0	0.000109075043630017	0	0
unclassified(100)	Otu106511	0	0	0.000109075043630017	0	0
unclassified(100)	Otu106512	0	0	0.000109075043630017	0	0
unclassified(100)	Otu106513	0	0	0.000109075043630017	0	0
unclassified(100)	Otu106515	0	0	0	0	0.000110387459984546
unclassified(100)	Otu106516	0	0	0.000109075043630017	0	0
unclassified(100)	Otu106524	0.0000778513040093422	0	0	0	0
Bradyrhizobium(100)	Otu106535	0.0000778513040093422	0	0	0	0
Afipia(100)	Otu106561	0.0000778513040093422	0	0	0	0
Afipia(100)	Otu106588	0.0000778513040093422	0	0	0	0
Spirosoma(100)	Otu106597	0.0000778513040093422	0	0	0	0
unclassified(100)	Otu106605	0.0000778513040093422	0	0	0	0
Spirosoma(100)	Otu106607	0.0000778513040093422	0	0	0	0
Spirosoma(100)	Otu106654	0.0000778513040093422	0	0	0	0
Spirosoma(100)	Otu106659	0.0000778513040093422	0	0	0	0
Spirosoma(100)	Otu106671	0.0000778513040093422	0	0	0	0
Arcicella(100)	Otu106672	0	0.000124968757810547	0	0	0
unclassified(100)	Otu106727	0	0	0	0	0.000110387459984546
Bradyrhizobium(100)	Otu106754	0	0	0	0	0.000110387459984546
unclassified(100)	Otu106814	0	0	0	0	0.000110387459984546
unclassified(100)	Otu106914	0.0000778513040093422	0	0	0	0
unclassified(100)	Otu107092	0.0000778513040093422	0	0	0	0
unclassified(100)	Otu107094	0.0000778513040093422	0	0	0	0
unclassified(100)	Otu107123	0	0.000124968757810547	0	0	0
unclassified(100)	Otu107128	0.0000778513040093422	0	0	0	0
unclassified(100)	Otu107131	0	0.000124968757810547	0	0	0
unclassified(100)	Otu107134	0	0.000124968757810547	0	0	0
unclassified(100)	Otu107135	0	0.000124968757810547	0	0	0
unclassified(100)	Otu107139	0.0000778513040093422	0	0	0	0

unclassified(100)	Otu107270	0.0000778513040093422	0	0	0	0
unclassified(100)	Otu107298	0.0000778513040093422	0	0	0	0
unclassified(100)	Otu107301	0.0000778513040093422	0	0	0	0
unclassified(100)	Otu107313	0.0000778513040093422	0	0	0	0
unclassified(100)	Otu107315	0.0000778513040093422	0	0	0	0
unclassified(100)	Otu107321	0.0000778513040093422	0	0	0	0
unclassified(100)	Otu107323	0.0000778513040093422	0	0	0	0
unclassified(100)	Otu107330	0.0000778513040093422	0	0	0	0
Neisseria(100)	Otu108333	0	0	0	0.00012482836100362	0
uncultured(100)	Otu108348	0	0	0	0	0.000110387459984546
proteobacterium_Ellin181(100)	Otu108399	0	0	0.000109075043630017	0	0
proteobacterium_Ellin181(100)	Otu108425	0	0	0.000109075043630017	0	0
Fusobacterium(100)	Otu108461	0	0	0	0.00012482836100362	0
uncultured_bacterium(100)	Otu108483	0	0	0.000109075043630017	0	0
unclassified(100)	Otu108488	0	0	0	0	0.000220774919969092
uncultured(100)	Otu108523	0	0	0	0	0.000110387459984546
Legionella(100)	Otu108626	0.0000778513040093422	0	0	0	0
Legionella(100)	Otu108630	0	0.000249937515621095	0	0	0
Legionella(100)	Otu108650	0	0	0.00043630017452007	0	0
unclassified(100)	Otu108682	0	0	0.000327225130890052	0	0
uncultured_bacterium(100)	Otu108747	0	0.00124968757810547	0	0	0
Leptotrichia(100)	Otu108811	0	0	0	0.00012482836100362	0
unclassified(100)	Otu108843	0	0	0	0.00012482836100362	0
unclassified(100)	Otu108863	0	0	0	0.00012482836100362	0
unclassified(100)	Otu108871	0	0.000124968757810547	0	0	0
uncultured_Bacteroidetes_bacterium(100)	Otu108924	0	0	0.000109075043630017	0	0
uncultured_Sphingomonadaceae_bacterium(100)	Otu109044	0	0	0	0.00024965672200724	0
unclassified(100)	Otu109092	0	0.000124968757810547	0	0	0
unclassified(100)	Otu109093	0	0	0.000109075043630017	0	0
unclassified(100)	Otu109094	0	0	0.000109075043630017	0	0
unclassified(100)	Otu109096	0	0	0	0	0.000110387459984546
unclassified(100)	Otu109173	0	0.000124968757810547	0	0	0
uncultured_bacterium(100)	Otu109180	0	0.000249937515621095	0	0	0

unclassified(100)	Otu109181	0	0.00162459385153712	0	0	0
Patulibacter(100)	Otu109201	0.0000778513040093422	0	0	0	0
unclassified(100)	Otu109229	0	0.000124968757810547	0	0	0
unclassified(100)	Otu109239	0	0	0.000109075043630017	0	0
unclassified(100)	Otu109283	0.0000778513040093422	0	0	0	0
unclassified(100)	Otu109567	0	0	0	0	0.000110387459984546
unclassified(100)	Otu109572	0	0	0	0	0.000110387459984546
Gluconobacter(100)	Otu109579	0	0	0	0	0.00331162379953637
unclassified(100)	Otu109605	0	0.000124968757810547	0	0	0
unclassified(100)	Otu109607	0	0.000124968757810547	0	0	0
uncultured(100)	Otu109614	0	0.000124968757810547	0	0	0
Craurococcus(100)	Otu109627	0	0	0	0.00037448508301086	0
unclassified(100)	Otu109628	0	0	0	0.00024965672200724	0
unclassified(100)	Otu109644	0	0	0.000109075043630017	0	0
unclassified(100)	Otu109647	0	0	0	0	0.000110387459984546
Gluconobacter(100)	Otu109672	0	0	0	0	0.000110387459984546
Tanticharoenia(100)	Otu109673	0	0	0	0	0.000110387459984546
Gluconobacter(100)	Otu109676	0	0	0	0	0.000110387459984546
unclassified(100)	Otu109733	0	0	0	0.00012482836100362	0
unclassified(100)	Otu109740	0	0	0	0.00012482836100362	0
Gluconobacter(100)	Otu109743	0	0	0	0	0.000110387459984546
Gluconobacter(100)	Otu109744	0	0	0	0	0.000110387459984546
Gluconobacter(100)	Otu109747	0	0	0	0	0.000110387459984546
unclassified(100)	Otu109752	0	0	0	0	0.000110387459984546
Gluconobacter(100)	Otu110284	0	0	0	0	0.000110387459984546
Gluconobacter(100)	Otu110289	0	0	0	0	0.000110387459984546
Gluconobacter(100)	Otu110304	0	0	0	0	0.000110387459984546
unclassified(100)	Otu110336	0	0	0	0	0.000110387459984546
Gluconobacter(100)	Otu110337	0	0	0	0	0.000110387459984546
Gluconobacter(100)	Otu110340	0	0	0	0	0.000110387459984546
unclassified(100)	Otu110344	0	0	0	0	0.000110387459984546
unclassified(100)	Otu110357	0	0	0	0	0.000110387459984546
Gluconobacter(100)	Otu110359	0	0	0	0	0.000110387459984546

unclassified(100)	Otu110367	0	0	0	0	0.000110387459984546
unclassified(100)	Otu110370	0	0	0	0	0.000110387459984546
Gluconobacter(100)	Otu110376	0	0	0	0	0.000110387459984546
unclassified(100)	Otu110379	0	0	0	0	0.000110387459984546
Gluconobacter(100)	Otu110383	0	0	0	0	0.000110387459984546
Gluconobacter(100)	Otu110384	0	0	0	0	0.000110387459984546
unclassified(100)	Otu110388	0	0	0	0	0.000110387459984546
Gluconobacter(100)	Otu110395	0	0	0	0	0.000110387459984546
Gluconobacter(100)	Otu110550	0	0	0	0	0.000110387459984546
Gluconobacter(100)	Otu110581	0	0	0	0	0.000110387459984546
unclassified(100)	Otu110582	0	0	0	0	0.000110387459984546
unclassified(100)	Otu110583	0	0	0	0	0.000110387459984546
unclassified(100)	Otu110591	0	0	0	0	0.000110387459984546
Gluconobacter(100)	Otu110594	0	0	0	0	0.000110387459984546
Gluconobacter(100)	Otu110595	0	0	0	0	0.000110387459984546
unclassified(100)	Otu110597	0	0	0	0	0.000110387459984546
Gluconobacter(100)	Otu110598	0	0	0	0	0.000110387459984546
unclassified(100)	Otu110619	0	0	0	0	0.000110387459984546
unclassified(100)	Otu110643	0	0	0	0	0.000110387459984546
unclassified(100)	Otu110647	0	0	0	0	0.000110387459984546
Gluconobacter(100)	Otu110649	0	0	0	0	0.000110387459984546
unclassified(100)	Otu110650	0	0	0	0	0.000110387459984546
unclassified(100)	Otu110654	0	0	0	0	0.000110387459984546
unclassified(100)	Otu110656	0	0	0	0	0.000110387459984546
Gluconobacter(100)	Otu110664	0	0	0	0	0.000110387459984546
unclassified(100)	Otu110739	0	0	0	0	0.000110387459984546
unclassified(100)	Otu110741	0	0	0	0	0.000110387459984546
Gluconobacter(100)	Otu110742	0	0	0	0	0.000110387459984546
unclassified(100)	Otu110743	0	0	0	0	0.000110387459984546
Gluconobacter(100)	Otu110744	0	0	0	0	0.000110387459984546
Gluconobacter(100)	Otu110785	0	0	0	0	0.000110387459984546
Gluconobacter(100)	Otu110787	0	0	0	0	0.000110387459984546
Gluconobacter(100)	Otu110790	0	0	0	0	0.000110387459984546

Gluconobacter(100)	Otu110812	0	0	0	0	0.000110387459984546
unclassified(100)	Otu110844	0	0	0	0	0.000110387459984546
unclassified(100)	Otu110849	0	0	0	0	0.000110387459984546
Gluconobacter(100)	Otu110850	0	0	0	0	0.000110387459984546
unclassified(100)	Otu110859	0	0	0	0	0.000110387459984546
Gluconobacter(100)	Otu110863	0	0	0	0	0.000110387459984546
Gluconobacter(100)	Otu110864	0	0	0	0	0.000110387459984546
Gluconobacter(100)	Otu110881	0	0	0	0	0.000110387459984546
Craurococcus(100)	Otu110925	0	0	0	0.00024965672200724	0
unclassified(100)	Otu111874	0	0.000124968757810547	0	0	0
unclassified(100)	Otu111875	0	0.000124968757810547	0	0	0
unclassified(100)	Otu111876	0	0.000124968757810547	0	0	0
unclassified(100)	Otu111886	0	0.000124968757810547	0	0	0
unclassified(100)	Otu111910	0	0.000124968757810547	0	0	0
unclassified(100)	Otu111912	0	0.000124968757810547	0	0	0
unclassified(100)	Otu118566	0	0	0.000109075043630017	0	0
unclassified(100)	Otu122060	0	0.000124968757810547	0	0	0
unclassified(100)	Otu122065	0	0.000124968757810547	0	0	0
unclassified(100)	Otu122067	0	0.000124968757810547	0	0	0
unclassified(100)	Otu122112	0	0.000124968757810547	0	0	0
unclassified(100)	Otu122118	0	0.000124968757810547	0	0	0
unclassified(100)	Otu122129	0	0.000124968757810547	0	0	0
unclassified(100)	Otu122160	0	0.000124968757810547	0	0	0
unclassified(100)	Otu122165	0	0.000124968757810547	0	0	0
unclassified(100)	Otu122292	0	0.000124968757810547	0	0	0
unclassified(100)	Otu122319	0	0.000124968757810547	0	0	0
unclassified(100)	Otu122320	0	0.000124968757810547	0	0	0
unclassified(100)	Otu122356	0	0.000124968757810547	0	0	0
unclassified(100)	Otu122361	0	0.000124968757810547	0	0	0
unclassified(100)	Otu122373	0	0.000124968757810547	0	0	0
unclassified(100)	Otu122378	0	0.000124968757810547	0	0	0
unclassified(100)	Otu122385	0.0000778513040093422	0	0	0	0
unclassified(100)	Otu122393	0	0.000124968757810547	0	0	0

unclassified(100)	Otu122404	0	0.000124968757810547	0	0	0
unclassified(100)	Otu122407	0	0.000124968757810547	0	0	0
unclassified(100)	Otu122414	0	0.000124968757810547	0	0	0
Acinetobacter(100)	Otu123034	0	0	0	0.00012482836100362	0
Enhydrobacter(100)	Otu123084	0	0	0	0	0.000110387459984546
Psychrobacter(100)	Otu123110	0	0	0	0.00012482836100362	0
Enhydrobacter(100)	Otu123258	0	0	0	0.00012482836100362	0
Acinetobacter(100)	Otu123267	0	0	0.000109075043630017	0	0
Acinetobacter(100)	Otu123388	0	0.000124968757810547	0	0	0
Acinetobacter(100)	Otu123434	0	0.000124968757810547	0	0	0
Acinetobacter(100)	Otu124132	0	0	0.000109075043630017	0	0
Acinetobacter(100)	Otu124176	0	0	0	0.00012482836100362	0
Alkanindiges(100)	Otu124228	0	0.000124968757810547	0	0	0
Alkanindiges(100)	Otu124517	0	0	0.000109075043630017	0	0
Acinetobacter(100)	Otu124528	0	0	0.000109075043630017	0	0
Acinetobacter(100)	Otu124529	0	0.000124968757810547	0	0	0
Acinetobacter(100)	Otu124537	0	0	0.000109075043630017	0	0
Acinetobacter(100)	Otu124553	0	0.000124968757810547	0	0	0
Acinetobacter(100)	Otu124584	0	0	0.000109075043630017	0	0
unclassified(100)	Otu124601	0	0	0	0.00012482836100362	0
Acinetobacter(100)	Otu124648	0	0.000124968757810547	0	0	0
Acinetobacter(100)	Otu124687	0	0.000124968757810547	0	0	0
Acinetobacter(100)	Otu124754	0	0	0	0.00012482836100362	0
Enhydrobacter(100)	Otu124828	0	0.000124968757810547	0	0	0
Acinetobacter(100)	Otu124904	0	0.000124968757810547	0	0	0
Acinetobacter(100)	Otu124951	0	0	0	0.00012482836100362	0
Enhydrobacter(100)	Otu124967	0	0	0	0.00012482836100362	0
unclassified(100)	Otu125021	0.0000778513040093422	0	0	0	0
unclassified(100)	Otu125023	0.0000778513040093422	0	0	0	0
unclassified(100)	Otu125028	0	0.000124968757810547	0	0	0
unclassified(100)	Otu125047	0.0000778513040093422	0	0	0	0
unclassified(100)	Otu125070	0	0.000124968757810547	0	0	0
Caulobacter(100)	Otu125079	0.0000778513040093422	0	0	0	0

unclassified(100)	Otu125098	0.0000778513040093422	0	0	0	0
Caulobacter(100)	Otu125109	0	0.000124968757810547	0	0	0
unclassified(100)	Otu125123	0	0.000124968757810547	0	0	0
unclassified(100)	Otu125127	0.0000778513040093422	0	0	0	0
Caulobacter(100)	Otu125128	0.0000778513040093422	0	0	0	0
Caulobacter(100)	Otu125136	0.0000778513040093422	0	0	0	0
unclassified(100)	Otu125140	0.0000778513040093422	0	0	0	0
unclassified(100)	Otu125173	0.0000778513040093422	0	0	0	0
Caulobacter(100)	Otu125181	0.0000778513040093422	0	0	0	0
Phenylobacterium(100)	Otu125182	0.0000778513040093422	0	0	0	0
unclassified(100)	Otu125187	0.0000778513040093422	0	0	0	0
unclassified(100)	Otu125202	0.0000778513040093422	0	0	0	0
unclassified(100)	Otu125208	0.0000778513040093422	0	0	0	0
Caulobacter(100)	Otu125228	0.0000778513040093422	0	0	0	0
unclassified(100)	Otu125230	0.0000778513040093422	0	0	0	0
Caulobacter(100)	Otu125288	0.0000778513040093422	0	0	0	0
unclassified(100)	Otu125361	0.0000778513040093422	0	0	0	0
unclassified(100)	Otu125367	0.0000778513040093422	0	0	0	0
unclassified(100)	Otu125380	0.0000778513040093422	0	0	0	0
Phenylobacterium(100)	Otu125390	0.0000778513040093422	0	0	0	0
unclassified(100)	Otu125406	0.0000778513040093422	0	0	0	0
unclassified(100)	Otu125409	0.0000778513040093422	0	0	0	0
Caulobacter(100)	Otu125411	0.0000778513040093422	0	0	0	0
unclassified(100)	Otu125417	0.0000778513040093422	0	0	0	0
unclassified(100)	Otu125434	0.0000778513040093422	0	0	0	0
unclassified(100)	Otu125438	0.0000778513040093422	0	0	0	0
Caulobacter(100)	Otu125443	0.0000778513040093422	0	0	0	0
unclassified(100)	Otu125480	0	0	0.000109075043630017	0	0
Caulobacter(100)	Otu125542	0.0000778513040093422	0	0	0	0
unclassified(100)	Otu125561	0.0000778513040093422	0	0	0	0
Caulobacter(100)	Otu125563	0.0000778513040093422	0	0	0	0
Caulobacter(100)	Otu125567	0	0.000124968757810547	0	0	0
unclassified(100)	Otu125592	0	0	0.000109075043630017	0	0

Brevundimonas(100)	Otu125594	0	0	0.000109075043630017	0	0
unclassified(100)	Otu125649	0	0	0.000109075043630017	0	0
Caulobacter(100)	Otu125728	0.0000778513040093422	0	0	0	0
unclassified(100)	Otu125736	0.0000778513040093422	0	0	0	0
Caulobacter(100)	Otu125742	0.0000778513040093422	0	0	0	0
unclassified(100)	Otu125753	0	0.000124968757810547	0	0	0
unclassified(100)	Otu125755	0.0000778513040093422	0	0	0	0
unclassified(100)	Otu125764	0.0000778513040093422	0	0	0	0
Caulobacter(100)	Otu125770	0.0000778513040093422	0	0	0	0
unclassified(100)	Otu125782	0.0000778513040093422	0	0	0	0
unclassified(100)	Otu125784	0.0000778513040093422	0	0	0	0
unclassified(100)	Otu125804	0.0000778513040093422	0	0	0	0
Caulobacter(100)	Otu125807	0.0000778513040093422	0	0	0	0
Brevundimonas(100)	Otu125816	0.0000778513040093422	0	0	0	0
Caulobacter(100)	Otu125817	0.0000778513040093422	0	0	0	0
Brevundimonas(100)	Otu125820	0.0000778513040093422	0	0	0	0
unclassified(100)	Otu125822	0.0000778513040093422	0	0	0	0
Caulobacter(100)	Otu125824	0	0.000124968757810547	0	0	0
unclassified(100)	Otu125840	0.0000778513040093422	0	0	0	0
Caulobacter(100)	Otu125843	0.0000778513040093422	0	0	0	0
unclassified(100)	Otu125849	0.0000778513040093422	0	0	0	0
unclassified(100)	Otu125851	0.0000778513040093422	0	0	0	0
unclassified(100)	Otu125856	0.0000778513040093422	0	0	0	0
Caulobacter(100)	Otu125876	0.0000778513040093422	0	0	0	0
Caulobacter(100)	Otu125882	0.0000778513040093422	0	0	0	0
unclassified(100)	Otu125885	0.0000778513040093422	0	0	0	0
Caulobacter(100)	Otu125886	0	0	0	0	0.000110387459984546
Caulobacter(100)	Otu125888	0.0000778513040093422	0	0	0	0
unclassified(100)	Otu125894	0.0000778513040093422	0	0	0	0
unclassified(100)	Otu125937	0.0000778513040093422	0	0	0	0
Caulobacter(100)	Otu125943	0.0000778513040093422	0	0	0	0
Caulobacter(100)	Otu125963	0.0000778513040093422	0	0	0	0
unclassified(100)	Otu125968	0.0000778513040093422	0	0	0	0

unclassified(100)	Otu125972	0.0000778513040093422	0	0	0	0
unclassified(100)	Otu125978	0.0000778513040093422	0	0	0	0
unclassified(100)	Otu125980	0.0000778513040093422	0	0	0	0
unclassified(100)	Otu125982	0.0000778513040093422	0	0	0	0
unclassified(100)	Otu125994	0.0000778513040093422	0	0	0	0
unclassified(100)	Otu125996	0.0000778513040093422	0	0	0	0
unclassified(100)	Otu125997	0.0000778513040093422	0	0	0	0
unclassified(100)	Otu125999	0.0000778513040093422	0	0	0	0
unclassified(100)	Otu126003	0.0000778513040093422	0	0	0	0
unclassified(100)	Otu126004	0.0000778513040093422	0	0	0	0
unclassified(100)	Otu126006	0.0000778513040093422	0	0	0	0
unclassified(100)	Otu126007	0.0000778513040093422	0	0	0	0
unclassified(100)	Otu126011	0.0000778513040093422	0	0	0	0
unclassified(100)	Otu126020	0.0000778513040093422	0	0	0	0
unclassified(100)	Otu126021	0.0000778513040093422	0	0	0	0
unclassified(100)	Otu126026	0.0000778513040093422	0	0	0	0
unclassified(100)	Otu126027	0.0000778513040093422	0	0	0	0
unclassified(100)	Otu126030	0.0000778513040093422	0	0	0	0
unclassified(100)	Otu126034	0.0000778513040093422	0	0	0	0
unclassified(100)	Otu126038	0.0000778513040093422	0	0	0	0
unclassified(100)	Otu126066	0.0000778513040093422	0	0	0	0
unclassified(100)	Otu126069	0.0000778513040093422	0	0	0	0
unclassified(100)	Otu126075	0.0000778513040093422	0	0	0	0
unclassified(100)	Otu126080	0.0000778513040093422	0	0	0	0
unclassified(100)	Otu126128	0.0000778513040093422	0	0	0	0
unclassified(100)	Otu126156	0.0000778513040093422	0	0	0	0
unclassified(100)	Otu126169	0.0000778513040093422	0	0	0	0
unclassified(100)	Otu126172	0.0000778513040093422	0	0	0	0
unclassified(100)	Otu126176	0.0000778513040093422	0	0	0	0
unclassified(100)	Otu126180	0.0000778513040093422	0	0	0	0
unclassified(100)	Otu126183	0.0000778513040093422	0	0	0	0
unclassified(100)	Otu126184	0.0000778513040093422	0	0	0	0
unclassified(100)	Otu126446	0.0000778513040093422	0	0	0	0

unclassified(100)	Otu126523	0	0	0	0	0.000110387459984546
unclassified(100)	Otu126524	0	0	0	0	0.000110387459984546
unclassified(100)	Otu126529	0	0	0	0	0.000110387459984546
unclassified(100)	Otu126532	0	0	0	0	0.000110387459984546
unclassified(100)	Otu126546	0	0	0	0	0.000110387459984546
unclassified(100)	Otu126715	0	0	0	0	0.000110387459984546
unclassified(100)	Otu126717	0	0	0	0	0.000110387459984546
unclassified(100)	Otu126719	0	0	0	0	0.000110387459984546
unclassified(100)	Otu126793	0.0000778513040093422	0	0	0	0
unclassified(100)	Otu126851	0	0	0	0	0.000110387459984546
Rhizobium(100)	Otu126973	0	0	0	0	0.000110387459984546
Rhizobium(100)	Otu126978	0.0000778513040093422	0	0	0	0
Rhizobium(100)	Otu126980	0.0000778513040093422	0	0	0	0
Rhizobium(100)	Otu126996	0	0	0	0	0.000110387459984546
Rhizobium(100)	Otu127001	0	0	0	0	0.000110387459984546
Rhizobium(100)	Otu127046	0.0000778513040093422	0	0	0	0
Rhizobium(100)	Otu127267	0	0	0	0.00012482836100362	0
Rhizobium(100)	Otu127277	0	0	0.000109075043630017	0	0
Rhizobium(100)	Otu127280	0	0	0.000109075043630017	0	0
Rhizobium(100)	Otu127288	0	0	0	0.00012482836100362	0
Rhizobium(100)	Otu127340	0	0	0	0	0.000110387459984546
Rhizobium(100)	Otu127342	0	0	0	0.00012482836100362	0
Rhizobium(100)	Otu127434	0	0	0	0	0.000110387459984546
Rhizobium(100)	Otu127438	0	0	0	0	0.000110387459984546
unclassified(100)	Otu127453	0	0	0	0	0.000220774919969092
Flectobacillus(100)	Otu127459	0	0	0.000109075043630017	0	0
Hymenobacter(100)	Otu127463	0	0.000124968757810547	0	0	0
Hymenobacter(100)	Otu127465	0	0	0	0	0.000110387459984546
Hymenobacter(100)	Otu127467	0	0	0	0	0.000110387459984546
Hymenobacter(100)	Otu127469	0	0.000124968757810547	0	0	0
Hymenobacter(100)	Otu127471	0	0	0	0	0.000110387459984546
Hymenobacter(100)	Otu127517	0	0	0	0.00012482836100362	0
Flexibacter(100)	Otu127525	0	0	0	0.00012482836100362	0

Hymenobacter(100)	Otu127527	0	0	0	0.00012482836100362	0
Hymenobacter(100)	Otu127529	0	0	0	0	0.000110387459984546
Spirosoma(100)	Otu127535	0.0000778513040093422	0	0	0	0
unclassified(100)	Otu127543	0.0000778513040093422	0	0	0	0
Spirosoma(100)	Otu127545	0.0000778513040093422	0	0	0	0
Spirosoma(100)	Otu127553	0.0000778513040093422	0	0	0	0
Spirosoma(100)	Otu127554	0.0000778513040093422	0	0	0	0
Hymenobacter(100)	Otu127558	0	0.000124968757810547	0	0	0
Hymenobacter(100)	Otu127560	0	0.000124968757810547	0	0	0
Hymenobacter(100)	Otu127564	0	0.000124968757810547	0	0	0
unclassified(100)	Otu127636	0	0	0	0	0.000220774919969092
Hymenobacter(100)	Otu127637	0	0	0	0	0.000110387459984546
Hymenobacter(100)	Otu127644	0	0.000124968757810547	0	0	0
Hymenobacter(100)	Otu127842	0	0	0	0.00012482836100362	0
Hymenobacter(100)	Otu127843	0	0	0.000109075043630017	0	0
Caulobacter(100)	Otu127945	0	0	0	0.00012482836100362	0
unclassified(100)	Otu128032	0.0000778513040093422	0	0	0	0
Caulobacter(100)	Otu128034	0	0	0.000109075043630017	0	0
Brevundimonas(100)	Otu128038	0	0	0	0.00012482836100362	0
unclassified(100)	Otu128201	0	0	0.000109075043630017	0	0
Caulobacter(100)	Otu128211	0	0	0.000109075043630017	0	0
Phenylobacterium(100)	Otu128245	0	0	0.000109075043630017	0	0
Phenylobacterium(100)	Otu128290	0	0	0.000109075043630017	0	0
Brevundimonas(100)	Otu128353	0	0	0	0	0.000110387459984546
Brevundimonas(100)	Otu128380	0	0	0	0.00012482836100362	0
unclassified(100)	Otu128410	0	0	0	0	0.000110387459984546
unclassified(100)	Otu128411	0	0	0	0	0.000110387459984546
Rhizobium(100)	Otu128551	0	0.000124968757810547	0	0	0
Rhizobium(100)	Otu128657	0.0000778513040093422	0	0	0	0
Rhizobium(100)	Otu128659	0.0000778513040093422	0	0	0	0
unclassified(100)	Otu128898	0	0	0	0.00012482836100362	0
unclassified(100)	Otu128918	0.0000778513040093422	0	0	0	0
unclassified(100)	Otu129039	0.0000778513040093422	0	0	0	0

unclassified(100)	Otu129041	0.0000778513040093422	0	0	0	0
unclassified(100)	Otu129050	0.0000778513040093422	0	0	0	0
unclassified(100)	Otu129051	0.0000778513040093422	0	0	0	0
unclassified(100)	Otu129061	0.0000778513040093422	0	0	0	0
unclassified(100)	Otu129063	0.0000778513040093422	0	0	0	0
unclassified(100)	Otu129064	0.0000778513040093422	0	0	0	0
unclassified(100)	Otu129065	0.0000778513040093422	0	0	0	0
unclassified(100)	Otu129066	0.0000778513040093422	0	0	0	0
unclassified(100)	Otu129079	0.0000778513040093422	0	0	0	0
unclassified(100)	Otu129081	0.0000778513040093422	0	0	0	0
unclassified(100)	Otu129083	0.0000778513040093422	0	0	0	0
unclassified(100)	Otu129091	0.0000778513040093422	0	0	0	0
unclassified(100)	Otu129093	0.0000778513040093422	0	0	0	0
unclassified(100)	Otu129132	0.0000778513040093422	0	0	0	0
unclassified(100)	Otu129676	0.0000778513040093422	0	0	0	0
unclassified(100)	Otu129677	0.0000778513040093422	0	0	0	0
unclassified(100)	Otu129713	0.0000778513040093422	0	0	0	0
unclassified(100)	Otu129716	0.0000778513040093422	0	0	0	0
unclassified(100)	Otu129717	0.0000778513040093422	0	0	0	0
unclassified(100)	Otu129723	0.0000778513040093422	0	0	0	0
unclassified(100)	Otu129726	0.0000778513040093422	0	0	0	0
unclassified(100)	Otu129728	0.0000778513040093422	0	0	0	0
unclassified(100)	Otu129733	0.0000778513040093422	0	0	0	0
unclassified(100)	Otu129735	0.0000778513040093422	0	0	0	0
unclassified(100)	Otu129744	0.0000778513040093422	0	0	0	0
unclassified(100)	Otu129752	0.0000778513040093422	0	0	0	0
unclassified(100)	Otu129756	0.0000778513040093422	0	0	0	0
unclassified(100)	Otu129767	0.0000778513040093422	0	0	0	0
unclassified(100)	Otu130919	0	0	0	0	0.000110387459984546
unclassified(100)	Otu131121	0.0000778513040093422	0	0	0	0
unclassified(100)	Otu131125	0.0000778513040093422	0	0	0	0
unclassified(100)	Otu131134	0	0.000124968757810547	0	0	0
unclassified(100)	Otu131135	0.0000778513040093422	0	0	0	0

unclassified(100)	Otu131139	0.0000778513040093422	0	0	0	0
unclassified(100)	Otu131146	0	0.000124968757810547	0	0	0
unclassified(100)	Otu131164	0	0.000124968757810547	0	0	0
unclassified(100)	Otu131165	0	0.000124968757810547	0	0	0
unclassified(100)	Otu131171	0	0.000124968757810547	0	0	0
unclassified(100)	Otu131174	0	0.000124968757810547	0	0	0
unclassified(100)	Otu131178	0	0.000124968757810547	0	0	0
unclassified(100)	Otu131181	0	0.000124968757810547	0	0	0
unclassified(100)	Otu131183	0	0.000124968757810547	0	0	0
unclassified(100)	Otu131194	0	0.000124968757810547	0	0	0
unclassified(100)	Otu131200	0	0.000124968757810547	0	0	0
unclassified(100)	Otu131209	0	0	0.000109075043630017	0	0
unclassified(100)	Otu131210	0	0.000124968757810547	0	0	0
unclassified(100)	Otu131211	0	0.000124968757810547	0	0	0
unclassified(100)	Otu131213	0	0.000124968757810547	0	0	0
unclassified(100)	Otu131215	0	0.000124968757810547	0	0	0
unclassified(100)	Otu131223	0	0.000124968757810547	0	0	0
unclassified(100)	Otu131232	0	0.000124968757810547	0	0	0
unclassified(100)	Otu131233	0	0.000124968757810547	0	0	0
unclassified(100)	Otu131237	0	0.000124968757810547	0	0	0
unclassified(100)	Otu131239	0	0.000124968757810547	0	0	0
unclassified(100)	Otu131241	0	0.000124968757810547	0	0	0
unclassified(100)	Otu131271	0.0000778513040093422	0	0	0	0
unclassified(100)	Otu131290	0.0000778513040093422	0	0	0	0
unclassified(100)	Otu131305	0	0	0.000109075043630017	0	0
unclassified(100)	Otu131312	0.0000778513040093422	0	0	0	0
unclassified(100)	Otu131315	0.0000778513040093422	0	0	0	0
unclassified(100)	Otu131316	0	0.000999750062484379	0	0	0
unclassified(100)	Otu131320	0	0	0.000109075043630017	0	0
unclassified(100)	Otu131324	0.0000778513040093422	0	0	0	0
unclassified(100)	Otu131325	0.0000778513040093422	0	0	0	0
unclassified(100)	Otu131327	0.0000778513040093422	0	0	0	0
unclassified(100)	Otu131331	0	0.000124968757810547	0	0	0

unclassified(100)	Otu131333	0	0.000124968757810547	0	0	0
unclassified(100)	Otu131336	0.0000778513040093422	0	0	0	0
unclassified(100)	Otu131339	0.0000778513040093422	0	0	0	0
unclassified(100)	Otu131341	0.0000778513040093422	0	0	0	0
unclassified(100)	Otu131343	0	0.000124968757810547	0	0	0
unclassified(100)	Otu131353	0.0000778513040093422	0	0	0	0
unclassified(100)	Otu131355	0.0000778513040093422	0	0	0	0
unclassified(100)	Otu131369	0	0	0	0	0.000110387459984546
unclassified(100)	Otu131370	0	0	0	0	0.000110387459984546
unclassified(100)	Otu131372	0	0	0	0	0.000110387459984546
unclassified(100)	Otu131375	0	0	0	0	0.000110387459984546
unclassified(100)	Otu131380	0	0.000124968757810547	0	0	0
unclassified(100)	Otu131383	0	0.000124968757810547	0	0	0
unclassified(100)	Otu131385	0	0.000124968757810547	0	0	0
unclassified(100)	Otu131387	0	0.000124968757810547	0	0	0
unclassified(100)	Otu131396	0	0	0	0	0.000110387459984546
unclassified(100)	Otu131398	0	0	0.000109075043630017	0	0
unclassified(100)	Otu131403	0	0	0.000109075043630017	0	0
unclassified(100)	Otu131404	0	0	0	0	0.000110387459984546
unclassified(100)	Otu131405	0	0	0	0	0.000110387459984546
unclassified(100)	Otu131409	0	0	0	0	0.000110387459984546
unclassified(100)	Otu131411	0	0	0	0	0.000110387459984546
unclassified(100)	Otu131412	0	0	0	0	0.000110387459984546
unclassified(100)	Otu131413	0	0	0	0	0.000110387459984546
unclassified(100)	Otu131414	0	0	0	0	0.000110387459984546
unclassified(100)	Otu131418	0	0	0	0	0.000110387459984546
unclassified(100)	Otu131421	0	0	0	0	0.000110387459984546
unclassified(100)	Otu131423	0	0	0	0	0.000110387459984546
unclassified(100)	Otu131424	0	0	0	0	0.000110387459984546
unclassified(100)	Otu131426	0	0	0	0	0.000110387459984546
unclassified(100)	Otu131428	0	0	0	0	0.000110387459984546
unclassified(100)	Otu131429	0	0	0	0	0.000110387459984546
unclassified(100)	Otu131430	0	0	0	0	0.000110387459984546

unclassified(100)	Otu131431	0	0	0	0	0.000110387459984546
unclassified(100)	Otu131435	0	0	0	0	0.000110387459984546
unclassified(100)	Otu131436	0	0	0	0	0.000110387459984546
unclassified(100)	Otu131439	0	0	0	0	0.000110387459984546
unclassified(100)	Otu131441	0	0	0	0	0.000110387459984546
unclassified(100)	Otu131443	0	0	0	0	0.000110387459984546
unclassified(100)	Otu131446	0	0	0	0	0.000110387459984546
unclassified(100)	Otu131451	0	0	0	0	0.000110387459984546
unclassified(100)	Otu131452	0	0	0	0	0.000110387459984546
unclassified(100)	Otu131456	0	0	0	0	0.000110387459984546
unclassified(100)	Otu131463	0	0	0	0	0.000110387459984546
unclassified(100)	Otu131465	0	0	0.000109075043630017	0	0
unclassified(100)	Otu131467	0	0	0	0	0.000110387459984546
unclassified(100)	Otu131473	0	0	0	0	0.000110387459984546
unclassified(100)	Otu131478	0	0	0	0	0.000110387459984546
unclassified(100)	Otu131483	0	0	0	0	0.000110387459984546
unclassified(100)	Otu131485	0	0	0	0	0.000110387459984546
unclassified(100)	Otu131489	0	0	0	0	0.000110387459984546
unclassified(100)	Otu131490	0	0	0	0	0.000110387459984546
unclassified(100)	Otu131497	0	0	0	0	0.000110387459984546
unclassified(100)	Otu131507	0	0	0	0	0.000110387459984546
unclassified(100)	Otu131511	0	0	0	0	0.000110387459984546
unclassified(100)	Otu131512	0	0	0	0	0.000110387459984546
unclassified(100)	Otu131513	0	0	0	0	0.000110387459984546
unclassified(100)	Otu131518	0	0	0	0	0.000110387459984546
unclassified(100)	Otu131525	0	0	0	0.00012482836100362	0
unclassified(100)	Otu131529	0	0	0	0.00012482836100362	0
unclassified(100)	Otu131533	0	0	0	0.00012482836100362	0
unclassified(100)	Otu131535	0	0	0	0	0.000110387459984546
unclassified(100)	Otu131537	0	0	0	0.00012482836100362	0
unclassified(100)	Otu131548	0	0	0	0.00012482836100362	0
unclassified(100)	Otu131550	0	0	0.000109075043630017	0	0
unclassified(100)	Otu131555	0	0	0.000109075043630017	0	0

unclassified(100)	Otu131557	0	0	0.000109075043630017	0	0
unclassified(100)	Otu131558	0	0	0.000109075043630017	0	0
unclassified(100)	Otu131561	0	0	0.000109075043630017	0	0
unclassified(100)	Otu131566	0	0	0	0.00012482836100362	0
unclassified(100)	Otu131568	0	0	0.000109075043630017	0	0
unclassified(100)	Otu131571	0	0	0.000109075043630017	0	0
unclassified(100)	Otu131572	0	0	0	0	0.000110387459984546
unclassified(100)	Otu131573	0	0	0.000109075043630017	0	0
unclassified(100)	Otu131579	0	0	0	0.00012482836100362	0
unclassified(100)	Otu131581	0	0	0.000109075043630017	0	0
unclassified(100)	Otu131585	0	0	0	0.00012482836100362	0
unclassified(100)	Otu131586	0	0	0	0.00012482836100362	0
unclassified(100)	Otu131587	0	0	0	0.00012482836100362	0
unclassified(100)	Otu131593	0	0	0	0.00012482836100362	0
unclassified(100)	Otu131594	0	0	0	0.00012482836100362	0
unclassified(100)	Otu131597	0	0	0.000109075043630017	0	0
unclassified(100)	Otu131604	0	0	0	0.00012482836100362	0
unclassified(100)	Otu131606	0	0	0	0	0.000110387459984546
unclassified(100)	Otu131607	0	0	0	0	0.000110387459984546
unclassified(100)	Otu131608	0	0	0	0	0.000110387459984546
unclassified(100)	Otu131609	0	0	0	0	0.000110387459984546
unclassified(100)	Otu131610	0	0	0	0	0.000110387459984546
unclassified(100)	Otu131616	0	0	0	0.00012482836100362	0
unclassified(100)	Otu131624	0	0	0	0.00012482836100362	0
unclassified(100)	Otu131670	0	0	0	0.00012482836100362	0
unclassified(100)	Otu131704	0	0	0	0.00012482836100362	0
unclassified(100)	Otu131708	0	0	0	0.00012482836100362	0
unclassified(100)	Otu131714	0	0	0	0.00012482836100362	0
unclassified(100)	Otu131717	0	0	0	0.00012482836100362	0
unclassified(100)	Otu131718	0	0	0	0.00012482836100362	0
unclassified(100)	Otu131726	0	0	0	0.00012482836100362	0
unclassified(100)	Otu131732	0	0	0	0.00012482836100362	0
unclassified(100)	Otu131772	0	0	0	0	0.000110387459984546

unclassified(100)	Otu132470	0	0	0.000109075043630017	0	0
unclassified(100)	Otu132471	0	0	0.000109075043630017	0	0
unclassified(100)	Otu132476	0	0	0.000109075043630017	0	0
unclassified(100)	Otu132477	0	0	0.000109075043630017	0	0
unclassified(100)	Otu132480	0	0	0.000109075043630017	0	0
unclassified(100)	Otu132482	0	0	0.000109075043630017	0	0
unclassified(100)	Otu132483	0	0	0.000109075043630017	0	0
unclassified(100)	Otu132484	0	0	0.000109075043630017	0	0
unclassified(100)	Otu132488	0	0	0.000109075043630017	0	0
unclassified(100)	Otu132491	0	0	0.000109075043630017	0	0
unclassified(100)	Otu132494	0	0	0.000109075043630017	0	0
unclassified(100)	Otu132497	0	0	0.000109075043630017	0	0
unclassified(100)	Otu132498	0	0	0.000109075043630017	0	0
unclassified(100)	Otu132499	0	0	0.000109075043630017	0	0
unclassified(100)	Otu132502	0	0.000124968757810547	0	0	0
unclassified(100)	Otu132505	0	0.000124968757810547	0	0	0
unclassified(100)	Otu132508	0	0.000124968757810547	0	0	0
unclassified(100)	Otu132510	0	0.000249937515621095	0	0	0
unclassified(100)	Otu132512	0	0	0.000109075043630017	0	0
unclassified(100)	Otu132514	0	0	0.000109075043630017	0	0
unclassified(100)	Otu132515	0	0	0.000109075043630017	0	0
unclassified(100)	Otu132517	0	0	0.000109075043630017	0	0
unclassified(100)	Otu132518	0	0	0.000109075043630017	0	0
unclassified(100)	Otu132523	0	0	0.000109075043630017	0	0
unclassified(100)	Otu132525	0	0	0.000109075043630017	0	0
unclassified(100)	Otu132529	0	0	0.000109075043630017	0	0
unclassified(100)	Otu132566	0	0	0.000109075043630017	0	0
unclassified(100)	Otu132570	0	0	0.000109075043630017	0	0
unclassified(100)	Otu132571	0	0	0.000109075043630017	0	0
unclassified(100)	Otu132573	0	0	0.000109075043630017	0	0
unclassified(100)	Otu166928	0	0.000124968757810547	0	0	0
unclassified(100)	Otu167036	0	0.000124968757810547	0	0	0
unclassified(100)	Otu167722	0	0	0.000109075043630017	0	0

unclassified(100)	Otu168364	0	0	0	0.00012482836100362	0
unclassified(100)	Otu168446	0	0	0	0.00012482836100362	0
Stenotrophomonas(100)	Otu168449	0	0	0	0.00012482836100362	0
Stenotrophomonas(100)	Otu168457	0	0	0	0.00012482836100362	0
unclassified(100)	Otu168460	0	0	0	0.00012482836100362	0
unclassified(100)	Otu168473	0	0	0	0.00012482836100362	0
unclassified(100)	Otu168476	0	0	0	0.00012482836100362	0
unclassified(100)	Otu170944	0	0	0	0	0.000110387459984546
Flavobacterium(100)	Otu170950	0	0	0	0	0.000110387459984546
unclassified(100)	Otu170951	0	0	0	0	0.000110387459984546
unclassified(100)	Otu170955	0	0	0	0	0.000110387459984546
Chryseobacterium(100)	Otu170957	0	0	0	0	0.000110387459984546
Flavobacterium(100)	Otu170958	0	0	0	0	0.000110387459984546
Chryseobacterium(100)	Otu170960	0	0	0	0	0.000110387459984546
Chryseobacterium(100)	Otu170961	0	0	0	0	0.000110387459984546
unclassified(100)	Otu171097	0	0	0	0	0.000110387459984546
unclassified(100)	Otu172223	0	0	0	0.00012482836100362	0
unclassified(100)	Otu172232	0	0	0	0.00012482836100362	0
unclassified(100)	Otu172238	0	0	0	0.00012482836100362	0
unclassified(100)	Otu172366	0	0	0	0	0.000110387459984546
unclassified(100)	Otu172368	0	0	0	0.00012482836100362	0
unclassified(100)	Otu172374	0	0.000124968757810547	0	0	0
unclassified(100)	Otu172376	0	0.000124968757810547	0	0	0
unclassified(100)	Otu172387	0	0	0	0	0.000110387459984546
unclassified(100)	Otu172392	0	0	0.000109075043630017	0	0
unclassified(100)	Otu172398	0	0	0	0.00012482836100362	0
unclassified(100)	Otu172399	0	0	0	0.00012482836100362	0
unclassified(100)	Otu172401	0	0	0	0.00012482836100362	0
unclassified(100)	Otu172434	0	0.000124968757810547	0	0	0
unclassified(100)	Otu173722	0.0000778513040093422	0	0	0	0
unclassified(100)	Otu173725	0.0000778513040093422	0	0	0	0
unclassified(100)	Otu173727	0.0000778513040093422	0	0	0	0
unclassified(100)	Otu173779	0	0	0.000109075043630017	0	0

unclassified(100)	Otu173781	0	0	0.000109075043630017	0	0
unclassified(100)	Otu173782	0	0	0.000109075043630017	0	0
unclassified(100)	Otu173783	0	0	0.000109075043630017	0	0
unclassified(100)	Otu173787	0	0	0.000109075043630017	0	0
unclassified(100)	Otu173788	0	0	0.000109075043630017	0	0
unclassified(100)	Otu173789	0	0	0.000109075043630017	0	0
unclassified(100)	Otu173796	0.0000778513040093422	0	0	0	0
unclassified(100)	Otu173800	0.0000778513040093422	0	0	0	0
unclassified(100)	Otu173806	0	0.000124968757810547	0	0	0
unclassified(100)	Otu173808	0	0.000124968757810547	0	0	0
unclassified(100)	Otu173811	0.0000778513040093422	0	0	0	0
unclassified(100)	Otu173828	0.0000778513040093422	0	0	0	0
unclassified(100)	Otu173829	0.0000778513040093422	0	0	0	0
unclassified(100)	Otu173840	0.0000778513040093422	0	0	0	0
unclassified(100)	Otu173894	0.0000778513040093422	0	0	0	0
unclassified(100)	Otu173896	0.0000778513040093422	0	0	0	0
unclassified(100)	Otu173899	0.0000778513040093422	0	0	0	0
unclassified(100)	Otu174098	0	0	0	0	0.000110387459984546
unclassified(100)	Otu174099	0	0	0	0	0.000110387459984546
unclassified(100)	Otu174100	0	0	0	0	0.000110387459984546
unclassified(100)	Otu174104	0	0	0.000109075043630017	0	0
unclassified(100)	Otu174106	0	0	0.000109075043630017	0	0
unclassified(100)	Otu174108	0	0	0	0	0.000110387459984546
unclassified(100)	Otu174109	0	0	0	0.00012482836100362	0
unclassified(100)	Otu174110	0	0	0.000109075043630017	0	0
unclassified(100)	Otu174111	0	0	0	0	0.000110387459984546
unclassified(100)	Otu174114	0	0	0	0.00012482836100362	0
unclassified(100)	Otu174119	0	0	0	0	0.000110387459984546
unclassified(100)	Otu174142	0	0	0.000109075043630017	0	0
unclassified(100)	Otu174143	0	0.000124968757810547	0	0	0
unclassified(100)	Otu174147	0	0	0.000109075043630017	0	0
unclassified(100)	Otu174154	0	0	0.000109075043630017	0	0
unclassified(100)	Otu174155	0	0	0.000109075043630017	0	0

unclassified(100)	Otu174157	0	0	0.000109075043630017	0	0
unclassified(100)	Otu174161	0	0	0	0	0.000110387459984546
unclassified(100)	Otu174172	0	0	0	0	0.000110387459984546
unclassified(100)	Otu174173	0	0	0	0	0.000110387459984546
unclassified(100)	Otu174174	0	0	0	0	0.000110387459984546
unclassified(100)	Otu174194	0	0	0	0	0.000110387459984546
unclassified(100)	Otu174196	0	0.000124968757810547	0	0	0
unclassified(100)	Otu174201	0	0.000124968757810547	0	0	0
unclassified(100)	Otu174206	0	0	0	0	0.000110387459984546
unclassified(100)	Otu174209	0	0	0.000109075043630017	0	0
unclassified(100)	Otu174211	0	0	0	0	0.000110387459984546
unclassified(100)	Otu174218	0	0	0	0	0.000110387459984546
unclassified(100)	Otu174259	0.0000778513040093422	0	0	0	0
unclassified(100)	Otu174261	0.0000778513040093422	0	0	0	0
unclassified(100)	Otu174266	0.0000778513040093422	0	0	0	0
unclassified(100)	Otu174362	0	0	0.000109075043630017	0	0
unclassified(100)	Otu174368	0	0	0.000109075043630017	0	0
unclassified(100)	Otu174397	0	0	0	0	0.000110387459984546
unclassified(100)	Otu174398	0	0	0	0	0.000110387459984546
unclassified(100)	Otu174399	0	0	0	0	0.000110387459984546
unclassified(100)	Otu174742	0	0	0	0.00012482836100362	0
unclassified(100)	Otu174747	0	0	0	0.00012482836100362	0
unclassified(100)	Otu174748	0	0	0	0.00012482836100362	0
unclassified(100)	Otu174751	0	0	0	0.00012482836100362	0
unclassified(100)	Otu174762	0	0	0	0	0.000110387459984546
Beta_vulgaris_subsp._vulgaris(100)	Otu174763	0	0	0	0	0.000110387459984546
unclassified(100)	Otu175839	0	0	0.000109075043630017	0	0
Candidatus_Rhabdochlamydia(100)	Otu175950	0	0	0	0	0.000220774919969092
unclassified(100)	Otu176005	0	0	0	0	0.000331162379953637
unclassified(100)	Otu176146	0	0	0	0.00012482836100362	0
unclassified(100)	Otu176294	0	0.000124968757810547	0	0	0
unclassified(100)	Otu176370	0	0	0	0	0.000110387459984546
Caldanaerobius(100)	Otu176440	0	0	0	0.00012482836100362	0

Truepera(100)	Otu176441	0	0	0	0	0.000331162379953637
unclassified(100)	Otu176527	0	0	0	0.00012482836100362	0
Incertae_Sedis(100)	Otu176546	0	0	0	0	0.000110387459984546
Incertae_Sedis(100)	Otu176548	0	0	0	0	0.000110387459984546
unclassified(100)	Otu176584	0	0	0	0	0.000220774919969092
uncultured_bacterium(100)	Otu176672	0	0	0	0.00037448508301086	0
Aureimonas(100)	Otu176736	0	0	0	0	0.000110387459984546
Aureimonas(100)	Otu176740	0	0.000124968757810547	0	0	0
unclassified(100)	Otu176764	0	0	0	0	0.000110387459984546
unclassified(100)	Otu176819	0	0	0.000109075043630017	0	0
unclassified(100)	Otu176820	0	0	0.000109075043630017	0	0
unclassified(100)	Otu177046	0	0	0.000109075043630017	0	0
unclassified(100)	Otu177125	0	0.000124968757810547	0	0	0
unclassified(100)	Otu177149	0.0000778513040093422	0	0	0	0
unclassified(100)	Otu177152	0.0000778513040093422	0	0	0	0
unclassified(100)	Otu177154	0.0000778513040093422	0	0	0	0
Bdellovibrio(100)	Otu177535	0.000233553912028026	0	0	0	0
unclassified(100)	Otu177615	0	0	0	0	0.000110387459984546
unclassified(100)	Otu177616	0	0	0	0	0.000110387459984546
unclassified(100)	Otu177618	0	0	0	0	0.000110387459984546
unclassified(100)	Otu177646	0	0.000124968757810547	0	0	0
unclassified(100)	Otu177663	0	0	0	0.00012482836100362	0
unclassified(100)	Otu177673	0	0	0	0.00012482836100362	0
Nosocomiicoccus(100)	Otu177852	0	0	0	0.00074897016602172	0
Staphylococcus(100)	Otu178065	0	0.000124968757810547	0	0	0
Staphylococcus(100)	Otu178089	0	0.000124968757810547	0	0	0
Staphylococcus(100)	Otu178094	0	0	0	0.00012482836100362	0
Staphylococcus(100)	Otu178155	0	0.000124968757810547	0	0	0
Staphylococcus(100)	Otu178215	0	0	0.000109075043630017	0	0
Staphylococcus(100)	Otu178261	0	0	0	0.00012482836100362	0
unclassified(100)	Otu178828	0	0.000124968757810547	0	0	0
unclassified(100)	Otu178833	0	0.000124968757810547	0	0	0
unclassified(100)	Otu178888	0	0	0	0	0.000110387459984546

Stenotrophomonas(100)	Otu178889	0	0	0	0	0.000110387459984546
Stenotrophomonas(100)	Otu178896	0	0	0	0.00012482836100362	0
Stenotrophomonas(100)	Otu178897	0	0	0	0	0.000110387459984546
unclassified(100)	Otu178899	0	0	0	0	0.000110387459984546
Stenotrophomonas(100)	Otu178900	0	0	0	0	0.000110387459984546
Stenotrophomonas(100)	Otu178911	0	0	0	0	0.000110387459984546
unclassified(100)	Otu179028	0	0.000124968757810547	0	0	0
unclassified(100)	Otu179064	0	0	0	0.00012482836100362	0
Stenotrophomonas(100)	Otu179149	0	0.000124968757810547	0	0	0
Stenotrophomonas(100)	Otu179172	0	0.000124968757810547	0	0	0
Stenotrophomonas(100)	Otu179226	0.0000778513040093422	0	0	0	0
Stenotrophomonas(100)	Otu179228	0	0	0	0	0.000110387459984546
unclassified(100)	Otu179244	0	0	0.000109075043630017	0	0
Rhodanobacter(100)	Otu179249	0	0	0	0	0.000110387459984546
unclassified(100)	Otu179254	0	0	0.000109075043630017	0	0
Stenotrophomonas(100)	Otu179257	0	0	0	0	0.000110387459984546
Staphylococcus(100)	Otu179370	0	0	0	0.00012482836100362	0
Staphylococcus(100)	Otu179375	0	0	0	0.00012482836100362	0
Staphylococcus(100)	Otu179477	0	0	0	0.00012482836100362	0
Staphylococcus(100)	Otu179480	0	0	0	0.00012482836100362	0
Staphylococcus(100)	Otu179493	0	0	0.000109075043630017	0	0
Staphylococcus(100)	Otu179524	0	0.000124968757810547	0	0	0
unclassified(100)	Otu179580	0	0	0	0	0.000110387459984546
Staphylococcus(100)	Otu179725	0	0	0	0	0.000110387459984546
Staphylococcus(100)	Otu179735	0	0	0	0	0.000110387459984546
unclassified(100)	Otu179882	0	0	0	0	0.000110387459984546
unclassified(100)	Otu179883	0.0000778513040093422	0	0	0	0
unclassified(100)	Otu179884	0.0000778513040093422	0	0	0	0
unclassified(100)	Otu179887	0.0000778513040093422	0	0	0	0
unclassified(100)	Otu180008	0	0	0.000109075043630017	0	0
unclassified(100)	Otu180011	0.0000778513040093422	0	0	0	0
unclassified(100)	Otu180012	0.0000778513040093422	0	0	0	0
unclassified(100)	Otu180016	0	0.000124968757810547	0	0	0

unclassified(100)	Otu180018	0	0	0	0.00012482836100362	0
unclassified(100)	Otu180045	0	0	0	0	0.000110387459984546
unclassified(100)	Otu180073	0	0	0	0	0.000110387459984546
unclassified(100)	Otu180080	0	0	0	0	0.000110387459984546
unclassified(100)	Otu180097	0	0	0	0	0.000110387459984546
unclassified(100)	Otu180098	0	0	0	0	0.000110387459984546
unclassified(100)	Otu180099	0	0	0	0	0.000110387459984546
unclassified(100)	Otu180101	0	0	0	0	0.000110387459984546
unclassified(100)	Otu180363	0.0000778513040093422	0	0	0	0
unclassified(100)	Otu180425	0.0000778513040093422	0	0	0	0
unclassified(100)	Otu180427	0.0000778513040093422	0	0	0	0
unclassified(100)	Otu180430	0.0000778513040093422	0	0	0	0
unclassified(100)	Otu180434	0.0000778513040093422	0	0	0	0
unclassified(100)	Otu180436	0.0000778513040093422	0	0	0	0
unclassified(100)	Otu180440	0.0000778513040093422	0	0	0	0
unclassified(100)	Otu180442	0.0000778513040093422	0	0	0	0
unclassified(100)	Otu180444	0.0000778513040093422	0	0	0	0
unclassified(100)	Otu180446	0.0000778513040093422	0	0	0	0
unclassified(100)	Otu180452	0.0000778513040093422	0	0	0	0
unclassified(100)	Otu180472	0.0000778513040093422	0	0	0	0
unclassified(100)	Otu180475	0.0000778513040093422	0	0	0	0
unclassified(100)	Otu180482	0.0000778513040093422	0	0	0	0
unclassified(100)	Otu180576	0	0	0	0	0.000110387459984546
unclassified(100)	Otu180614	0.0000778513040093422	0	0	0	0
unclassified(100)	Otu180616	0.0000778513040093422	0	0	0	0
unclassified(100)	Otu180622	0.0000778513040093422	0	0	0	0
unclassified(100)	Otu180626	0.0000778513040093422	0	0	0	0
unclassified(100)	Otu180629	0.0000778513040093422	0	0	0	0
unclassified(100)	Otu180639	0.0000778513040093422	0	0	0	0
unclassified(100)	Otu180644	0.0000778513040093422	0	0	0	0
unclassified(100)	Otu180648	0.0000778513040093422	0	0	0	0
unclassified(100)	Otu180657	0	0	0.000109075043630017	0	0
unclassified(100)	Otu180658	0	0	0.000109075043630017	0	0

unclassified(100)	Otu180663	0	0	0	0.00012482836100362	0
unclassified(100)	Otu180666	0.0000778513040093422	0	0	0	0
unclassified(100)	Otu180668	0.0000778513040093422	0	0	0	0
unclassified(100)	Otu180669	0.0000778513040093422	0	0	0	0
unclassified(100)	Otu180677	0	0	0	0.00012482836100362	0
unclassified(100)	Otu180678	0	0.000124968757810547	0	0	0
unclassified(100)	Otu180679	0	0.000124968757810547	0	0	0
unclassified(100)	Otu180682	0.0000778513040093422	0	0	0	0
unclassified(100)	Otu180686	0	0.000124968757810547	0	0	0
unclassified(100)	Otu180689	0	0.000124968757810547	0	0	0
unclassified(100)	Otu180690	0	0.000124968757810547	0	0	0
unclassified(100)	Otu180696	0	0	0	0.00012482836100362	0
unclassified(100)	Otu180697	0	0	0	0.00012482836100362	0
unclassified(100)	Otu180698	0	0	0	0.00012482836100362	0
unclassified(100)	Otu180704	0	0	0.000109075043630017	0	0
unclassified(100)	Otu180710	0.0000778513040093422	0	0	0	0
unclassified(100)	Otu180714	0.0000778513040093422	0	0	0	0
unclassified(100)	Otu180716	0	0.000124968757810547	0	0	0
unclassified(100)	Otu180722	0	0	0.000109075043630017	0	0
unclassified(100)	Otu180723	0.0000778513040093422	0	0	0	0
unclassified(100)	Otu180742	0.0000778513040093422	0	0	0	0
unclassified(100)	Otu180779	0.0000778513040093422	0	0	0	0
unclassified(100)	Otu180785	0.0000778513040093422	0	0	0	0
unclassified(100)	Otu180786	0.0000778513040093422	0	0	0	0
unclassified(100)	Otu180787	0.0000778513040093422	0	0	0	0
unclassified(100)	Otu180788	0.0000778513040093422	0	0	0	0
unclassified(100)	Otu180791	0.0000778513040093422	0	0	0	0
unclassified(100)	Otu180792	0.0000778513040093422	0	0	0	0
unclassified(100)	Otu180793	0.0000778513040093422	0	0	0	0
unclassified(100)	Otu180798	0.0000778513040093422	0	0	0	0
unclassified(100)	Otu180801	0.0000778513040093422	0	0	0	0
unclassified(100)	Otu180802	0.0000778513040093422	0	0	0	0
unclassified(100)	Otu180804	0.0000778513040093422	0	0	0	0

unclassified(100)	Otu180806	0.0000778513040093422	0	0	0	0
unclassified(100)	Otu180821	0.0000778513040093422	0	0	0	0
unclassified(100)	Otu180826	0.0000778513040093422	0	0	0	0
unclassified(100)	Otu180849	0.0000778513040093422	0	0	0	0
unclassified(100)	Otu180859	0.0000778513040093422	0	0	0	0
unclassified(100)	Otu181571	0.0000778513040093422	0	0	0	0
unclassified(100)	Otu181578	0.0000778513040093422	0	0	0	0
unclassified(100)	Otu181584	0.0000778513040093422	0	0	0	0
unclassified(100)	Otu181593	0.0000778513040093422	0	0	0	0
unclassified(100)	Otu181594	0.0000778513040093422	0	0	0	0
unclassified(100)	Otu181596	0.0000778513040093422	0	0	0	0
unclassified(100)	Otu181597	0.0000778513040093422	0	0	0	0
unclassified(100)	Otu181621	0.0000778513040093422	0	0	0	0
unclassified(100)	Otu181624	0.0000778513040093422	0	0	0	0
Wolbachia(100)	Otu182995	0.0000778513040093422	0	0	0	0
Wolbachia(100)	Otu182999	0.0000778513040093422	0	0	0	0
Wolbachia(100)	Otu183000	0	0.000124968757810547	0	0	0
Wolbachia(100)	Otu183004	0.0000778513040093422	0	0	0	0
Wolbachia(100)	Otu183151	0.0000778513040093422	0	0	0	0
Wolbachia(100)	Otu183200	0	0	0	0	0.000110387459984546
Wolbachia(100)	Otu183209	0	0	0	0	0.000110387459984546
Wolbachia(100)	Otu183210	0	0	0	0	0.000110387459984546
Wolbachia(100)	Otu183214	0	0	0	0	0.000220774919969092
Wolbachia(100)	Otu183217	0	0	0	0	0.000110387459984546
Wolbachia(100)	Otu183219	0	0	0	0	0.000110387459984546
Wolbachia(100)	Otu183222	0	0	0	0	0.000110387459984546
Wolbachia(100)	Otu183279	0	0	0	0	0.000110387459984546
Wolbachia(100)	Otu183298	0	0.000124968757810547	0	0	0
Wolbachia(100)	Otu183303	0	0.000124968757810547	0	0	0
Wolbachia(100)	Otu183308	0	0.000124968757810547	0	0	0
Wolbachia(100)	Otu183310	0	0.000124968757810547	0	0	0
Wolbachia(100)	Otu183311	0	0.000124968757810547	0	0	0
Wolbachia(100)	Otu183326	0	0.000124968757810547	0	0	0

Wolbachia(100)	Otu183328	0.0000778513040093422	0	0	0	0
Wolbachia(100)	Otu183343	0	0.000124968757810547	0	0	0
Wolbachia(100)	Otu183348	0	0.000124968757810547	0	0	0
Wolbachia(100)	Otu183351	0	0.000124968757810547	0	0	0
Wolbachia(100)	Otu183352	0	0.000124968757810547	0	0	0
Wolbachia(100)	Otu183361	0	0	0.000109075043630017	0	0
Wolbachia(100)	Otu183365	0	0	0	0.00012482836100362	0
Wolbachia(100)	Otu183366	0	0	0	0.00012482836100362	0
Wolbachia(100)	Otu183374	0	0	0	0.00012482836100362	0
Wolbachia(100)	Otu183375	0	0	0	0.00012482836100362	0
Wolbachia(100)	Otu183376	0	0	0	0.00012482836100362	0
Wolbachia(100)	Otu183381	0	0	0	0.00012482836100362	0
Wolbachia(100)	Otu183384	0	0	0	0.00012482836100362	0
Wolbachia(100)	Otu183388	0	0	0	0.00012482836100362	0
Wolbachia(100)	Otu183389	0	0	0	0	0.000110387459984546
Wolbachia(100)	Otu183393	0	0	0.000109075043630017	0	0
Wolbachia(100)	Otu183394	0	0	0.000109075043630017	0	0
Wolbachia(100)	Otu183399	0	0	0.000109075043630017	0	0
Wolbachia(100)	Otu183402	0	0	0.000109075043630017	0	0
Wolbachia(100)	Otu183403	0	0	0.000109075043630017	0	0
Wolbachia(100)	Otu183404	0	0	0.000109075043630017	0	0
Wolbachia(100)	Otu183408	0	0	0.000109075043630017	0	0
Wolbachia(100)	Otu183412	0	0	0.000109075043630017	0	0
Wolbachia(100)	Otu183413	0	0	0.000109075043630017	0	0
Wolbachia(100)	Otu183414	0	0	0.000109075043630017	0	0
Wolbachia(100)	Otu183417	0	0	0.000109075043630017	0	0
Wolbachia(100)	Otu183418	0	0	0.000109075043630017	0	0
Wolbachia(100)	Otu185557	0	0.000124968757810547	0	0	0
Wolbachia(100)	Otu185793	0	0.000124968757810547	0	0	0
Wolbachia(100)	Otu185903	0	0	0.000109075043630017	0	0
Chryseobacterium(100)	Otu191170	0	0.000124968757810547	0	0	0
unclassified(100)	Otu191214	0	0	0.000109075043630017	0	0
Chryseobacterium(100)	Otu191231	0	0	0.000109075043630017	0	0

Flavobacterium(100)	Otu191234	0	0	0.000109075043630017	0	0
Chryseobacterium(100)	Otu191237	0	0	0.000109075043630017	0	0
Chryseobacterium(100)	Otu191241	0	0	0.000109075043630017	0	0
unclassified(100)	Otu191262	0	0	0.000109075043630017	0	0
Flavobacterium(100)	Otu191319	0	0	0.000109075043630017	0	0
unclassified(100)	Otu191321	0	0	0.000109075043630017	0	0
unclassified(100)	Otu191425	0	0.000124968757810547	0	0	0
Chryseobacterium(100)	Otu191436	0	0.000124968757810547	0	0	0
Chryseobacterium(100)	Otu191461	0	0.000124968757810547	0	0	0
Chryseobacterium(100)	Otu191463	0	0.000124968757810547	0	0	0
Flavobacterium(100)	Otu191468	0	0.000124968757810547	0	0	0
Flavobacterium(100)	Otu191481	0	0.000124968757810547	0	0	0
unclassified(100)	Otu191484	0	0.000124968757810547	0	0	0
Chryseobacterium(100)	Otu191507	0	0.000124968757810547	0	0	0
Chryseobacterium(100)	Otu191598	0	0	0.000109075043630017	0	0
Flavobacterium(100)	Otu191599	0	0	0.000109075043630017	0	0
Chryseobacterium(100)	Otu191600	0	0	0.000109075043630017	0	0
Flavobacterium(100)	Otu191601	0	0	0.000109075043630017	0	0
Chryseobacterium(100)	Otu191616	0	0	0.000109075043630017	0	0
Chryseobacterium(100)	Otu191622	0	0	0.000109075043630017	0	0
unclassified(100)	Otu191623	0	0	0.000109075043630017	0	0
Flavobacterium(100)	Otu191625	0	0	0.000109075043630017	0	0
unclassified(100)	Otu191626	0	0	0.000109075043630017	0	0
Chryseobacterium(100)	Otu191631	0	0	0.000109075043630017	0	0
Chryseobacterium(100)	Otu191636	0	0	0.000109075043630017	0	0
Chryseobacterium(100)	Otu191644	0	0	0	0.00012482836100362	0
unclassified(100)	Otu191645	0	0	0.000109075043630017	0	0
Chryseobacterium(100)	Otu191646	0	0	0	0.00012482836100362	0
Chryseobacterium(100)	Otu191652	0	0	0.000109075043630017	0	0
Chryseobacterium(100)	Otu191655	0	0	0.000109075043630017	0	0
unclassified(100)	Otu191657	0	0	0.000109075043630017	0	0
Chryseobacterium(100)	Otu191661	0	0	0	0.00012482836100362	0
Flavobacterium(100)	Otu191665	0	0	0	0.00012482836100362	0

Chryseobacterium(100)	Otu191668	0	0	0	0.00012482836100362	0
unclassified(100)	Otu191669	0	0	0	0.00012482836100362	0
Chryseobacterium(100)	Otu191674	0	0	0	0.00012482836100362	0
unclassified(100)	Otu191675	0	0	0	0.00012482836100362	0
Chryseobacterium(100)	Otu191685	0	0	0	0.00012482836100362	0
Flavobacterium(100)	Otu191688	0	0	0	0.00012482836100362	0
Chryseobacterium(100)	Otu191695	0	0	0.000109075043630017	0	0
Chryseobacterium(100)	Otu191696	0	0	0.000109075043630017	0	0
Flavobacterium(100)	Otu191699	0	0	0.000109075043630017	0	0
Flavobacterium(100)	Otu191700	0	0	0.000109075043630017	0	0
Flavobacterium(100)	Otu191706	0	0	0.000109075043630017	0	0
Chryseobacterium(100)	Otu191707	0	0	0.000109075043630017	0	0
Chryseobacterium(100)	Otu191713	0	0	0	0.00012482836100362	0
Chryseobacterium(100)	Otu191720	0	0	0	0.00012482836100362	0
Flavobacterium(100)	Otu191799	0	0	0	0.00012482836100362	0
Bergeyella(100)	Otu191802	0	0	0	0.00012482836100362	0
Chryseobacterium(100)	Otu191804	0	0	0	0.00012482836100362	0
Chryseobacterium(100)	Otu191807	0	0	0.000109075043630017	0	0
Flavobacterium(100)	Otu191810	0	0	0.000109075043630017	0	0
Flavobacterium(100)	Otu191811	0	0	0.000109075043630017	0	0
Chryseobacterium(100)	Otu191816	0	0	0.000109075043630017	0	0
Chryseobacterium(100)	Otu191818	0	0	0.000109075043630017	0	0
Flavobacterium(100)	Otu191819	0	0	0.000109075043630017	0	0
Chryseobacterium(100)	Otu191825	0	0	0.000109075043630017	0	0
Cloacibacterium(100)	Otu191829	0	0	0.000109075043630017	0	0
Chryseobacterium(100)	Otu191830	0	0	0.000109075043630017	0	0
Chryseobacterium(100)	Otu191832	0	0	0.000109075043630017	0	0
Flavobacterium(100)	Otu191997	0	0	0	0.00012482836100362	0
Chryseobacterium(100)	Otu192006	0	0	0	0.00012482836100362	0
Chryseobacterium(100)	Otu192007	0	0	0	0.00012482836100362	0
Chryseobacterium(100)	Otu192012	0	0	0	0.00012482836100362	0
Flavobacterium(100)	Otu192026	0	0	0	0.00012482836100362	0
unclassified(100)	Otu192030	0	0	0	0.00012482836100362	0

Chryseobacterium(100)	Otu192035	0	0	0	0.00012482836100362	0
Chryseobacterium(100)	Otu192053	0	0	0	0.00012482836100362	0
Chryseobacterium(100)	Otu192054	0	0	0	0.00012482836100362	0
Flavobacterium(100)	Otu192090	0	0	0	0.00012482836100362	0
Flavobacterium(100)	Otu192092	0	0	0	0.00012482836100362	0
Chryseobacterium(100)	Otu192096	0	0	0	0.00012482836100362	0
Chryseobacterium(100)	Otu192099	0	0	0	0.00012482836100362	0
Flavobacterium(100)	Otu192728	0	0.000124968757810547	0	0	0
Flavobacterium(100)	Otu192770	0	0.000124968757810547	0	0	0
Chryseobacterium(100)	Otu192776	0	0.000124968757810547	0	0	0
Chryseobacterium(100)	Otu192837	0	0.000124968757810547	0	0	0
Chryseobacterium(100)	Otu192842	0	0.000124968757810547	0	0	0
unclassified(100)	Otu192844	0	0.000124968757810547	0	0	0
unclassified(100)	Otu192845	0	0.000124968757810547	0	0	0
Chryseobacterium(100)	Otu192846	0	0.000124968757810547	0	0	0
Chryseobacterium(100)	Otu192853	0	0.000124968757810547	0	0	0
unclassified(100)	Otu192865	0	0.000124968757810547	0	0	0
unclassified(100)	Otu193071	0	0.000124968757810547	0	0	0
Chryseobacterium(100)	Otu193106	0.0000778513040093422	0	0	0	0
Chryseobacterium(100)	Otu193116	0.0000778513040093422	0	0	0	0
Chryseobacterium(100)	Otu193123	0	0.000124968757810547	0	0	0
Chryseobacterium(100)	Otu193262	0	0	0	0	0.000110387459984546
Flavobacterium(100)	Otu193267	0	0	0	0	0.000110387459984546
Chryseobacterium(100)	Otu193269	0	0	0	0	0.000110387459984546
Flavobacterium(100)	Otu193275	0	0	0	0	0.000110387459984546
Flavobacterium(100)	Otu193277	0	0	0	0	0.000110387459984546
Flavobacterium(100)	Otu193290	0	0	0	0.00012482836100362	0
Chryseobacterium(100)	Otu193313	0	0	0	0.00012482836100362	0
unclassified(100)	Otu193935	0	0	0	0	0.000110387459984546
Chryseobacterium(100)	Otu193971	0	0	0	0.00012482836100362	0
unclassified(100)	Otu193981	0	0	0	0.00012482836100362	0
Flavobacterium(100)	Otu194027	0	0	0	0	0.000110387459984546
unclassified(100)	Otu194029	0	0	0	0	0.000110387459984546

Chryseobacterium(100)	Otu194030	0	0	0	0	0.000110387459984546
unclassified(100)	Otu194033	0	0	0	0	0.000110387459984546
Flavobacterium(100)	Otu194035	0	0	0	0	0.000110387459984546
Chryseobacterium(100)	Otu194040	0	0	0	0	0.000110387459984546
Chryseobacterium(100)	Otu194055	0	0	0	0	0.000110387459984546
Chryseobacterium(100)	Otu194061	0	0	0	0	0.000110387459984546
Flavobacterium(100)	Otu194066	0	0	0	0	0.000110387459984546
Chryseobacterium(100)	Otu194070	0	0	0	0	0.000110387459984546
Chryseobacterium(100)	Otu194071	0	0	0	0	0.000110387459984546
unclassified(100)	Otu194113	0	0	0	0	0.000110387459984546
Chryseobacterium(100)	Otu194119	0	0	0	0	0.000110387459984546
Chryseobacterium(100)	Otu194128	0	0	0	0	0.000110387459984546
Chryseobacterium(100)	Otu194130	0	0	0	0	0.000110387459984546
Flavobacterium(100)	Otu194134	0	0	0	0	0.000110387459984546
unclassified(100)	Otu194135	0	0	0	0	0.000110387459984546
Flavobacterium(100)	Otu196713	0	0.000124968757810547	0	0	0
Chryseobacterium(100)	Otu196715	0	0.000124968757810547	0	0	0
Flavobacterium(100)	Otu196719	0	0.000124968757810547	0	0	0
Chryseobacterium(100)	Otu196732	0	0.000124968757810547	0	0	0
Bergeyella(100)	Otu196738	0	0.000124968757810547	0	0	0
Chryseobacterium(100)	Otu196762	0	0.000124968757810547	0	0	0
unclassified(100)	Otu196773	0	0.000124968757810547	0	0	0
Bergeyella(100)	Otu196777	0	0.000124968757810547	0	0	0
Myroides(100)	Otu196778	0	0.000124968757810547	0	0	0
Chryseobacterium(100)	Otu196780	0	0.000124968757810547	0	0	0
unclassified(100)	Otu196795	0	0.000124968757810547	0	0	0
Chryseobacterium(100)	Otu196810	0	0.000124968757810547	0	0	0
Chryseobacterium(100)	Otu196812	0	0.000124968757810547	0	0	0
Chryseobacterium(100)	Otu196825	0	0.000124968757810547	0	0	0
Chryseobacterium(100)	Otu196855	0	0.000124968757810547	0	0	0
Chryseobacterium(100)	Otu196867	0	0.000124968757810547	0	0	0
Chryseobacterium(100)	Otu196873	0	0.000124968757810547	0	0	0
Flavobacterium(100)	Otu196878	0	0.000124968757810547	0	0	0

Flavobacterium(100)	Otu196884	0	0.000124968757810547	0	0	0
Chryseobacterium(100)	Otu196900	0	0.000124968757810547	0	0	0
unclassified(100)	Otu196916	0	0.000124968757810547	0	0	0
Chryseobacterium(100)	Otu196920	0	0.000124968757810547	0	0	0
unclassified(100)	Otu196921	0	0.000124968757810547	0	0	0
unclassified(100)	Otu196923	0	0.000124968757810547	0	0	0
Chryseobacterium(100)	Otu196938	0	0.000124968757810547	0	0	0
Chryseobacterium(100)	Otu196941	0	0.000124968757810547	0	0	0
